# Modulation of the Cytotoxic Properties of Pd(II) Complexes Based on Functionalized Carboxamides Featuring Labile Phosphoryl Coordination Sites

**DOI:** 10.3390/pharmaceutics15041088

**Published:** 2023-03-28

**Authors:** Diana V. Aleksanyan, Aleksandr V. Konovalov, Svetlana G. Churusova, Ekaterina Yu. Rybalkina, Alexander S. Peregudov, Svetlana A. Aksenova, Evgenii I. Gutsul, Zinaida S. Klemenkova, Vladimir A. Kozlov

**Affiliations:** 1 A. N. Nesmeyanov Institute of Organoelement Compounds, Russian Academy of Sciences, ul. Vavilova 28, Str. 1, Moscow 119334, Russia; 2 Department of Chemistry and Technology of Organic Synthesis, Faculty of Chemical Pharmaceutical Technologies and Biomedical Preparations, Russian University of Chemical Technology, Miusskaya pl. 9, Moscow 125047, Russia; 3 N. N. Blokhin National Medical Research Center of Oncology of the Ministry of Health of the Russian Federation, Kashirskoe Shosse 23, Moscow 115478, Russia; 4 Moscow Institute of Physics and Technology (National Research University), Institutskiy per. 9, Dolgoprudny, Moscow 141700, Russia

**Keywords:** palladium, (aminoalkyl)phosphine oxides, bi- and tridentate ligands, anticancer activity, metal-based cytotoxic agents

## Abstract

Platinum-based drugs are commonly recognized as a keystone in modern cancer chemotherapy. However, intrinsic and acquired resistance as well as serious side effects often caused by the traditional Pt(II) anticancer agents prompt a continuous search for more selective and efficient alternatives. Today, significant attention is paid to the compounds of other transition metals, in particular those of palladium. Recently, our research group has suggested functionalized carboxamides as a useful platform for the creation of cytotoxic Pd(II) pincer complexes. In this work, a robust picolinyl- or quinoline-carboxamide core was combined with a phosphoryl ancillary donor group to achieve hemilabile coordination capable of providing the required level of thermodynamic stability and kinetic lability of the ensuing Pd(II) complexes. Several cyclopalladated derivatives featuring either a bi- or tridentate pincer-type coordination mode of the deprotonated phosphoryl-functionalized amides were selectively synthesized and fully characterized using IR and NMR spectroscopy as well as X-ray crystallography. The preliminary evaluation of the anticancer potential of the resulting palladocycles revealed a strong dependence of their cytotoxic properties on the binding mode of the deprotonated amide ligands and demonstrated certain advantages of the pincer-type ligation.

## 1. Introduction

**Figure 1:**
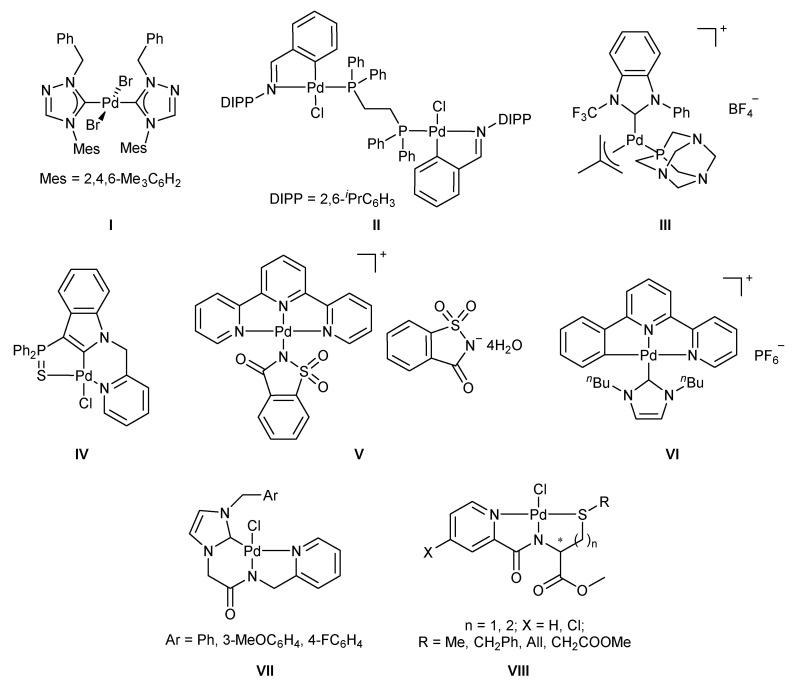
Selected examples of Pd(II) complexes exhibiting anticancer properties [24,25,26,27,28,29,30,31,32].

The introduction of cisplatin into clinical practice in 1978 became a landmark event in the development of chemotherapy which, until that time, had been a domain of only organic compounds. This led to a surge of investigations on the anticancer potential of different types of Pt(II) compounds; these subsequently gave rise to several next-generation platinum-based chemotherapeutics, including carboplatin, oxaliplatin, lobaplatin, nedaplatin, and heptaplatin [1]. In the last decade, research in this field has been markedly advanced by the creation of targeted Pt(II) agents, Pt(IV) prodrugs, and nanoparticle delivery systems [2,3,4,5,6,7,8,9]; the prominent results have been demonstrated by the combination therapy [10,11,12]. However, despite the crucial role of platinum-based drugs in current cancer treatment, their application is often associated with the appearance of resistance and systemic toxicity that results in severe side effects [13]. Looking for alternatives to platinum compounds, many research groups around the world are actively exploring the anticancer properties of other transition metal derivatives [14,15,16,17,18]. Particular attention is drawn to organometallic and metal-organic compounds of palladium [4,15,19,20,21,22]. The basic premise of investigations in this area is that Pd(II) complexes show coordination behaviors similar to their Pt(II) counterparts; but the major difference lies in the much faster ligand-exchange processes that can lead to the undesired deactivation of potential Pd(II) drugs in the biological environment [23]. This latter fact has provoked the development of promising palladium-based candidates that have gone far beyond the classical Pt(II) anticancer agents both structurally and mechanistically (see, for example, compounds **I**–**VIII** in Figure 1 [24,25,26,27,28,29,30,31,32]).

One of the successful approaches to the creation of novel palladium(II) cytotoxic agents is based on the application of chelating ligands that can undergo cyclometalation [19,20]. The optimal balance between the thermodynamic and kinetic stability of various cyclopalladated species has been generally recognized for catalytic purposes [33,34], and this strategy is now gaining popularity in medicinal chemistry. The particularly encouraging results from several research groups, including our own, have been recently achieved with the so-called pincer-type ligands that feature highly tunable monoanionic tridentate frameworks (e.g., compounds **IV**, **VI**–**VIII** in Figure 1) [27,29,30,31,32]. At the same time, to the best of our knowledge, there have been no direct comparative investigations on the effect of a pincer vs. bidentate coordination mode on the anticancer activity of cyclopalladated derivatives. To fill this gap, we have designed new representatives of non-classical functionalized amide ligands that combine a robust picolinylamide core with labile phosphoryl coordination arms. The presence of the latter ensured the production of closely related mono- and bis(palladocyclic) (pincer) complexes. The following bioactivity studies disclosed the modulation of their cytotoxic properties in strict compliance with the binding mode of the deprotonated amide ligands and the superiority of the pincer-type ligation.

## 2. Results and Discussion

**Scheme 1:**
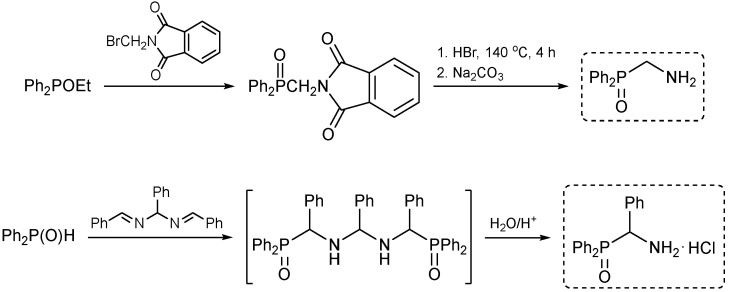
Synthesis of the key phosphorylated amine precursors.

The *N*,*N*-chelating motif is widely recognized as highly effective for generating cytotoxic complexes of both platinum and non-platinum metals. Analogously, the chloride anion and various oxygen donor centers are often used as appropriate leaving groups. Our strategy for the design of new palladium-based chemotherapeutics aims to integrate these basic principles with the pincer concept to achieve higher tunability of the Pd(II) coordination environment. It is noteworthy that a combination of the firmly coordinating deprotonated functionalized amide unit with a more labile ancillary donor group in a single tridentate ligand framework has already proved successful in the case of the Pd(II) pincer complexes based on (homo)cysteine and methionine derivatives [31,32,35,36], (methylsulfanyl)acetic and propionic acid derivatives [37,38], and monothiooxamides [39]. In this work, the phosphoryl group featuring a hard oxygen donor atom was chosen to ensure sufficient hemilability of the resulting ligand system, which would enable, in turn, the synthesis of target complexes with both a tridentate binding mode and a bidentate coordination. Previously, we demonstrated the utility of *o*-phosphorylated aniline and its thio analog for obtaining the biologically and catalytically active Pd(II) complexes with non-classical amide-based pincer scaffolds (see [38] and the articles cited therein). However, switching to aliphatic amines was expected to provide a higher flexibility degree of the ligand framework. For this purpose, (aminomethyl)diphenylphosphine oxide was synthesized by the Michaelis–Arbuzov reaction between Ph_2_POEt and *N*-bromomethylphthalimide, followed by the hydrolysis of the protecting imide moiety according to the published procedure (Scheme 1) [40]. The treatment of hydrobenzamide with diphenylphosphine oxide generated in situ from Ph_2_PCl afforded a hydrochloride salt of its analog with an additional phenyl substituent in the bridging unit between the phosphoryl and amine groups, which is able to impart an additional steric effect (Scheme 1) [41].

**Scheme 2:**
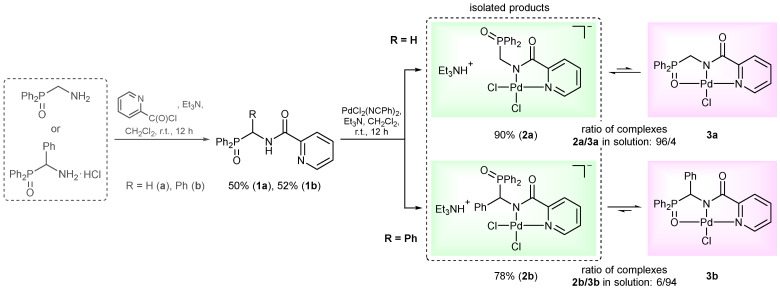
Synthesis and cyclopalladation of the phosphoryl-functionalized picolinylamides.

**Figure 2:**
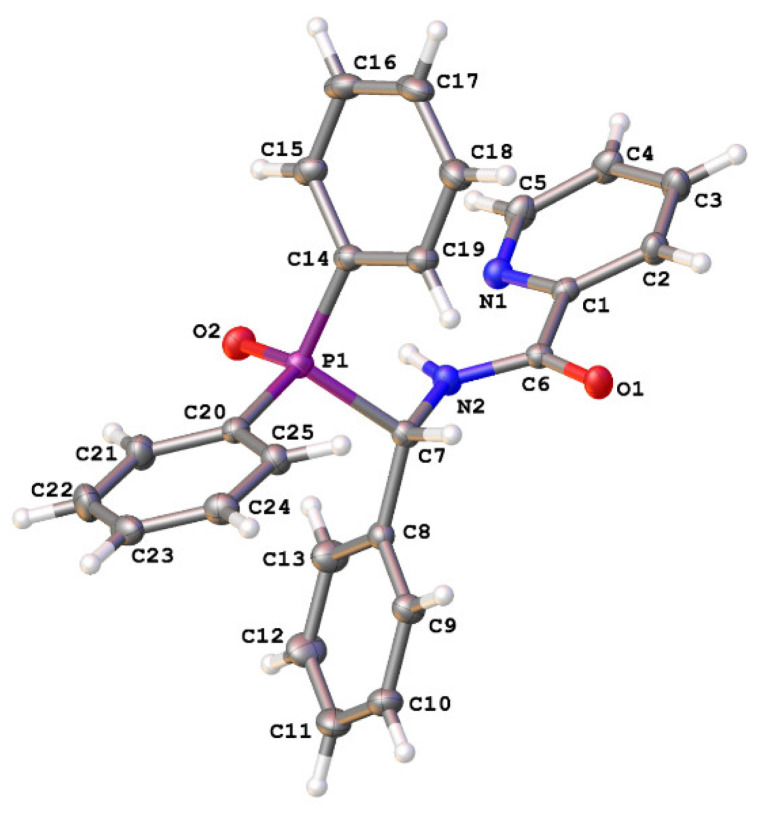
Molecular structure of ligand **1b**. Hereinafter, the atoms are drawn as thermal ellipsoids at 40% probability level.

The reactions of the key phosphorylated amine precursors with picolinyl chloride smoothly afforded the target functionalized amide ligands (compounds **1a**,**b**, Scheme 2). Their structures and compositions were unambiguously confirmed by the multinuclear NMR and IR spectroscopic data as well as elemental analyses (see the experimental section and Figures S1–S10 in the Supporting Information (SI) for a full set of the NMR and IR spectra of ligand **1a** used as a representative example). The molecular structure of ligand **1b** was also corroborated using X-ray crystallography (Figure 2).

**Scheme 3:**
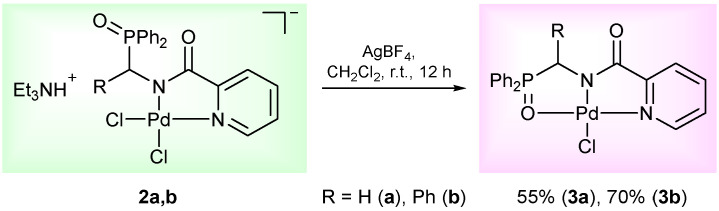
Synthesis of Pd(II) pincer complexes of the phosphorylated picolinylamides.

The complexing features of the resulting functionalized picolinylamides towards Pd(II) ions were studied through their interaction with PdCl_2_(NCPh)_2_, which is commonly used as a versatile cyclopalladating agent. The reactions were performed under mild conditions, in dichloromethane at room temperature in the presence of Et_3_N. The latter was necessary for trapping HCl liberated during metalation in order to prevent the possible ligand deactivation. Although the subsequent ex situ analysis of the isolated products confirmed the presence of an *N*,*N*-chelated moiety, i.e., the occurrence of cyclometalation in the case of both ligands **1a**,**b** (vide infra), the ^31^P NMR monitoring of the reaction course revealed a significant difference in the coordination behavior of the phosphorus ancillary donor groups of these compounds in solution. The major signal in the ^31^P NMR spectrum of the reaction mixture with (diphenylphosphoryl)methyl-appended ligand **1a** (*δ*_P_ = 31.1 ppm) appeared in the region characteristic of free tertiary phosphine oxides. The minor signal at 72.3 ppm was indicative of the strong coordination of the P=O donor group and was presumably assigned to a pincer-type product. In the case of the phenyl-substituted analog (ligand **1b**), an opposite spectral pattern implied the predominance of the phosphoryl-coordinated species (the ratio of the signals at 72.7 and 31.9 ppm was 94/6). But despite this, the only isolated solid products from both reaction mixtures were anionic palladate complexes **2a**,**b** featuring a bidentate coordination mode of the deprotonated amide ligands and Et_3_NH^+^ counter ions (Scheme 2). Nevertheless, when dissolved, these complexes completely reproduced the spectral features that had already been observed for the initial reaction mixtures. This implies the existence of equilibrium between the derivatives bound in the bi- and tridentate fashion in solution. More importantly, the ratio of the latter strongly depended on the steric properties of the phosphoryl coordination arms and was selectively shifted either to the bidentate complex (in the case of **1a**) or to the pincer-type counterpart (in the case of **1b**). In fact, we have achieved the desired lability of the ligand framework, which was realized, in addition, in a highly selective manner. As for obtaining the target pincer-type complexes in the pure form, this was readily accomplished by the chloride abstraction from **2a**,**b** under the action of AgBF_4_ (compounds **3a**,**b**, Scheme 3).

The resulting complexes were exhaustively characterized using IR and multinuclear NMR spectroscopy (including different 2D NMR techniques) as well as elemental analysis. The lack of C(O)NH proton signals in the ^1^H NMR spectra unequivocally testified to the deprotonation of the central amide unit in all cases. This was accompanied by a strong downfield shift of the C=O carbon resonance (Δ*δ*_C_ = 5.61–7.22 ppm). The analogous changes were observed in the IR spectra of solid complexes **2a**,**b** and **3a**,**b**: the absorption bands associated with the NH stretching and bending motions (observed at 3392/1515 and 3363/1513 cm^–1^ for ligands **1a** and **1b**, respectively) disappeared, whereas the carbonyl stretches notably shifted to the lower frequencies (Δ*ν* = 36–57 cm^–1^). The *N*,*N*-chelation was indirectly supported by the expected changes in the resonances of some hydrogen and carbon nuclei of the pyridine core. For example, the signal of the CH proton closest to the heteroatom was found to be downfield shifted by 0.35–0.60 ppm (the greatest difference was observed in the case of complex **2a**). In turn, the redistribution of electron density in the amide unit led to a significant downfield shift of the signal of the *ipso*-C pyridine nucleus, reaching up to 7.47 ppm. Finally, the convincing evidence for the coordination of both pyridine and amide units was provided by the results of ^1^H–^15^N HMBC analysis. Thus, the amide nitrogen resonances of **2a** and **3a** were found to be downfield shifted relative to the signal of free ligand **1a** by 26.1 and 22.4 ppm, respectively, while the signals of the pyridine nitrogen nuclei shifted in the opposite direction by 87.8 (**2a**) and 103.2 (**3a**) ppm. Note that the complete peak assignments for most of the compounds explored was performed based on ^1^H–^1^H COSY, ^1^H–^13^C HSQC, and ^1^H–^13^C HMBC spectra. For illustration, the NMR spectra of cyclopalladated derivatives **2a** and **3b**, along with their IR spectra, are provided in the SI (Figures S11–S36).

The strongly deshielded phosphorus resonances in the ^31^P NMR spectra of complexes **3a**,**b** clearly indicated the coordination of the phosphoryl donor groups (Δ*δ*_P_ reached up to 43.2 ppm), confirming the realization of a pincer-type ligation in these cases. The same was also observed for complex **2b**, which, upon dissolution, almost completely converts to the P(O)-coordinated product: compare *δ*_P_ = 73.92 ppm for a solution of **2b** in CDCl_3_ with the phosphorus resonance of an authentic sample of **3b** in CDCl_3_ (73.89 ppm). Interestingly, the ^1^H and ^13^C NMR spectra of this palladocycle show a double set of signals (besides the nonequivalent signals of prochiral groupings such as Ph substituents at the phosphorus atom) that correspond to two isomeric pincer complexes (see Figures S37–S44 in the SI). The latter are likely to arise due to fixation of the chiral CHPh unit in space upon closure of the second metal-containing ring as a result of the P=O group coordination, which, in the case of complex **2b**, is reversible. An additional signal at ca. 39.6 ppm in the ^31^P NMR spectrum of palladocycle **3a** may result from the partial decoordination of the P=O arm (slightly broadened and poorly resolved signals in the ^1^H NMR spectrum of this palladocycle also argue for the existence of dynamic transformations in solution) (Figures S45 and S46 in the SI). In contrast, the bidentately bound derivative is the major form of **2a** in solution, which corresponds to the signal at 34.17 ppm (*cf. δ*_P_ = 30.18 ppm for free ligand **1a**). As for the structures of these complexes in the solid state, the IR spectra unambiguously confirmed the proposed bi- (**2a**,**b**) and tridentate (**3a**,**b**) coordination mode of the deprotonated amide ligands. Thus, the binding of the ancillary phosphoryl donor groups in the pincer-type complexes resulted in an essential shift of the P=O stretches when compared to the corresponding absorption bands in the spectra of free ligands **1a**,**b** (Δ*ν* = 70 (**3a**) and 77 (**3b**) cm^–1^). In the case of cyclopalladated derivatives **2a**,**b**, this shift reached maximum 27 cm^–1^ and was due to the hydrogen bonding between the P=O group and the ammonium cation (vide infra).

**Figure 3:**
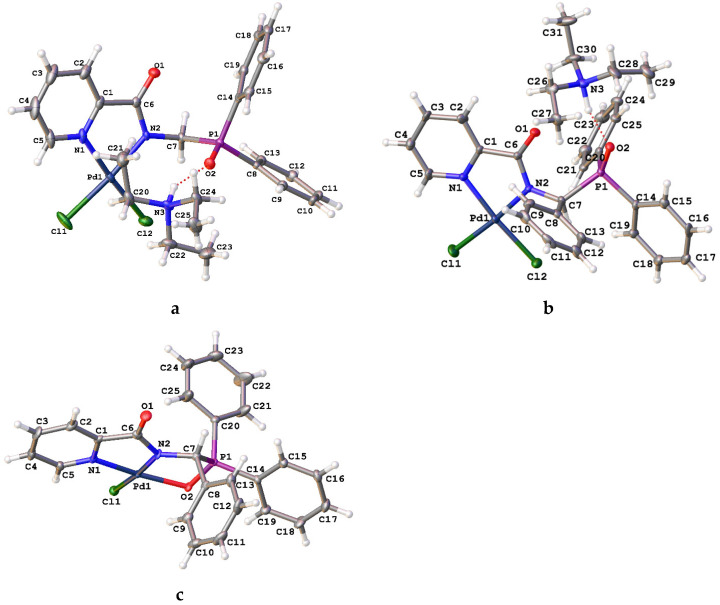
Molecular structures of complexes **2a** (**a**), **2b** (**b**), and **3b** (**c**). The solvate chloroform molecule in **2b** is omitted for clarity. Dotted lines stand for hydrogen bonds.

The structures of complexes **2a**,**b** and **3b** in the solid state were further supported by the results of XRD analysis (Figure 3). Table 1 lists some important bond lengths and angles for these cyclopalladated derivatives and ligand **1b**. As anticipated, in compounds **2a**,**b** the palladium ion is coordinated by two nitrogen atoms of the deprotonated picolinylamide unit and two chloride ligands. The resulting complex anions are bound with triethylammonium cations through hydrogen bonds between the P=O group and NH^+^ moiety (N…O 2.715(3) Å, NHO 149.24(14)° (**2a**), N…O 2.776(7) Å, NHO 160.4(5)° (**2b**)). The formation of the ionic pairs in **2b** is also assisted by C–H…O contacts between the C=O group of the anion and CH_2_ group of the cation (C…O–3.171(10) Å, CHO 122.9(5)°). In **3b**, the deprotonated amide ligand adopts a tridentate coordination mode, additionally binding with the metal center through the oxygen atom of the phosphoryl group. One chloride ligand completes the coordination sphere of the Pd(II) ion. The more diversified environment leads to the more distorted square-planar geometry around the metal center in pincer complex **3b** compared to its monometallocyclic counterparts **2a**,**b**, although the main geometric parameters that involve coordination bonds in these compounds are quite close and lie within the expected ranges. The coordination of the phosphoryl group in **3b** results in a significant elongation of the P=O bond (1.536(2) Å vs. 1.4843(11) Å in free ligand **1b**). The *N*,*N*-chelation in complex **2b** only slightly affects the bonding parameters of the picolinylamide unit, whereas in the case of pincer complex **3b**, the marked changes are observed for both N1–C1 bond in the pyridine ring and C1–C6 bond between the carbonyl group and heterocyclic moiety. This is likely to be connected with the presence of a system of two fused metallocycles which, in turn, adopt envelope conformations (with atoms Pd1 and P1 deviating by 0.283(5) and 0.618(3) Å from the mean planes of other atoms in the *N*,*N*- and *O*,*N*-chelate rings, respectively), unlike the planar metal-containing cycles in complexes **2a**,**b**. In the crystal of **2a**, the C=O group forms C–H…O contacts with one of the phenyl substituents and CH_2_ group (C…O 3.451(3)–3.548(3) Å, CHO 146.56(14)–157.54(15)°) to produce centrosymmetric dimers; the formation of the 3D-framework is completed by weaker van der Waals contacts. In the case of palladocycle **2b**, the pyridine units form parallel-displaced stacking interactions (with the interplane angle of 0° and the inter-centroid and shift distances of 3.7162(6) and 1.720(13) Å, respectively) that pack the anions into centrosymmetric dimers; those are held together by weaker van der Waals contacts to produce a 3D-framework. In the crystal of pincer complex **3b**, both the P=O and C=O groups form C–H…O contacts with the hydrogen atoms of one (C…O 3.264(4) Å, CHO 137.2(2)°) and two (C…O 3.257(4)–3.315(4) Å, CHO 142.4(2)–160.3(2)°) phenyl substituents, respectively. The resulting zigzag chains along the crystallographic axis *c* are held together by weaker van der Waals contacts, creating a 3D-framework. The fragments of the crystal packing of the complexes explored are depicted in Figure S47 in the SI.

To characterize the antitumor potential of the resulting Pd(II) pincer complexes, their cytotoxicities against a panel of human solid and hematopoietic cancer cell lines, including colorectal carcinoma (HCT116), breast cancer (MCF7), prostate adenocarcinoma (PC3), glioblastoma (U251), ovarian adenocarcinoma (Scov3), chronic myelogenous leukemia (K562) and its resistant subclone (K562/iS9), multiple plasmacytoma (AMO1), and acute lymphoblastic leukemia (H9) cell lineages, were evaluated using the conventional MTT assay. The results obtained are presented in Table 2 and Table 3 as the concentrations required for inhibiting the cellular survival fraction to 50% (IC_50_) defined after an exposure time of 48 h. For comparison, the inhibitory effects of the compounds explored on noncancerous human embryonic kidney cells HEK293 as well as transformed breast cells HBL100 and their doxorubicin-resistant analogs HBL100/Dox were also investigated under the same conditions.

In general, the complexes derived from (diphenylphosphoryl)methyl-appended ligand **1a** (compounds **2a** and **3a**, entries 2 and 4, respectively, in Table 2 and Table 3) were only moderately cytotoxic to some solid and all hematopoietic cancer cell lines and exhibited comparable activity towards noncancerous cells HEK293 (although they did not affect mammary epithelial cells HBL100). Their counterparts based on the phosphoryl-functionalized picolinylamide bearing an additional phenyl substituent (complexes **2b** and **3b**, entries 3 and 5) demonstrated almost the same efficiency on U251 and Scov3 cells but appeared to be significantly more toxic towards other cancer lineages explored, in most cases surpassing in the activity the classical metal-based anticancer agent cisplatin used as a reference (entry 7 in Table 2 and entry 6 in Table 3). Palladocycles **2b** and **3b** exhibited a particularly high level of antiproliferative activity against human colon cancer cells HCT116, with IC_50_ values falling in the low micromolar range (3–4 μM), and markedly lower toxicity towards HEK293 and HBL100 cells. As for the difference in the activities of the bi- and tridentate derivatives, it was almost negligible for both pairs of the complexes in the experiments with solid cancer cells but became apparent for more sterically hindered derivatives **2b** and **3b** on the hematopoietic cell lines. Thus, pincer-type complex **3b** essentially outperformed its monopalladocyclic analog **2b** on K562, AMO1, and H9 cell lineages (compare entries 3 and 5 in Table 3).

The observed dependences correlate well with our previous findings on the lability of the phosphoryl sites in palladocycles **2a**,**b**, **3a**,**b** and can be rationalized in terms of their coordination behavior. Thus, complex **2a**, which tends to retain the *N*,*N*-bidentate coordination mode of the deprotonated amide ligand in solution, exhibits lower activity than its counterpart **2b**, for which the pincer-type ligation is preferred. This latter fact may also explain why the cytotoxic effects of palladocycle **2b** are comparable in some cases to those of pincer-type complex **3b** based on the same phosphorylated ligand. However, the presence of an additional competitive chloride anion makes complex **2b** potentially more susceptible to decoordination of the phosphoryl arm. Confirming this assumption, the ^31^P NMR studies in CDCl_3_–(CD_3_)_2_SO mixture revealed that complex **2b** produces about 30% of decoordinated species already after dissolution, while pincer-type palladocycle **3b** is quite stable in this medium and reaches the commensurable decomposition degree only in a week (Figures S48 and S49 in the SI). The additional investigations by UV-vis spectroscopy revealed high stability of complexes **2a** and **3a** (used as representative examples) in neat DMSO as well as in DMSO–water and DMSO–PBS solutions (see Figure S50 in the SI). At least the *N*,*N*-bidentately bound core remained intact in the mentioned media over a period of 48 h. In turn, the stability of the amide-based complexes under consideration towards cell culture medium was indirectly confirmed by the high levels of cytotoxic activity of palladocycles **2b** and **3b**, preliminarily kept in DMSO–RPMI 1640 mixture (1/10 by volume) for 48 h before the experiments on AMO1 and K562 cells; these appeared to be comparable to the cytotoxicity of these complexes dissolved in neat DMSO (Figure S51 in the SI).

**Scheme 4:**
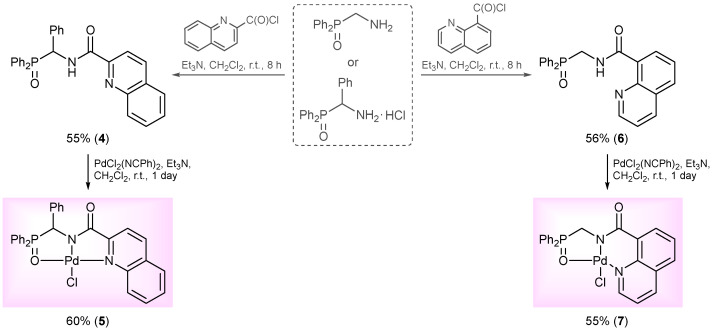
Synthesis of the isomeric phosphoryl-functionalized quinoline-substituted carboxamides and their Pd(II) pincer complexes.

**Figure 4:**
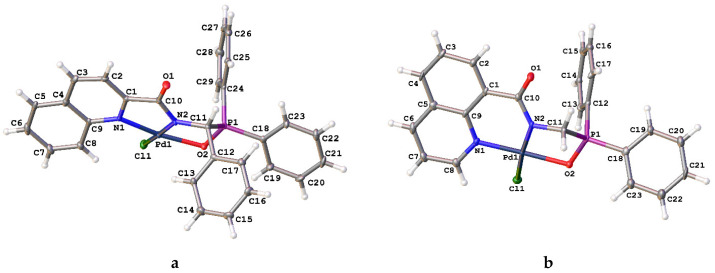
Molecular structures of complexes **5** (**a**) and **7** (**b**). The solvate dichloromethane (**5**) or chloroform (**7**) molecules as well as the second symmetry-independent molecule of complex **7** are omitted for clarity. For the selected bond lengths and angles, see Table S1 in the SI.

To further explore the effect of lability of coordination sites on the biological activity of this type of cyclopalladated complexes, we decided to modify the second arm in the *O*,*N*,*N*-ligand framework, specifically the ancillary *N*-donor group, replacing the pyridine unit for a more rigid quinoline moiety. The reaction of [amino(phenyl)methyl]diphenylphosphine oxide hydrochloride with in situ generated quinoline-2-carboxylic acid chloride smoothly furnished functionalized amide **4**, which, in turn, readily underwent direct cyclopalladation, affording Pd(II) pincer complex **5** (Scheme 4). The molecular structure of this palladocycle is presented in Figure 4, while its main geometric parameters are listed in Table S1 in the SI. Unfortunately, complex **5** appeared to be insoluble in common organic solvents and unstable in strongly coordinating media (e.g., DMSO); a possible reason for the stability issues is its highly constrained structure. Therefore, it was withdrawn from the cytotoxicity studies. An isomeric analog of complex **5** based on phosphoryl-substituted quinoline-8-carboxamide **6** and bearing fused metallocycles of different sizes (compound **7**, Scheme 4; for the results of XRD study, see Figure 4 and Table S1 in the SI) was stable in DMSO but displayed low activity towards HCT116, MCF7, and PC3 cancer cell lines, simultaneously affecting noncancerous cells HEK293 to a greater extent. Hence, a combination of the pyridine and phosphoryl donor groups provides an optimal level of the framework flexibility, where additional steric effects in the P=O coordination arm ensure more stable pincer-type ligation which seems to be favorable for improved cytotoxic properties.

It is important to mention that free ligand **1a** appeared to be almost nontoxic even at concentrations as high as 80–100 μM (entry 1 in Table 2 and Table 3). This allows us to conclude that the cytotoxic properties of the cyclopalladated derivatives under consideration are primarily determined by the coordination with Pd(II) ions.

**Figure 5:**
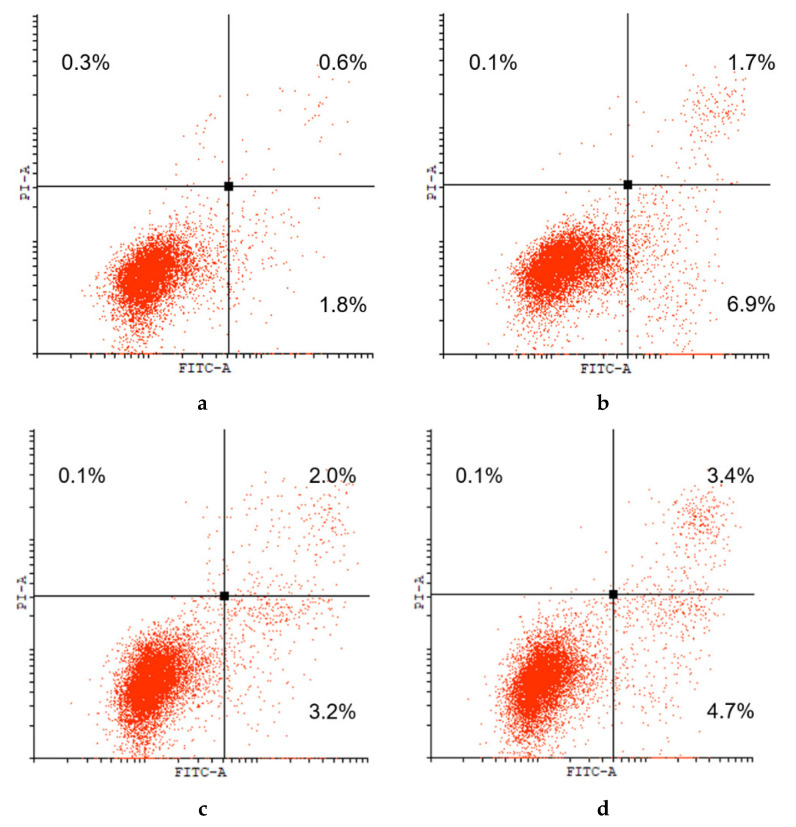
Percentages of necrotic (upper left), early apoptotic (lower right), and late apoptotic (upper right) K562 (**a**,**b**) and K562/iS9 (**c**,**d**) cells in the control experiments (**a**,**c**) and after exposure to complex **3b** (**b**,**d**) for 20 h.

Finally, the comparable levels of cytotoxic activity of most of the complexes obtained in this study against the parental cell lines HBL100 and K562 and their doxorubicin-resistant subclones HBL100/Dox and K562/iS9 show the prospects of the development of new anticancer agents based on the related derivatives that would be able to circumvent drug resistance. This is also confirmed by the results of flow cytometric studies on apoptosis inducing ability of the most active palladocycle (complex **3b**), performed using the Annexin V-FITC/PI double staining assay at the compound concentration of 10 μM. The diagrams presented in Figure 5 show that the total percentages of early (lower right quadrant) and late (upper right quadrant) apoptotic cells were almost the same for parental cells K562 and their resistant analogs K562/iS9. This suggests that the cyclopalladated complexes of phosphoryl-functionalized carboxamides represent promising objects for further detailed investigations of their anticancer potential.

## 3. Conclusions

To summarize the results presented, the phosphoryl-functionalized picolinylamides were shown to readily undergo direct cyclopalladation, selectively adopting either a bi- or tridentate coordination mode depending on the nature of the bridging unit between the P=O donor group and the central amide group as well as the reaction conditions. This allowed for direct comparison of the effect of pincer vs. bidentate ligation on the anticancer potential of the resulting cyclopalladated derivatives. The results of cytotoxicity studies demonstrated that the pincer-type coordination, especially in the case when it was forced by additional steric effects, is advantageous for biological activity of the amide-based Pd(II) complexes. Furthermore, they generally confirmed the efficiency of our strategy of anchoring the labile phosphoryl site as a formal oxygen leaving group on the ligand backbone to afford a potentially tridentate pincer system.

Among the complexes obtained, the pincer-type palladocycle featuring the functionalized picolinylamide ligand with the additional phenyl substituent in the phosphoryl coordination arm exhibited prominent cytotoxic effects on several human solid and, particularly, hematopoietic cancer cell lines, including chronic myelogenous leukemia K562, multiple plasmacytoma AMO1, and acute lymphoblastic leukemia H9. The comparable levels of cytotoxic activity of most of the complexes explored against parental cell lines HBL100 and K562 and their resistant subclones HBL100/Dox and K562/iS9 opens the way to the creation of new anticancer agents that would be able to overcome drug resistance. Our further efforts will focus on developing related ligand systems with phosphine sulfide donor moieties to provide firm pincer-type coordination of Pd(II) ions and to compare the cytotoxic activity of resulting complexes with those featuring labile phosphoryl sites.

## 4. Experimental Section

### 4.1. General Remarks

If not noted otherwise, all manipulations were carried out without taking precautions to exclude air and moisture. Dichloromethane was distilled from P_2_O_5_. Triethylamine was distilled over sodium. (Aminomethyl)diphenylphosphine oxide was synthesized by the Michaelis–Arbuzov reaction between Ph_2_POEt and *N*-bromomethylphthalimide followed by the hydrolysis of the protecting group according to the published procedure [40]. [Amino(phenyl)methyl]diphenylphosphine oxide hydrochloride was obtained by treating hydrobenzamide with diphenylphosphine oxide generated in situ from Ph_2_PCl [41]. Picolinyl chloride was synthesized by the reaction of picolinic acid with SOCl_2_ in the presence of Et_3_N [42] and immediately used in a further step without purification. All other chemicals and solvents were used as purchased.

The NMR spectra were recorded on Bruker Avance 400 and Avance 500 spectrometers, and the chemical shifts (*δ*) were referenced internally by the residual (^1^H) or deuterated (^13^C) solvent signals relative to tetramethylsilane or externally to H_3_PO_4_ (^31^P) or liquid ammonia (^15^N). The ^15^N chemical shifts were extracted from the ^1^H–^15^N HMBC spectra. In all cases, the ^13^C{^1^H} NMR spectra were registered using the *J*MODECHO mode; the signals for the *C* nuclei bearing odd and even numbers of protons had opposite polarities. The NMR peak assignments for ligand **1a** and complexes **2a**, **3a**,**b** were based on the analysis of ^1^H–^1^H COSY, ^1^H–^13^C HSQC, and ^1^H–^13^C HMBC spectra. The results obtained were used to assign the NMR spectra of the other compounds obtained in this study. For the NMR spectra of the representative compounds, see Figures S1–S9 (**1a**), S11–S22 (**2a**), and S24–S35 (**3b**) in the Supporting Information. The UV–vis spectra of complexes **2a** and **3a** were registered on a Cary50 spectrometer in quartz cells with 10 mm path length (Figure S50 in the Supporting Information).

The IR spectra were recorded on a Nicolet Magna-IR750 FT spectrometer (resolution 2 cm^–1^, 128 scans). The assignment of absorption bands in the IR spectra was conducted according to [43]. For the IR spectra of the representative compounds, see Figures S10 (**1a**), S23 (**2a**), and S36 (**3b**) in the Supporting Information. Column chromatography was carried out using Macherey–Nagel silica gel 60 (MN Kieselgel 60, 70–230 mesh). Melting points were determined using an MPA 120 EZ-Melt automated melting point apparatus (Stanford Research Systems).

### 4.2. Syntheses

#### 4.2.1. *N*-[(Diphenylphosphoryl)methyl]picolinamide, **1a**


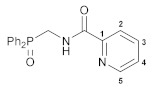


A solution of (aminomethyl)diphenylphosphine oxide (1.16 g, 5.02 mmol) and Et_3_N (0.51 g, 5.04 mmol) in dichloromethane (20 mL) was added dropwise to a solution of picolinyl chloride obtained in situ from picolinic acid (0.62 g, 5.04 mmol), SOCl_2_ (0.60 g, 5.04 mmol), and Et_3_N (0.76 g, 7.51 mmol) in CH_2_Cl_2_ (20 mL) at 0 to 5 °C. The reaction mixture was stirred at room temperature for 12 h and then washed with water. The organic layer was separated, dried over anhydrous Na_2_SO_4_, and evaporated to dryness. The resulting residue was purified by column chromatography (eluent: EtOAc) and recrystallized from EtOAc to give 0.85 g of the target compound as a white crystalline solid. Yield: 50%. Mp: 185–187 °C (EtOAc). ^31^P{^1^H} NMR (202.45 MHz, CDCl_3_): *δ* 30.18 ppm. ^1^H NMR (500.13 MHz, CDCl_3_): *δ* 4.46 (vt, 2H, CH_2_, ^2^*J*_HP_ = ^3^*J*_HH_ = 6.5 Hz), 7.41–7.43 (m, 1H, H(C4)), 7.48–7.51 (m, 4H, *m*-H in P(O)Ph_2_), 7.54–7.57 (m, 2H, *p*-H in P(O)Ph_2_), 7.81–7.86 (m, 5H, *o*-H in P(O)Ph_2_ + H(C3)), 8.11 (d, 1H, H(C2), ^3^*J*_HH_ = 7.7 Hz), 8.52 (d, 1H, H(C5), ^3^*J*_HH_ = 4.1 Hz), 8.69 (br. s, 1H, NH) ppm. ^13^C{^1^H} (125.76 MHz, CDCl_3_): *δ* 39.11 (d, CH_2_, ^1^*J*_CP_ = 78.0 Hz), 122.36 (s, C2), 126.48 (s, C4), 128.85 (d, *m*-C in P(O)Ph_2_, ^3^*J*_CP_ = 12.1 Hz), 130.60 (d, *ipso*-C in P(O)Ph_2_, ^1^*J*_CP_ = 100.0 Hz), 131.17 (d, *o*-C in P(O)Ph_2_, ^2^*J*_CP_ = 9.7 Hz), 132.42 (d, *p*-C in P(O)Ph_2_, ^4^*J*_CP_ = 2.5 Hz), 137.46 (s, C3), 148.11 (s, C5), 148.82 (s, C1), 164.19 (d, C=O, ^3^*J*_CP_ = 4.4 Hz) ppm. ^15^N NMR (50.67 MHz, CDCl_3_): *δ* 92.0 (C(O)NH), 300.6 (Py) ppm. IR (KBr, *ν*/cm^–1^): 468(w), 480(w), 504(w), 558(m), 592(w), 696(m), 718(m), 736(m), 790(w), 818(vw), 922(w), 1000(w), 1035(w), 1104(w), 1124(m), 1163(w), 1182(m) and 1193(m) (both *ν*P=O), 1241(w), 1304(vw), 1398(w), 1431(m), 1436(m), 1465(w), 1515(s) (C(O)NH), 1569(w), 1593(w), 1682(s) (*ν*C=O), 2908(w), 3025(vw), 3058(vw), 3392(m) (*ν*NH). Anal. Calcd for C_19_H_17_N_2_O_2_P: C, 67.85; H, 5.09; N, 8.33. Found: C, 67.95; H, 4.94; N, 8.41%.

#### 4.2.2. *N*-[(Diphenylphosphoryl)(phenyl)methyl]picolinamide, **1b**


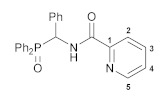


A solution of [amino(phenyl)methyl]diphenylphosphine oxide hydrochloride (1.17 g, 3.40 mmol) and Et_3_N (0.69 g, 6.82 mmol) in CH_2_Cl_2_ (20 mL) was added dropwise to a solution of picolinyl chloride obtained in situ from picolinic acid (0.42 g, 3.41 mmol), SOCl_2_ (0.41 g, 3.45 mmol), and Et_3_N (0.52 g, 5.14 mmol) in CH_2_Cl_2_ (15 mL) at 0 to 5 °C. The reaction mixture was stirred at room temperature for 12 h and then washed with water. The organic layer was separated, dried over anhydrous Na_2_SO_4_, and evaporated to dryness. The resulting residue was recrystallized from EtOAc to give 0.73 g of the target compound as a white crystalline solid. Yield: 52%. Mp: 250–252 °C (EtOAc). ^31^P{^1^H} NMR (161.98 MHz, CDCl_3_): *δ* 32.72 ppm. ^1^H NMR (400.13 MHz, CDCl_3_): *δ* 6.18 (dd, 1H, CH, ^2^*J*_HP_ = 6.7 Hz, ^3^*J*_HH_ = 10.1 Hz,), 7.21–7.25 (m, 3H, H_Ar_), 7.30–7.55 (m, 11H, H_Ar_), 7.76–7.80 (m, 1H, H(C3)), 7.96 (dd, 2H, *o*-H in P(O)Ph, ^3^*J*_HP_ = 10.5 Hz, ^3^*J*_HH_ = 7.6 Hz), 8.06 (d, 1H, H(C2), ^3^*J*_HH_ = 8.0 Hz), 8.55 (d, 1H, H(C5), ^3^*J*_HH_ = 4.4 Hz), 9.30 (dd, 1H, NH, ^3^*J*_HH_ = 10.1, ^3^*J*_HP_ = 3.2 Hz) ppm. ^13^C{^1^H} NMR (100.61 MHz, CDCl_3_–(CD_3_)_2_SO): *δ* 51.82 (d, CH, ^1^*J*_CP_ = 75.6 Hz), 121.77 (s, C2), 126.24 (s, C4), 127.59 (br. s, *p*-C in Ph), 127.94 (br. s, *m*-C in Ph), 127.95 (d, *m*-C in P(O)Ph, ^3^*J*_CP_ = 12.1 Hz), 128.28 (d, *o*-C in Ph, ^3^*J*_CP_ = 4.1 Hz), 128.34 (d, *m*-C in P(O)Ph, ^3^*J*_CP_ = 11.7 Hz), 129.80 (d, *ipso*-C in P(O)Ph, ^1^*J*_CP_ = 97.9 Hz), 130.00 (d, *ipso*-C in P(O)Ph, ^1^*J*_CP_ = 99.9 Hz), 130.91 (d, *o*-C in P(O)Ph, ^2^*J*_CP_ = 9.5 Hz), 130.96 (d, *o*-C in P(O)Ph, ^2^*J*_CP_ = 9.1 Hz), 131.69 (d, *p*-C in P(O)Ph, ^4^*J*_CP_ = 2.8 Hz), 131.94 (d, *p*-C in P(O)Ph, ^4^*J*_CP_ = 2.2 Hz), 134.35 (s, *ipso*-C in Ph), 136.92 (s, C3), 147.97 (s, C5), 148.41 (s, C1), 163.21 (d, C=O, ^3^*J*_CP_ = 6.3 Hz) ppm. IR (KBr, *ν*/cm^–1^): 509(m), 548(s), 623(w), 647(w), 701(s), 724(m), 753(m), 783(vw), 822(vw), 998(w), 1041(vw), 1103(sh, m), 1119(m), 1154(w), 1197(s) (*ν*P=O), 1240(vw), 1291(w), 1352(w), 1437(s), 1467(m), 1513(br, s) (C(O)NH), 1570(w), 1591(w), 1672(s) (*ν*C=O), 2951(vw), 3056(w), 3363(m) (*ν*NH). Anal. Calcd for C_25_H_21_N_2_O_2_P: C, 72.81; H, 5.13; N, 6.79. Found: C, 72.75; H, 5.10; N, 6.69%.

#### 4.2.3. Complex [*κ*^2^-*N*,*N*-(L)Pd(II)Cl] **2a**


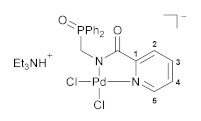


A solution of PdCl_2_(NCPh)_2_ (67 mg, 0.175 mmol) in CH_2_Cl_2_ (3 mL) was added dropwise to a solution of ligand **1a** (59 mg, 0.175 mmol) and Et_3_N (25 μL, 0.179 mmol) in CH_2_Cl_2_ (5 mL). The reaction mixture was left under ambient conditions for 12 h and then evaporated to dryness. The resulting residue was washed with Et_2_O and dried under vacuum to give 97 mg of complex **2a** as a yellow crystalline solid. Yield: 90%. Mp: >175 °C (dec.). ^31^P{^1^H} NMR (202.45 MHz, CDCl_3_): *δ* 34.17 ppm. ^1^H NMR (500.13 MHz, CDCl_3_): *δ* 1.40 (t, 9H, Me, ^3^*J*_HH_ = 7.3 Hz), 3.29–3.35 (m, 6H, CH_2_ in Et_3_NH^+^), 4.68 (d, 2H, CH_2_, ^2^*J*_HP_ = 6.3 Hz), 7.31–7.34 (m, 1H, H(C4)), 7.43–7.51 (m, 6H, *m*-H and *p*-H in P(O)Ph_2_), 7.64 (d, 1H, H(C2), ^3^*J*_HH_ = 7.6 Hz), 7.82–7.85 (m, 1H, H(C3)), 7.94 (dd, 4H, *o*-H in P(O)Ph_2_, *^3^J*_HP_ = 10.8 Hz, ^3^*J*_HH_ = 7.8 Hz,), 9.12 (d, 1H, H(C5), ^3^*J*_HH_ = 5.2 Hz), 10.95 (br. s, 1H, NH in Et_3_NH^+^) ppm. ^13^C{^1^H} NMR (125.76 MHz, CDCl_3_): *δ* 8.78 (s, Me), 46.02 (s, CH_2_ in Et_3_NH^+^), 46.20 (d, CH_2_, ^1^*J*_CP_ = 73.8 Hz), 124.74 (s, C2), 125.94 (s, C4), 128.34 (d, *m*-C in P(O)Ph_2_, ^3^*J*_CP_ = 11.8 Hz), 131.63 (d, *o*-C in P(O)Ph_2_, ^2^*J*_CP_ = 9.5 Hz), 131.88 (d, *p*-C in P(O)Ph_2_, ^4^*J*_CP_ = 1.5 Hz), 131.91 (d, *ipso*-C in P(O)Ph_2_, ^1^*J*_CP_ = 97.4 Hz), 138.97 (s, C3), 148.44 (s, C5), 154.31 (s, C1), 171.41 (d, C=O, ^3^*J*_CP_ = 2.7 Hz) ppm. ^15^N NMR (50.67 MHz, CDCl_3_): *δ* 56.4 (Et_3_NH^+^), 118.1 (C(O)N), 212.8 (Py) ppm. IR (KBr, *ν*/cm^–1^): 483(w), 504(w), 522(m), 566(w), 701(m), 712(w), 746(m), 758(w), 804(w), 939(w), 1047(w), 1071(w), 1103(w), 1119(w), 1169(s) (*ν*P=O), 1292(vw), 1381(m), 1394(m), 1438(m), 1476(w), 1570(w), 1599(s), 1625(s) (*ν*C=O), 2520(br, vw), 2684(br, w), and 2788(br, vw) (three *ν*NH in Et_3_NH^+^), 2958(w), 2977(w), 3055(vw). Anal. Calcd for C_25_H_32_Cl_2_N_3_O_2_PPd: C, 48.84; H, 5.25; N, 6.83. Found: C, 48.85; H, 5.29; N, 6.68%.

#### 4.2.4. Complex [*κ*^2^-*N*,*N*-(L)Pd(II)Cl] **2b**


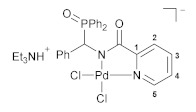


A solution of PdCl_2_(NCPh)_2_ (81 mg, 0.211 mmol) in CH_2_Cl_2_ (5 mL) was added dropwise to a solution of ligand **1a** (0.087 mg, 0.211 mmol) and Et_3_N (30 μL, 0.215 mmol) in CH_2_Cl_2_ (5 mL). The reaction mixture was left under ambient conditions for 12 h and then half evaporated. The addition of Et_2_O (10 mL) afforded a yellow precipitate, which was collected by filtration, dried in air, and then recrystallized from CH_2_Cl_2_–Et_2_O to give 121 mg of complex **2b** as a yellow crystalline solid. Yield: 78%. Mp: >155 °C (dec.). IR (KBr, *ν*/cm^–1^): 498(w), 518(w), 544(m), 702(m), 721(w), 736(w), 758(w), 810(vw), 837(vw), 942(vw), 1033(w), 1072(w), 1114(w), 1170(br, m) (*ν*P=O), 1290(w), 1399(m), 1437(m), 1451(w), 1471(w), 1494(w), 1574(vw), 1599(m), 1630(s) (*ν*C=O), 2500(br, vw) and 2680(br, w) (both *ν*NH in Et_3_NH^+^), 2913(vw), 2996(w), 3055(w). Anal. Calcd for C_31_H_36_Cl_2_N_3_O_2_PPd·0.5CH_2_Cl_2_: C, 51.59; H, 5.09; N, 5.73. Found: C, 51.35; H, 4.99; N, 5.76%.

#### 4.2.5. Complex [*κ*^3^-*O*,*N*,*N*-(L)Pd(II)Cl] **3a**


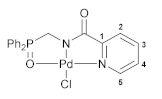


AgBF_4_ (37 mg, 0.191 mmol) was added to a solution of complex **2a** (117 mg, 0.190 mmol) in CH_2_Cl_2_ (20 mL). The reaction mixture was stirred at room temperature for 12 h and then filtered through a pad of cotton. The filtrate was evaporated to dryness. The resulting residue was purified by column chromatography on silica gel (eluent: CH_2_Cl_2_–EtOH (25:1)) to give 50 mg of the target pincer complex as a yellow crystalline solid. Yield: 55%. Mp: >155 °C (dec.). ^31^P{^1^H} NMR (161.98 MHz, CDCl_3_): *δ* 73.39 ppm. ^1^H NMR (400.13 MHz, CDCl_3_): *δ* 4.38 (d, 2H, CH_2_, ^2^*J*_HP_ = 2.0 Hz), 7.39–7.42 (m, 1H, H(C4)), 7.57–7.62 (m, 4H, *m*-H in P(O)Ph_2_), 7.68–7.71 (m, 2H, *p*-H in P(O)Ph_2_), 7.75 (d, 1H, H(C2), ^3^*J*_HH_ = 7.6 Hz), 7.89 (dd, 4H, *o*-H in P(O)Ph_2_, ^3^*J*_HP_ = 11.9 Hz, ^3^*J*_HH_ = 7.8 Hz), 7.94–7.98 (m, 1H, H(C3)), 8.87 (d, 1H, H(C5), ^3^*J*_HH_ = 5.0 Hz) ppm. ^13^C{^1^H} NMR (125.76 MHz, CDCl_3_): *δ* 48.46 (d, CH_2_, ^1^*J*_CP_ = 86.0 Hz), 125.51 (s, C2), 126.86 (d, *ipso*-C in P(O)Ph_2_, ^1^*J*_CP_ = 101.3 Hz), 126.95 (s, C4), 129.42 (d, *m*-C in P(O)Ph_2_, ^3^*J*_CP_ = 12.6 Hz), 131.60 (d, *o*-C in P(O)Ph_2_, ^2^*J*_CP_ = 10.2 Hz), 134.04 (br. s, *p*-C in P(O)Ph_2_), 139.89 (s, C3), 150.74 (s, C5), 155.91 (s, C1), 169.80 (d, C=O, ^3^*J*_CP_ = 12.1 Hz) ppm. ^15^N NMR (50.67 MHz, CDCl_3_): *δ* 114.4 (C(O)N), 197.4 (Py) ppm. IR (KBr, *ν*/cm^–1^): 479(w), 500(w), 524(m), 572(m), 676(w), 697(m), 723(m), 741(m), 750(m), 813(w), 937(vw), 997(vw), 1025(vw), 1044(w), 1057(w), 1073(w), 1087(m), 1100(w), 1123(m) and 1135(m) (both *ν*P=O), 1185(vw), 1251(vw), 1296(w), 1385(m), 1437(m), 1483(vw), 1569(w), 1601(s), 1627(s) (*ν*C=O), 2871(w), 2926(w), 2972(vw), 3054(w). Anal. Calcd for C_19_H_16_ClN_2_O_2_PPd: C, 47.82; H, 3.38; N, 5.87. Found: C, 47.61; H, 3.88; N, 5.44%.

#### 4.2.6. Complex [*κ*^3^-*O*,*N*,*N*-(L)Pd(II)Cl] **3b**


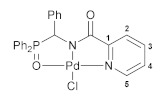


AgBF_4_ (23 mg, 0.119 mmol) was added to a solution of complex **2b** (87 mg, 0.119 mmol) in CH_2_Cl_2_ (15 mL). The reaction mixture was stirred at room temperature for 12 h and then filtered through a pad of cotton. The filtrate was evaporated to dryness. The resulting residue was washed with EtOH and dried under vacuum to give 46 mg of the target pincer complex as a yellow crystalline solid. Yield: 70%. Mp: >210 °C (dec.). ^31^P{^1^H} NMR (202.45 MHz, CDCl_3_): *δ* 73.89 ppm. ^1^H NMR (500.13 MHz, CDCl_3_): *δ* 5.66 (br. s, 1H, CH), 7.17–7.30 (m, 7H, *o*-H and *m*-H in P(O)Ph + *m*-H and *p*-H in Ph), 7.41–7.45 (m, 2H, *p*-H in P(O)Ph + H(C4)), 7.65–7.68 (m, 2H, *o*-H in Ph), 7.69–7.74 (m, 3H, *m*-H in P(O)Ph + H(C2)), 7.75–7.79 (m, 1H, *p*-H in P(O)Ph), 7.95 (dt, 1H, H(C3), ^3^*J*_HH_ = 7.7 Hz, ^4^*J*_HH_ = 1.5 Hz), 8.14–8.18 (m, 2H, *o*-H in P(O)Ph), 8.93 (dd, 1H, H(C5), ^3^*J*_HH_ = 5.6 Hz, ^4^*J*_HH_ = 1.3 Hz) ppm. ^13^C{^1^H} NMR (125.76 MHz, CDCl_3_): *δ* 62.21 (d, CH, ^1^*J*_CP_ = 81.8 Hz), 125.70 (d, *ipso*-C in P(O)Ph, ^1^*J*_CP_ = 102.7 Hz), 125.73 (s, C2), 127.08 (s, C4), 127.32 (d, *ipso*-C in P(O)Ph, ^1^*J*_CP_ = 94.2 Hz), 127.74 (d, *o*-C in Ph, ^3^*J*_CP_ = 5.5 Hz), 128.34 (d, *m*-C in P(O)Ph, ^3^*J*_CP_ = 12.7 Hz), 128.37 (s, *p*-C in Ph), 128.57 (d, *m*-C in Ph, ^4^*J*_CP_ = 2.4 Hz), 129.69 (d, *m*-C in P(O)Ph, ^3^*J*_CP_ = 12.7 Hz), 131.97 (d, *o*-C in P(O)Ph, ^2^*J*_CP_ = 9.1 Hz), 132.00 (d, *o*-C in P(O)Ph, ^2^*J*_CP_ = 10.0 Hz), 133.43 (d, *p*-C in P(O)Ph, ^4^*J*_CP_ = 2.7 Hz), 133.90 (d, *p*-C in P(O)Ph, ^4^*J*_CP_ = 2.6 Hz), 134.56 (d, *ipso*-C in Ph, ^2^*J*_CP_ = 2.8 Hz), 139.84 (c, C3), 150.64 (s, C5), 155.88 (s, C1), 169.32 (d, C=O, ^3^*J*_CP_ = 13.4 Hz) ppm. ^15^N NMR (50.67 MHz, CDCl_3_): *δ* 128.9 (C(O)N), 198.6 (Py) ppm. IR (KBr, *ν*/cm^–1^): 495(w), 525(m), 556(s), 581(w), 695(m), 708(w), 729(w), 767(w), 785(w), 810(vw), 999(w), 1022(m), 1038(w), 1120(br, m) (*ν*P=O), 1158(vw), 1191(vw), 1289(w), 1374(br, m), 1437(m), 1452(w), 1467(vw), 1492(w), 1601(m), 1636(s) (*ν*C=O), 2922(w), 2954(vw), 3060(w). Anal. Calcd for C_25_H_20_ClN_2_O_2_PPd: C, 54.27; H, 3.64; N, 5.06. Found: C, 53.73; H, 3.90; N, 4.88%.

#### 4.2.7. *N*-[(Diphenylphosphoryl)(phenyl)methyl]quinoline-2-carboxamide, **4**


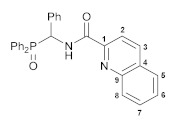


A mixture of quinoline-2-carboxylic acid (0.17 g, 0.98 mmol) and SOCl_2_ (4 mL) was refluxed for 12 h. After cooling to room temperature, the excess of SOCl_2_ was removed under vacuum to give quinoline-2-carbonyl chloride. A solution of the latter in CH_2_Cl_2_ (10 mL) was added to a solution of [amino(phenyl)methyl]diphenylphosphine oxide hydrochloride (0.34 g, 0.99 mmol) and Et_3_N (0.50 g, 0.49 mmol) in CH_2_Cl_2_ (20 mL) at 5–10 °C. The resulting mixture was stirred at room temperature for 8 h and then washed with water. The organic layer was separated, dried over anhydrous Na_2_SO_4_, and evaporated to dryness. The residue obtained was purified by column chromatography on silica gel (eluent: CHCl_3_–EtOAc (10:1)) to give 0.25 g of ligand **4** as a white crystalline solid. Yield: 55%. Mp: 192–194 °C. ^31^P{^1^H} NMR (161.98 MHz, CDCl_3_): *δ* 32.76 ppm. ^1^H NMR (400.13 MHz, CDCl_3_): *δ* 6.27 (dd, 1H, CH, ^2^*J*_HP_ = 7.2 Hz, ^3^*J*_HH_ = 10.4 Hz), 7.23–7.27 (m, 3H, H_Ar_), 7.33–7.37 (m, 2H, *m*-H in P(O)Ph), 7.41–7.65 (m, 9H, H_Ar_), 7.78–7.82 (m, 1H, H_Ar_), 7.86 (d, 1H, H_Ar_, ^3^*J*_HH_ = 8.1 Hz), 7.96–8.02 (m, 2H, *o*-H in P(O)Ph), 8.18 (d, 1H, H_Ar_, ^3^*J*_HH_ = 8.4 Hz), 8.22 (d, 1H, H_Ar_, ^3^*J*_HH_ = 8.4 Hz), 8.27 (d, 1H, H_Ar_, ^3^*J*_HH_ = 8.6 Hz), 9.47 (dd, 1H, NH, ^3^*J*_HH_ = 10.4 Hz, ^3^*J*_HP_ = 3.2 Hz) ppm. ^13^C{^1^H} NMR (125.76 MHz, CDCl_3_): *δ* 52.45 (d, CH, ^1^*J*_CP_ = 75.8 Hz), 118.80 (s, C2), 127.62 (s, C6 or C5), 128.05 (s, *p*-C in Ph), 128.18 (s, C5 or C6), 128.37 (d, *m*-C in P(O)Ph, ^3^*J*_CP_ = 11.4 Hz), 128.41 (s, *m*-C in Ph), 128.73 (d, *m*-C in P(O)Ph, ^3^*J*_CP_ = 11.8 Hz), 128.87 (d, *o*-C in Ph, ^3^*J*_CP_ = 4.1 Hz), 129.41 (s, C4), 130.06 and 130.23 (both s, C7 and C8), 130.24 (d, *ipso*-C in P(O)Ph, ^1^*J*_CP_ = 99.4 Hz), 130.64 (d, *ipso*-C in P(O)Ph, ^1^*J*_CP_ = 100.5 Hz), 131.44 (d, *o*-C in P(O)Ph, ^2^*J*_CP_ = 9.2 Hz), 131.57 (d, *o*-C in P(O)Ph, ^2^*J*_CP_ = 8.8 Hz), 132.05 (br. s, *p*-C in P(O)Ph), 132.31 (br. s, *p*-C in P(O)Ph), 134.70 (s, *ipso*-C in Ph), 137.48 (s, C3), 146.45 and 148.72 (both s, C1 and C9), 163.89 (d, C=O, ^3^*J*_CP_ = 5.9 Hz) ppm. IR (KBr, *ν*/cm^–1^): 492(w), 512(w), 528(w), 549(s), 623(w), 698(s), 723(m), 759(m), 775(m), 837(w), 915(vw), 998(vw), 1074(w), 1120(m), 1164(m), 1180(br, m) (*ν*P=O), 1211(w), 1247(w), 1295(w), 1340(w), 1427(w), 1438(m), 1456(w), 1498(m), 1519(m), 1539(m) (C(O)H), 1594(w), 1618(w), 1666(s) (*ν*C=O), 2935(vw), 3058(w), 3237(br, w) and 3371(br, w) (both *ν*NH). Anal. Calcd for C_29_H_23_N_2_O_2_P: C, 75.31; H, 5.01; N, 6.06. Found: C, 75.42; H, 5.11; N, 6.17%.

#### 4.2.8. Complex [*κ*^3^-*O*,*N*,*N*-(L)Pd(II)Cl] **5**


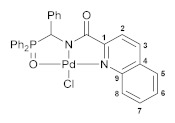


A solution of PdCl_2_(NCPh)_2_ (70 mg, 0.182 mmol) in CH_2_Cl_2_ (4 mL) was added dropwise to a solution of ligand **4** (84 mg, 0.182 mmol) and Et_3_N (26 μL, 0.186 mmol) in CH_2_Cl_2_ (6 mL). The reaction mixture was left under ambient conditions for 1 day and then filtered through a pad of cotton. The filtrate was evaporated to dryness. The resulting residue was washed with Et_2_O and purified by column chromatography on silica gel (eluent: CHCl_3_–EtOH (25:1)) to give 60 mg of the target pincer complex as a yellow crystalline solid. Yield: 55%. Mp: >175 °C (dec.). ^31^P{^1^H} NMR (161.98 MHz, CH_2_Cl_2_/D_2_O): *δ* 72.32 ppm (the ^1^H and ^13^C{^1^H} NMR spectroscopic data for complex **5** were not obtained due to its low solubility in common organic solvents (e.g., chlorinated hydrocarbons and acetonitrile) and instability in strongly coordinating media (e.g., DMSO)). IR (KBr, *ν*/cm^–1^): 521(w), 534(w), 561(s), 586(w), 699(m), 725(w), 766(w), 852(w), 927(vw), 998(w), 1024(m), 1042(m), 1121(m) (*ν*P=O), 1154(w), 1341(w), 1378(br, m), 1437(m), 1461(w), 1493(w), 1516(w), 1560(w), 1595(w), 1633(s) (*ν*C=O), 2923(vw), 3064(w). Anal. Calcd for C_29_H_22_ClN_2_O_2_PPd: C, 57.73; H, 3.68; N, 4.64. Found: C, 57.45; H, 3.73; N, 4.54%.

#### 4.2.9. *N*-[(Diphenylphosphoryl)methyl]quinoline-8-carboxamide, **6**


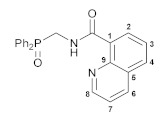


A solution of thionyl chloride (0.24 g, 2.02 mmol) in CH_2_Cl_2_ (5 mL) was added to a solution of quinoline-8-carboxylic acid (0.35 g, 2.02 mmol) and Et_3_N (0.21 g, 2.08 mmol) in CH_2_Cl_2_ (10 mL) at 5 °C. The resulting mixture was stirred at room temperature for 1 h. Then a solution of (aminomethyl)diphenylphosphine oxide (0.46 g, 1.99 mmol) and Et_3_N (0.21 g, 2.08 mmol) in CH_2_Cl_2_ (15 mL) was added. The reaction mixture was stirred at room temperature for 8 h and washed with water. The organic layer was separated, dried over anhydrous Na_2_SO_4_, and evaporated to dryness. The residue obtained was purified by column chromatography on silica gel (eluent: EtOAc–EtOH (10:1)) to give 0.43 g of ligand **6** as a white crystalline solid. Yield: 56%. Mp: 190–192 °C. ^31^P{^1^H} NMR (161.98 MHz, CDCl_3_): *δ* 30.58 ppm. ^1^H NMR (400.13 MHz, CDCl_3_): *δ* 4.66–4.69 (m, 2H, CH_2_), 7.44–7.57 (m, 7H, H_Ar_), 7.63–7.67 (m, 1H, H(C3)), 7.88–7.96 (m, 5H, H_Ar_), 8.25 (d, 1H, H_Ar_, ^3^*J*_HH_ = 8.2 Hz), 8.74–8.76 (m, 1H, H(C8)), 8.79 (d, 1H, H_Ar_, ^3^*J*_HH_ = 7.4 Hz), 11.99 (br. s, 1H, NH) ppm. ^13^C{^1^H} NMR (100.61 MHz, CDCl_3_): *δ* 39.79 (d, CH_2_, ^1^*J*_CP_ = 79.1 Hz), 121.00 (s, C7), 126.46 (s, C3), 127.95 and 128.42 (both s, C1 and C5), 128.73 (d, *m*-C in P(O)Ph_2_, ^3^*J*_CP_ = 11.7 Hz), 131.18 (d, *ipso*-C in P(O)Ph_2_, ^1^*J*_CP_ = 99.6 Hz), 131.38 (d, *o*-C in P(O)Ph_2_, ^2^*J*_CP_ = 9.7 Hz), 132.19–132.22 (overlapping signals of *p*-C in P(O)Ph_2_ and C2 or C4), 133.97 (s, C4 or C2), 137,74 (s, C6), 145.32 (s, C9), 149.40 (s, C8), 166.01 (d, C=O, ^3^*J*_CP_ = 5.8 Hz) ppm. IR (KBr, *ν*/cm^–1^): 500(m), 515(w), 537(m), 547(m), 579(vw), 644(w), 693(w), 702(w), 723(m), 731(m), 752(w), 765(w), 800(m), 841(w), 916(w), 997(vw), 1052(vw), 1072(vw), 1124(m), 1189(s) (*ν*P=O), 1236(vw), 1275(vw), 1294(w), 1382(w), 1405(w), 1439(m), 1462(vw), 1501(w), 1557(br, s) (C(O)NH), 1575(m), 1592(m), 1612(w), 1647(s) (*ν*C=O), 2913(w), 2993(w), 3058(w), 3127(br, w) (*ν*NH). Anal. Calcd for C_23_H_19_N_2_O_2_P: C, 71.50; H, 4.96; N, 7.25. Found: C, 71.41; H, 5.03; N, 7.20%.

#### 4.2.10. Complex [*κ*^3^-*O*,*N*,*N*-(L)Pd(II)Cl] **7**


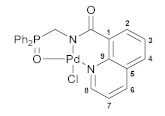


A solution of PdCl_2_(NCPh)_2_ (64 mg, 0.167 mmol) in CH_2_Cl_2_ (4 mL) was added dropwise to a solution of ligand **6** (65 mg, 0.168 mmol) and Et_3_N (24 μL, 0.172 mmol) in CH_2_Cl_2_ (7 mL). The reaction mixture was left under ambient conditions for 1 day and then filtered through a pad of cotton. The filtrate was evaporated to dryness. The resulting residue was washed with Et_2_O and purified by column chromatography on silica gel (eluent: CHCl_3_–EtOH (25:1)) to give 53 mg of the target pincer complex as a yellow crystalline solid. Yield: 60%. Mp: >130 °C (dec.). ^31^P{^1^H} NMR (161.98 MHz, CDCl_3_): *δ* 66.49 ppm. ^1^H NMR (400.13 MHz, CDCl_3_): *δ* 4.83 (d, 2H, CH_2_, ^2^*J*_HP_ = 4.1 Hz), 7.35 (dd, 1H, H(C7), ^3^*J*_HH_ = 8.1 Hz, ^3^*J*_HH_ = 5.6 Hz), 7.55–7.59 (m, 4H, *m*-H in P(O)Ph_2_), 7.63–7.69 (m, 3H, *p*-H in P(O)Ph_2_ + H_Ar_), 7.87–7.92 (m, 5H, *o*-H in P(O)Ph_2_ + H_Ar_), 8.35 (dd, 1H, H_Ar_, ^3^*J*_HH_ = 8.0 Hz, ^4^*J*_HH_ = 1.1 Hz), 8.94 (dd, 1H, H_Ar_, ^3^*J*_HH_ = 7.5 Hz, ^4^*J*_HH_ = 1.2 Hz), 9.70 (dd, 1H, H(C8), ^3^*J*_HH_ = 5.6 Hz, ^4^*J*_HH_ = 1.2 Hz) ppm. ^13^C{^1^H} NMR (100.61 MHz, CDCl_3_): *δ* 51.65 (d, CH_2_, ^1^*J*_CP_ = 82.7 Hz), 120.93 (s, C7), 126.00 (d, *ipso*-C in P(O)Ph_2_, ^1^*J*_CP_ = 100.3 Hz), 127.53 (s, C3), 129.25 (d, *m*-C in P(O)Ph_2_, ^3^*J*_CP_ = 12.5 Hz), 129.26 and 131.10 (both s, C1 and C5), 131.84 (s, C2 or C4), 131.96 (d, *o*-C in P(O)Ph_2_, ^2^*J*_CP_ = 11.0 Hz), 133.83 (d, *p*-C in P(O)Ph_2_, ^4^*J*_CP_ = 2.5 Hz), 137.01 (s, C4 or C2), 140.94 (s, C6), 143.14 (s, C9), 158.32 (s, C8), 162.63 (d, C=O, ^3^*J*_CP_ = 7.2 Hz) ppm. IR (KBr, *ν*/cm^–1^): 488(w), 562(m), 592(w), 619(vw), 691(w), 729(w), 745(w), 782(w), 837(w), 859(vw), 926(w), 997(w), 1026(w), 1048(m), 1092(w), 1125(br, m) (*ν*P=O), 1153(w), 1176(w), 1305(w), 1369(br, m), 1438(m), 1486(vw), 1509(w), 1561(s), 1582(m), 1615(m) (*ν*C=O), 2885(vw), 2962(vw), 3055(w). Anal. Calcd for C_23_H_18_ClN_2_O_2_PPd: C, 52.39; H, 3.44; N, 5.31. Found: C, 52.26; H, 3.52; N, 5.31%.

### 4.3. X-ray Crystallography

Single crystals of the compounds explored were obtained by slow crystallization from MeCN (**1b**), CH_2_Cl_2_–Et_2_O (**2a**, **5**), and CHCl_3_–Et_2_O (**2b**, **3b**, **7**). X-ray diffraction data were collected at 120 K with a Bruker ApexII DUO CCD diffractometer using graphite-monochromated Mo-Kα radiation (*λ* = 0.71073 Å). Using Olex2 [44], the structures were solved with the ShelXT structure solution program [45] using Intrinsic Phasing and refined with the XL refinement package [46] using Least-Squares minimization against F^2^_hkl_ in anisotropic approximation for non-hydrogen atoms. The positions of NH hydrogen atoms in compounds **1b**, **2a**, and **2b** were found from the difference Fourier synthesis, while the positions of other hydrogen atoms were calculated; all were refined in the isotropic approximation within the riding model. Crystal data and structure refinement parameters are given in Table S2 in the SI. CCDC 2242547, 2242548, 2242549, 2242550, 2242552, and 2242554 contain supplementary crystallographic data for **1b**, **2a**, **2b**, **3b**, **5** and **7**, respectively. These data can be obtained free of charge via www.ccdc.cam.ac.uk/15.3.2023/cif, or by emailing data_request@ccdc.cam.ac.uk, or by contacting The Cambridge Crystallographic Data Centre, 12 Union Road, Cambridge CB2 1EZ, UK; fax: +44 1223 336033.

### 4.4. Cytotoxicity Studies

The cytotoxic activity of the compounds explored was investigated on human colorectal carcinoma (HCT116), breast cancer (MCF7), prostate adenocarcinoma (PC3), glioblastoma (U251), ovarian adenocarcinoma (Scov3), chronic myelogenous leukemia (K562 and K562/iS9), multiple plasmacytoma (AMO1), and acute lymphoblastic leukemia (H9) cell lines, as well as human embryonic kidney (HEK293) and mammary epithelial (HBL100 and HBL100/Dox) cells used as representatives of pseudonormal cells. All cell lines were obtained from American Type Culture Collection (ATCC). RPMI-1640 and DMEM media were obtained from Gibco. Fetal bovine serum (FBS) was purchased from HyClone. Cells were cultured in RPMI-1640 or DMEM media supplemented with 10% FBS and 50 μg/mL gentamicin in a humidified incubator with 5% CO_2_ atmosphere. The cell growth inhibitory effects of the compounds were evaluated using the conventional MTT assay (ICN Biomedicals, Eschwege, Germany). Cells were seeded in triplicate at a cell density of 5 × 10^3^/well in 96-well plates in 100 µL complete medium and preincubated for 24 h. The tested compounds were initially dissolved in DMSO. Then, the compounds at various concentrations were added to the media. The well plates were incubated for 48 h followed by addition of MTT solution (Sigma, Darmstadt, Germany) (20 μL, 5 mg/mL). The cells were incubated at 37 °C for further 3 h; then the culture medium was removed, and formazan crystals were dissolved in DMSO (70 μL). The absorbance of the resulting solutions was measured on a multi-well plate reader (Multiskan FC, Thermo scientific) at 530 nm to determine the percentage of surviving cells. The reported values of IC_50_ are the averages of three independent experiments (Table 2 and Table 3). Cisplatin (in the initial form of an infusion concentrate in natural saline solution) from a commercial source was used as the reference.

### 4.5. Apoptosis Induction Assay

To study the apoptosis inducing ability of complex **3b**, K562 and K562/iS9 cells, preincubated for a day in a CO_2_ incubator at 37 °C, were cultured in the medium containing 10 μM of the palladocycle for 20 h. After exposure, the cells were washed with cold PBS and incubated with Annexin V-FITC for 20 min before being treated with PI according to the supplier protocol (Elabscience Annexin V-FITC/PI Apoptosis Detection Kit). The apoptotic rates of the resulting cell samples were analyzed on a FACScan flow cytometer (Becton Dickinson Franklin Lakes NJ USA) using the CellQuest software (version 3.3).

## Figures and Tables

**Table 1:** Selected bond lengths (Å) and angles (°) for compounds **1b**, **2a**,**b**, and **3b**.

	1b	2a	2b	3b
Pd1–Cl1	–	2.3031(7)	2.305(2)	2.3137(8)
Pd1–X ^1^	–	2.2977(7)	2.309(2)	2.060(2)
Pd1–N1	–	2.023(2)	2.037(6)	2.000(3)
Pd1–N2	–	2.0084(18)	2.025(6)	1.973(3)
P1–O2	1.4843(11)	1.4934(17)	1.502(5)	1.536(2)
N1–C1	1.3438(18)	1.349(3)	1.341(9)	1.367(4)
C1–C6	1.4997(19)	1.494(3)	1.509(10)	1.519(5)
C6–O1	1.2295(17)	1.245(3)	1.242(8)	1.241(4)
C6–N2	1.3373(18)	1.332(3)	1.336(9)	1.329(5)
Cl1–Pd1–N2	–	174.56(6)	174.66(18)	173.19(8)
X–Pd1–N1 ^1^	–	175.37(6)	174.45(17)	167.56(10)
N2–Pd1–N1	–	80.43(8)	80.6(2)	80.99(11)
N1–Pd1–Cl1	–	94.48(6)	94.45(17)	98.66(8)
Cl1–Pd1–X ^1^	–	89.83(3)	90.52(8)	93.78(6)
X–Pd1–N2 ^1^	–	95.33(6)	94.53(17)	86.73(10)

^1^ X = Cl2 (**2a**,**b**), O2 (**3b**).

**Table 2:** Cytotoxicity of the phosphoryl-functionalized amide derivatives against some human solid cancer and non-cancerous cell lines.

Entry	Comp.	IC_50_ ± SD ^1^, μM							
		HCT116	MCF7	PC3	U251	Scov3	HEK293	HBL100	HBL100/Dox
1	**1a**	>100.0 ^2^	46% ^3^	>100.0 ^2^	>100.0 ^2^	>100.0 ^2^	>100.0 ^2^	>100.0 ^2^	35% ^3^
2	**2a**	30.0 ± 5.0	33.0 ± 7.0	22.0 ± 10.0	>80.0 ^4^	>80.0 ^4^	24.0 ± 6.0	>80.0 ^4^	>80.0 ^4^
3	**2b**	3.0 ± 0.5	11.0 ± 2.5	9.0 ± 1.5	43.0 ± 1.5	36.0 ± 2.5	6.8 ± 0.2	30.0 ± 3.5	26.0 ± 7.2
4	**3a**	36.0 ± 2.0	45.0 ± 5.0	26.0 ± 6.0	>80.0 ^4^	30% ^5^	34.0 ± 3.0	>80.0 ^4^	>80.0 ^4^
5	**3b**	4.0 ± 2.0	13.0 ± 1.5	16.0 ± 2.0	43.0 ± 5.0	40.0 ± 0.6	12.5 ± 3.5	17.8 ± 0.8	28.0 ± 2.6
6	**7**	38.0 ± 6.0	58.0 ± 12.0	52.0 ± 10.0	n/d	n/d	23.0 ± 3.0	n/d	n/d
7	**Cisplatin**	18.0 ± 2.0	25.0 ± 4.0	16.0 ± 3.0	16.5 ± 1.5	21.0 ± 3.0	12.5 ± 1.5	14.6 ± 3.6	23.6 ± 3.6

^1^ SD is the standard deviation of the value; ^2^ over 50% of live cells at the compound concentration of 100.0 μM; ^3^ the percentage of live cells at the compound concentration of 100.0 μM; ^4^ over 50% of live cells at the compound concentration of 80.0 μM; ^5^ the percentage of live cells at the compound concentration of 80.0 μM; n/d not defined.

**Table 3:** Cytotoxicity of the phosphoryl-functionalized amide derivatives against some human hematopoietic *c*ancer cell lines.

Entry	Comp.	IC_50_ ± SD ^1^, μM			
		K562	K562/iS9	AMO1	H9
1	**1a**	>80.0 ^2^	>80.0 ^2^	41% ^3^	>80.0 ^2^
2	**2a**	36.0 ± 2.0	44.0 ± 2.0	40.0 ± 2.0	32.0 ± 1.0
3	**2b**	11.0 ± 3.4	8.5 ± 0.5	16.0 ± 1.0	12.5 ± 1.5
4	**3a**	36.0 ± 4.0	40.0 ± 4.5	32.0 ± 2.0	23.0 ± 1.0
5	**3b**	6.4 ± 0.4	7.2 ± 1.0	2.7 ± 0.5	3.2 ± 0.2
6	**Cisplatin**	15.5 ± 0.5	16.0 ± 2.0	3.2 ± 0.6	3.0 ± 1.0

^1^ SD is the standard deviation of the value; ^2^ over 50% of live cells at the compound concentration of 80.0 μM; ^3^ the percentage of live cells at the compound concentration of 80.0 μM.

## Data Availability

The data presented in this study are available in the article and supporting information.

## Supplementary Materials

The following supporting information can be downloaded at: https://www.mdpi.com/article/10.3390/pharmaceutics15041088/s1, Figures S1–S46: NMR and IR spectra of ligand **1a** and complexes **2a**,**b**, **3b**; Figure S47: fragments of the crystal packing of complexes **2a**,**b** and **3b**; Figures S48, S49: ^31^P NMR spectra of complexes **2b** and **3b** in CDCl_3_–(CD_3_)_2_SO mixture (stability studies); Figure S50: UV–vis spectra of complexes **2a** and **3a** in different media (stability studies); Figure S51: effect of complexes **2b** and **3b** preliminarily kept in neat DMSO and DMSO–RPMI 1640 mixture on AMO1 and K562 cells; Table S1: selected bond lengths and angles for complexes **5** and **7**; Table S2: crystal data and structure refinement parameters for compounds **1b**, **2a**,**b**, **3b**, **5**, and **7**.
