# Supplementary material for: Modulation of the Cytotoxic Properties of Pd(II) Complexes Based on Functionalized Carboxamides Featuring Labile Phosphoryl Coordination Sites
Source: Pharmaceutics. 2023 Mar 28;15(4):1088. doi: 10.3390/pharmaceutics15041088 (PMC10146186; doi:10.3390/pharmaceutics15041088)
Supplement: Supplementary file 1 [file pharmaceutics-15-01088-s001.zip › pharmaceutics-2280759-supplementary.pdf]

Modulation of the Cytotoxic Properties of Pd(II) Complexes Based on Functionalized Carboxamides Featuring Labile Phosphoryl Coordination Sites

Diana V. Aleksanyan, Aleksandr V. Konovalov, Svetlana G. Churusova, Ekaterina Yu. Rybalkin, Alexander S. Peregudov, Svetlana A. Aksenova, Evgenii I. Gutsul, Zinaida S. Klemenkova and Vladimir A. Kozlov

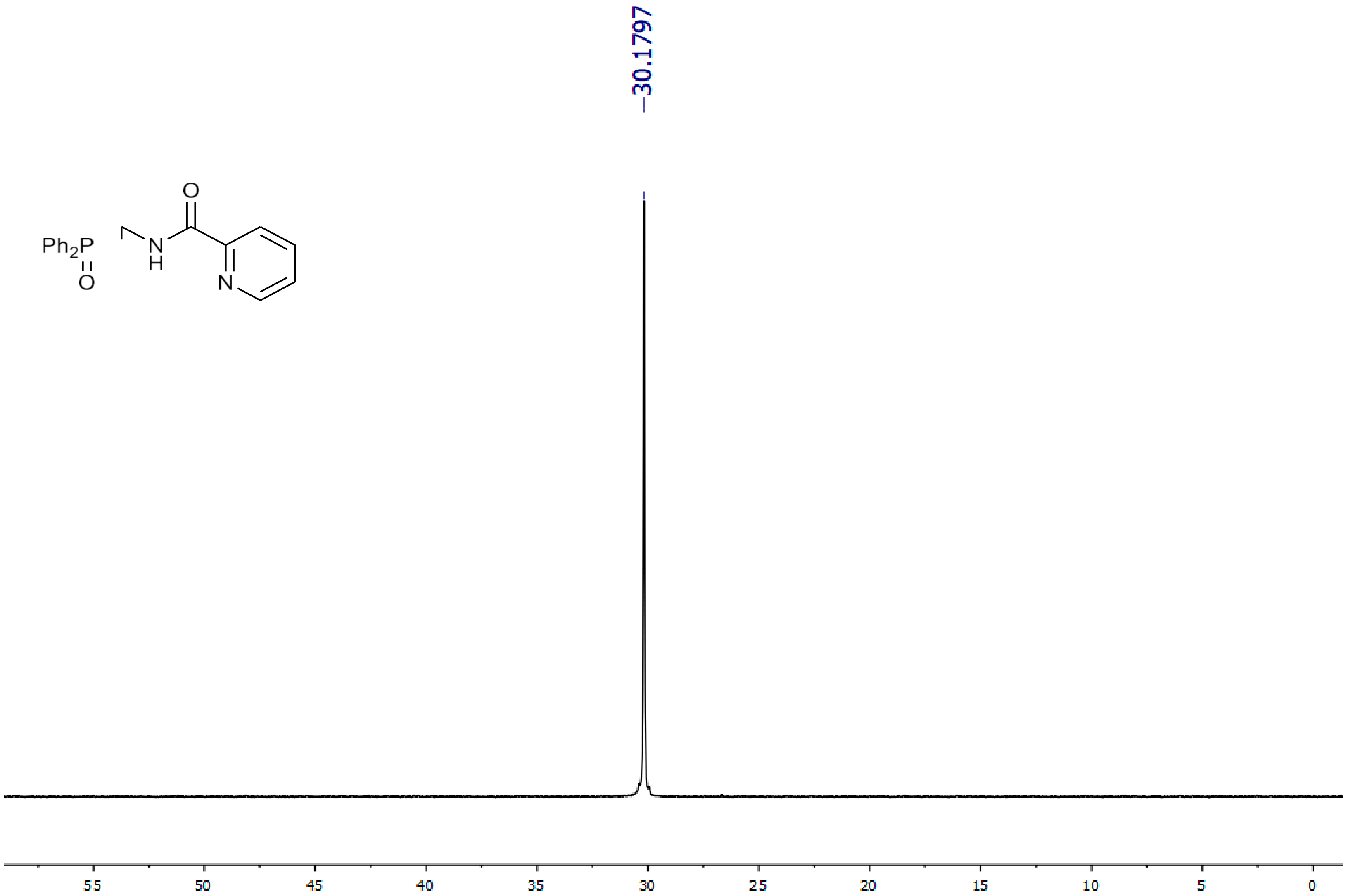

Figure S1. $^{31}P\{^{1}H\}$ NMR spectrum of ligand **1a** (202.45 MHz, $CDCl_3$).

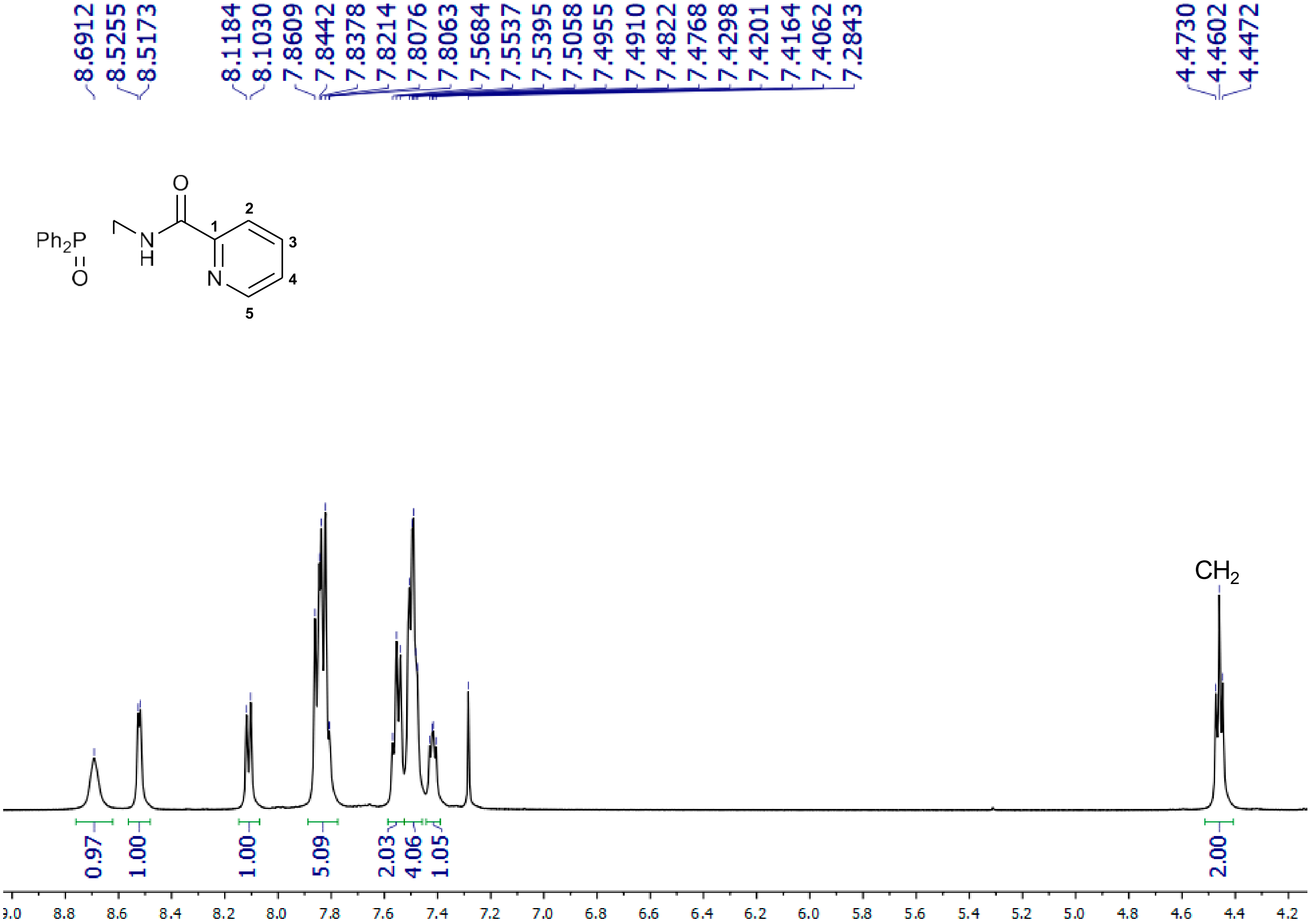

Figure. S2. ^{1}H NMR spectrum of ligand **1a** (500.13 MHz, CDCl3)

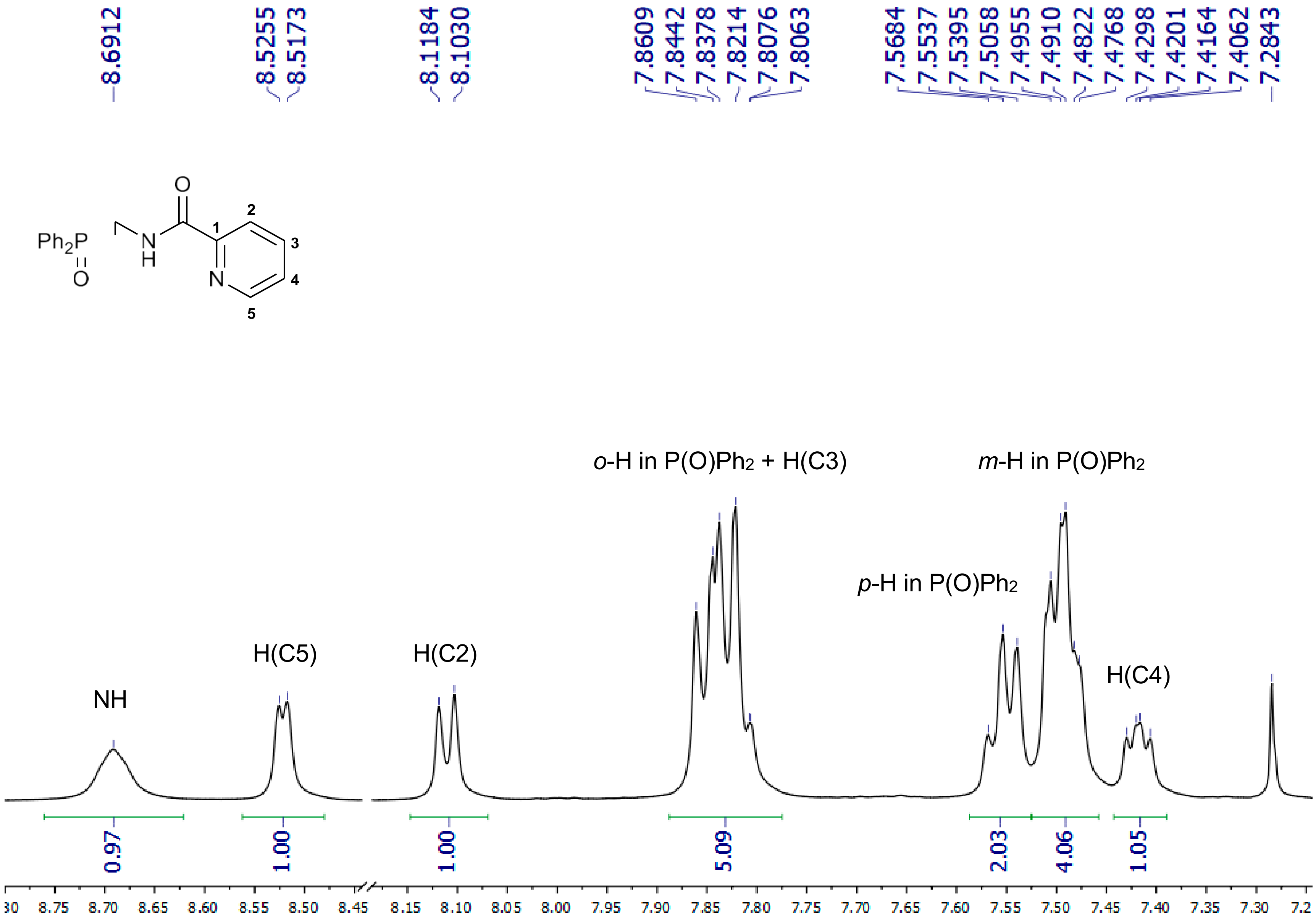

Figure. S3. Extended fragments of the ^{1}H NMR spectrum of ligand **1a** (500.13 MHz, CDCl3)

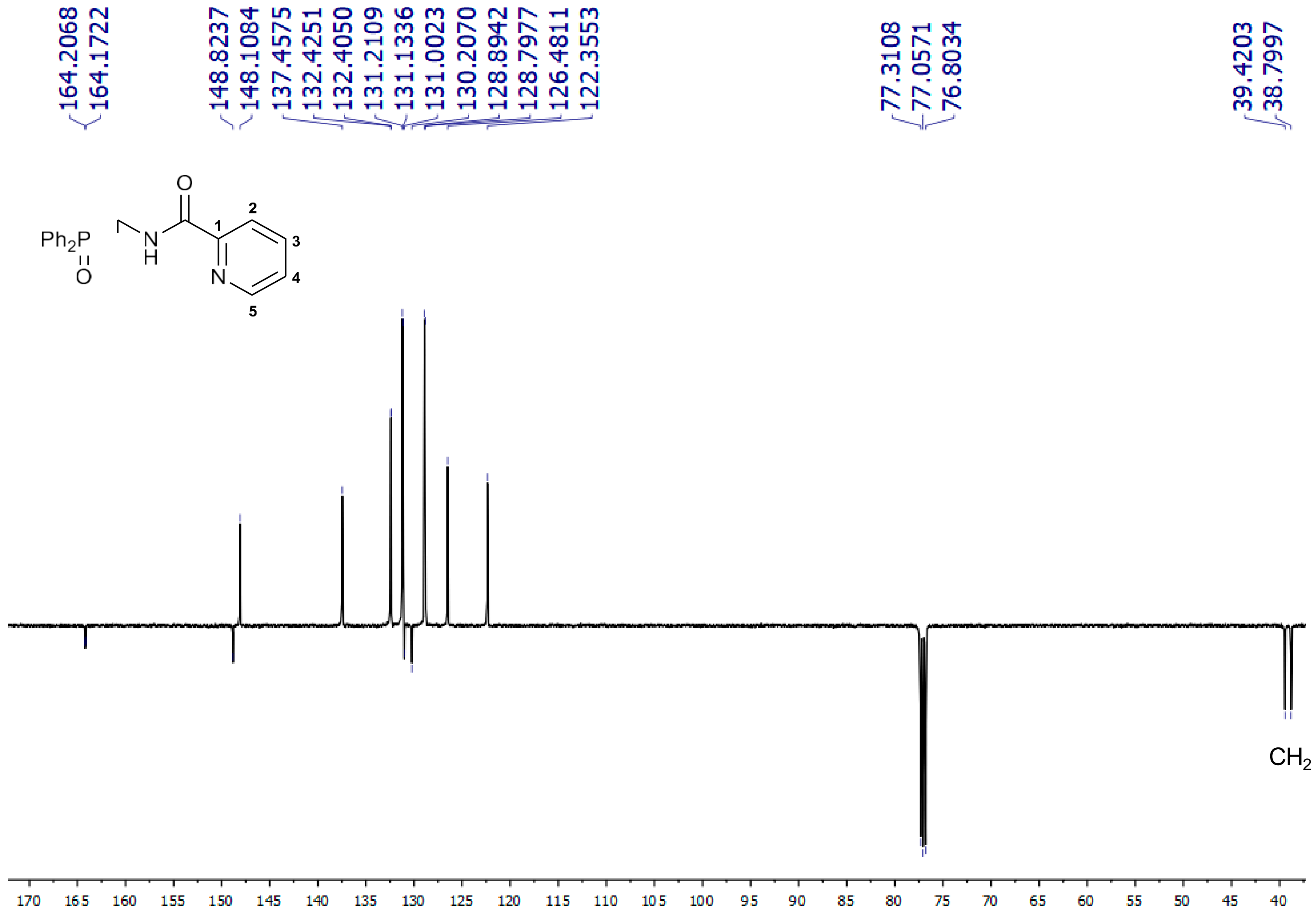

Figure. S4. $^{13}C\{^1H\}$ NMR spectrum of ligand **1a** (125.76 MHz, CDCl3

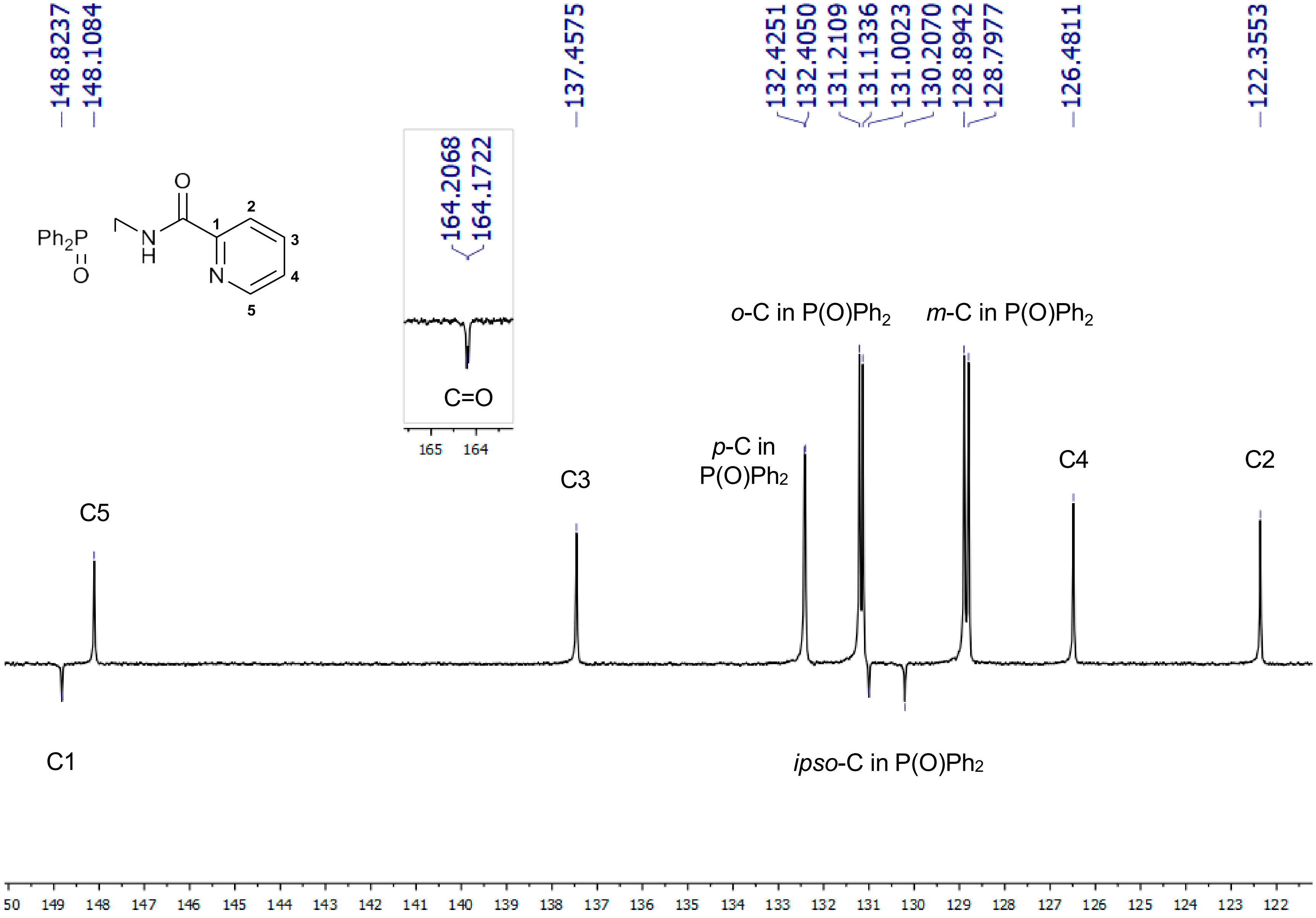

148.8237
148.1084
137.4575
132.4251
132.4050
131.2109
131.1336
131.0023
130.2070
128.8942
128.7977
126.4811
122.3553
164.2068
164.1722
C=O
165
164
Ph_2P
O
N
H
O
N
1
2
3
4
5
o-C in $P(O)Ph_2$
m-C in $P(O)Ph_2$
p-C in $P(O)Ph_2$
C5
C3
C4
C2
C1
ipso-C in $P(O)Ph_2$
50 149 148 147 146 145 144 143 142 141 140 139 138 137 136 135 134 133 132 131 130 129 128 127 126 125 124 123 122

Figure. S5. Extended fragments of the $^{13}C\{^1H\}$ NMR spectrum of ligand **1a** (125.76 MHz, CDCl3

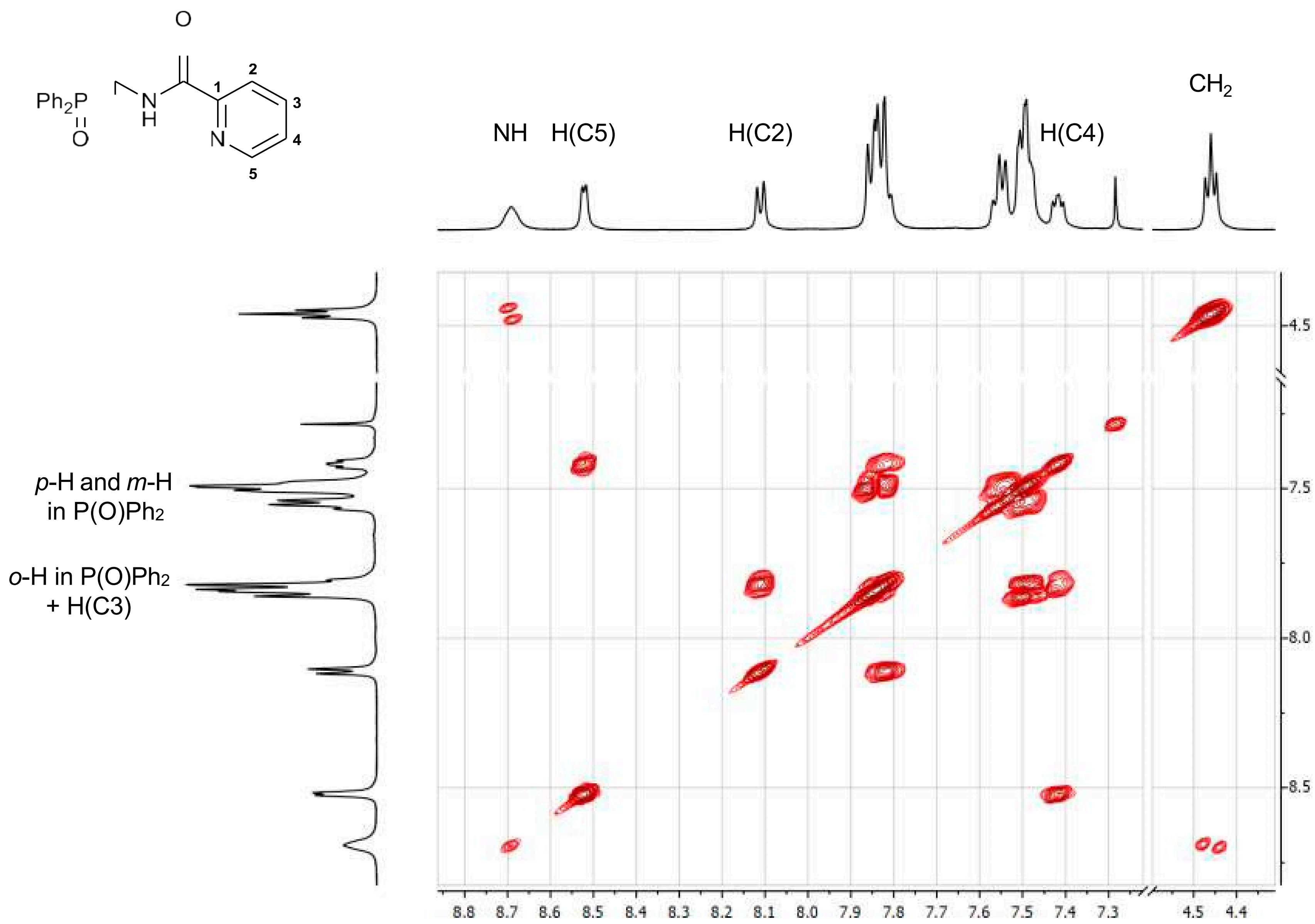

Figure. S6. Extended fragments of the ^{1}H–^{1}H COSY spectrum of ligand **1a** (500.13 MHz, $CDCl_3$)

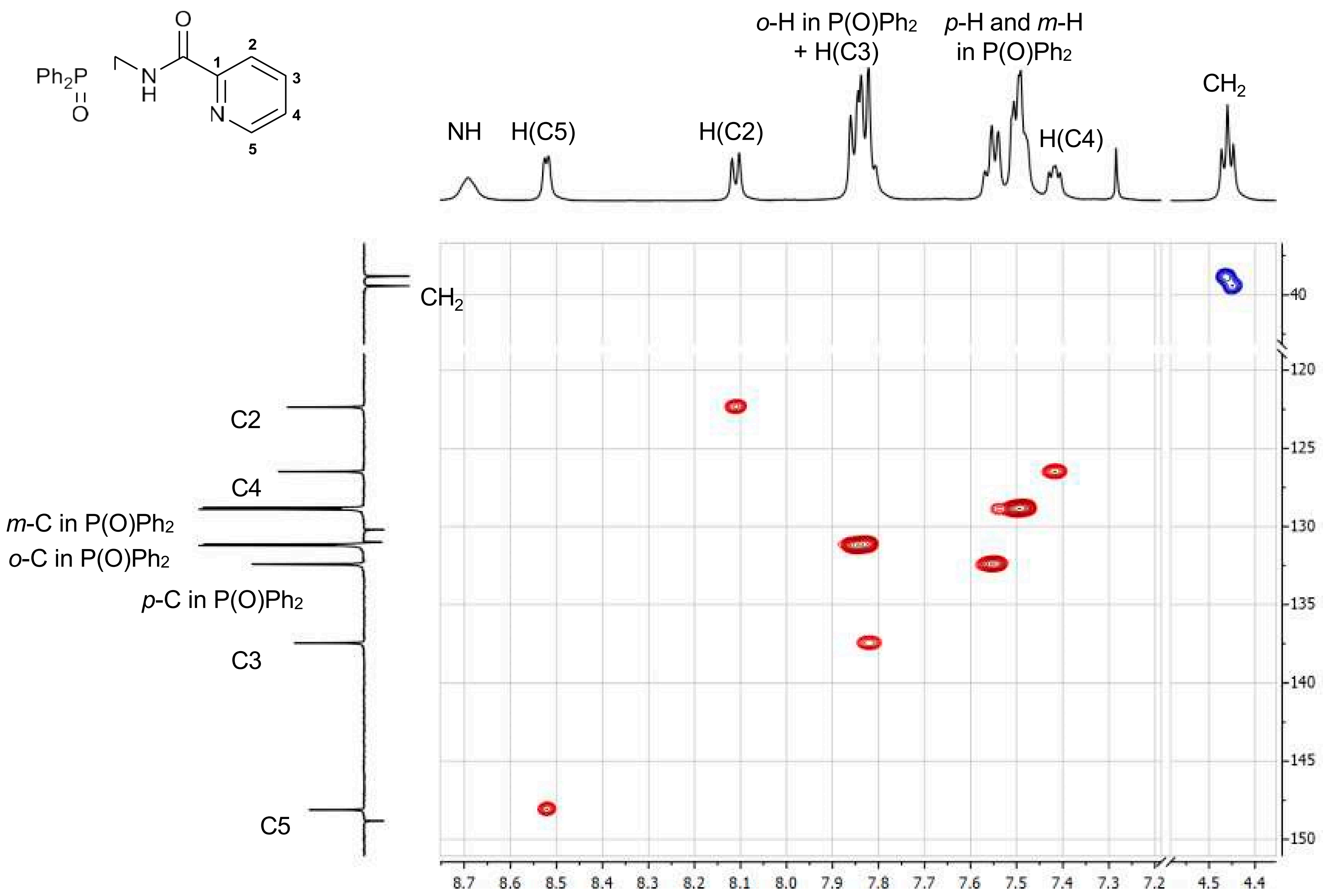

Figure. S7. Extended fragments of the ^{1}H–^{13}C HSQC spectrum of ligand **1a** ($CDCl_3$)

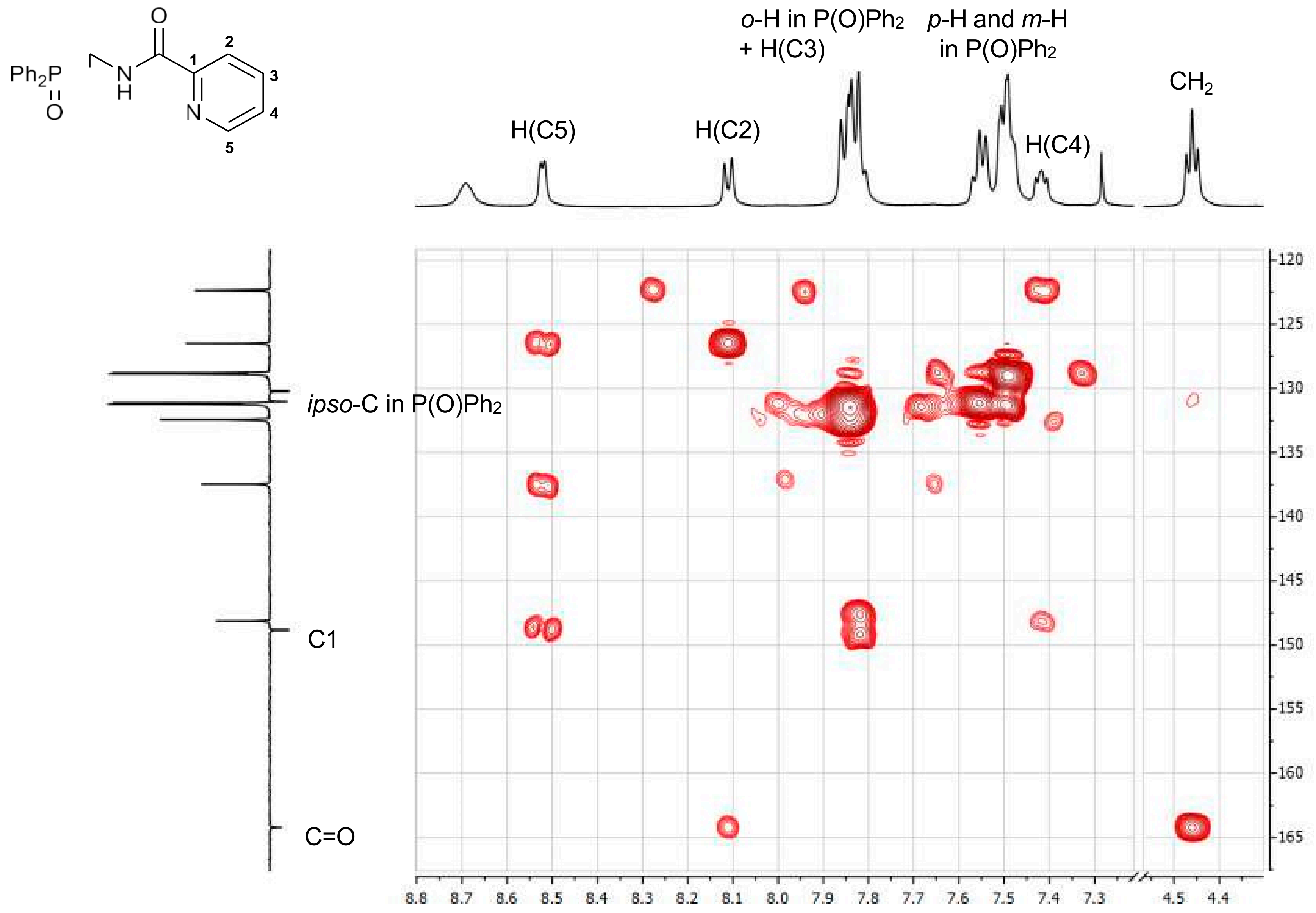

Figure. S8. Extended fragments of the ^1H–^{13}C HMBC spectrum of ligand **1a** ($CDCl_3$)

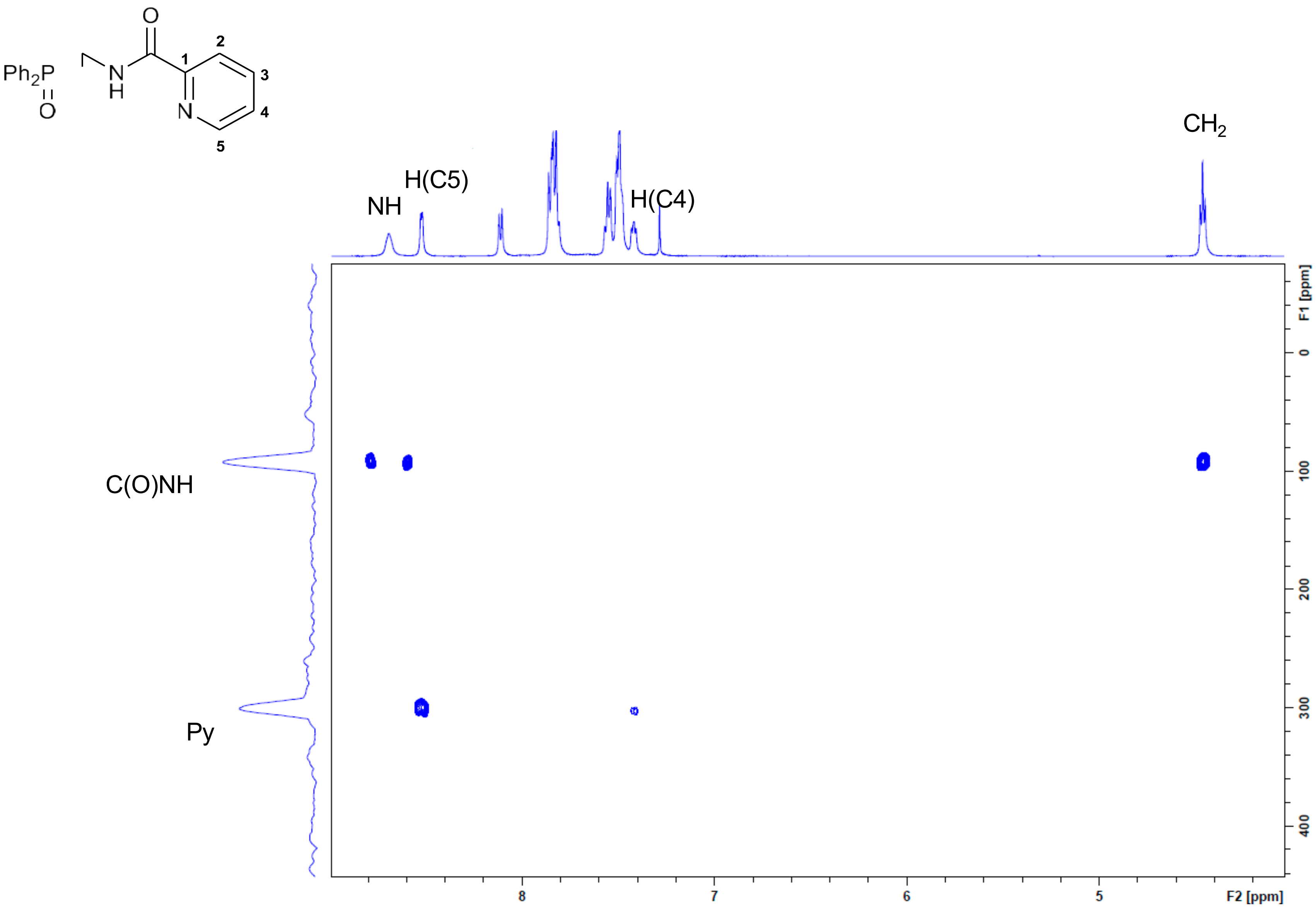

Figure. S9. ^1H–^{15}N HMBC spectrum of ligand **1a** ($CDCl_3$)

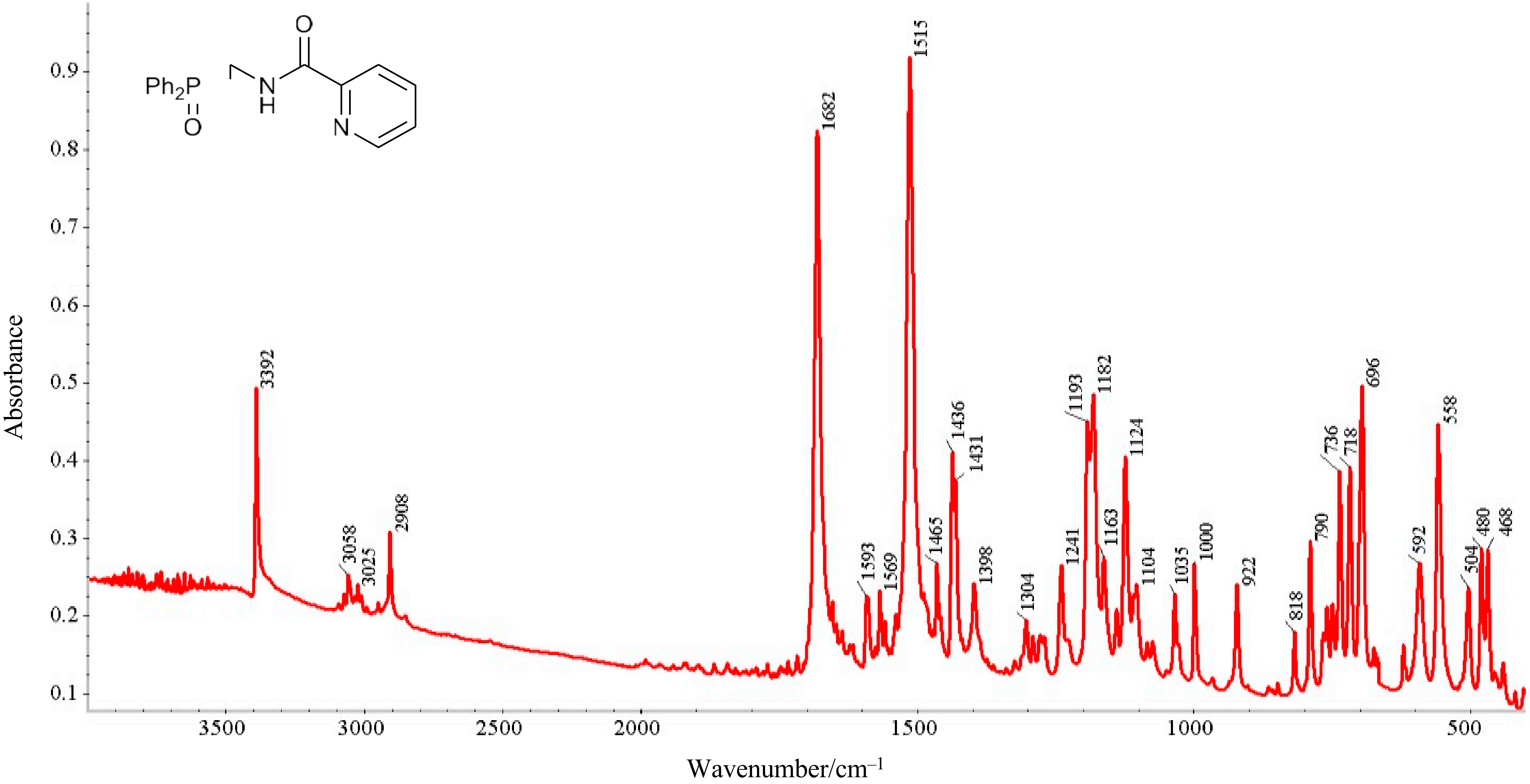

Figure. S10. IR spectrum of ligand **1a**

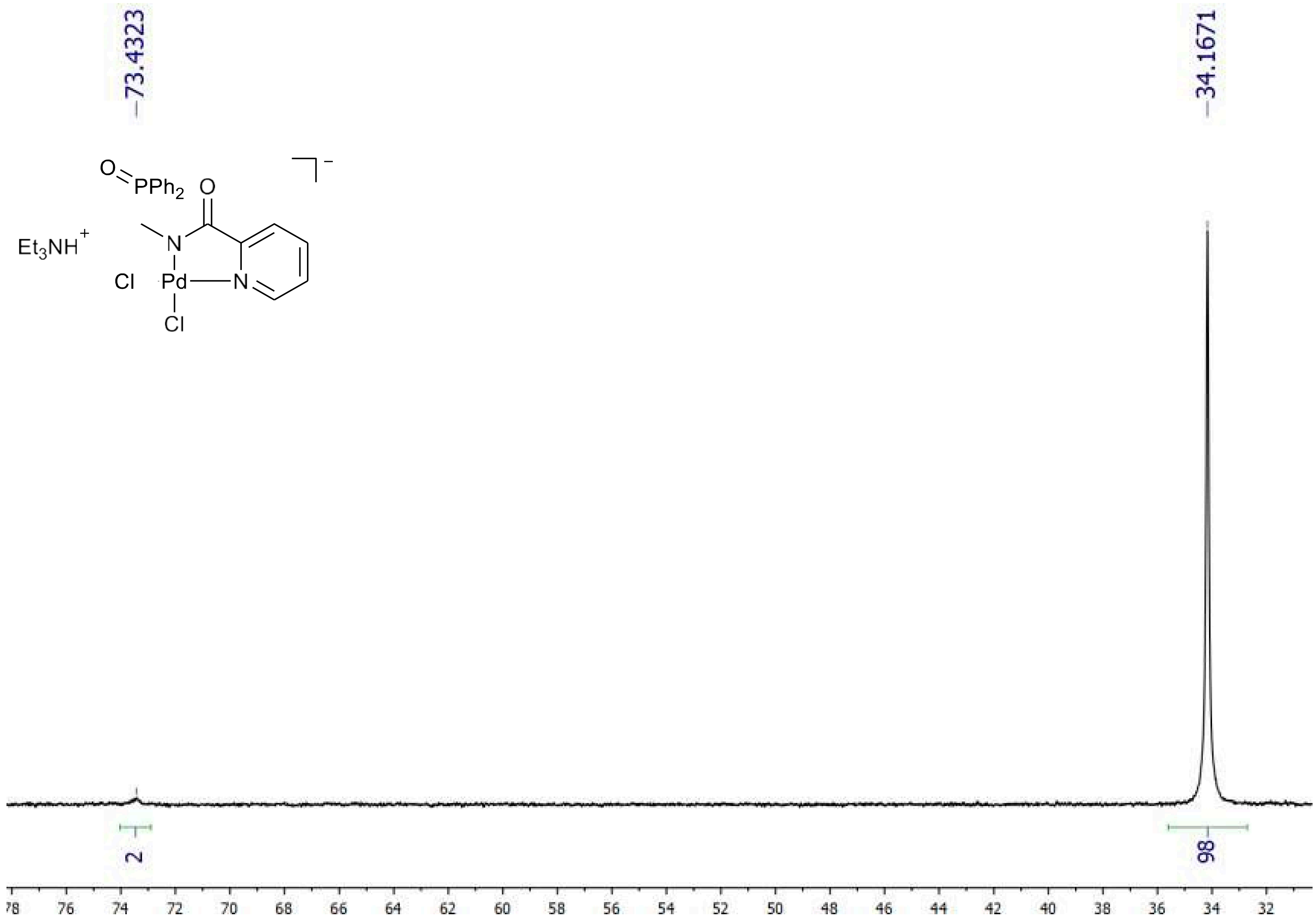

Figure S11. $^{31}P\{^{1}H\}$ NMR spectrum of complex **2a** (202.45 MHz, $CDCl_3$

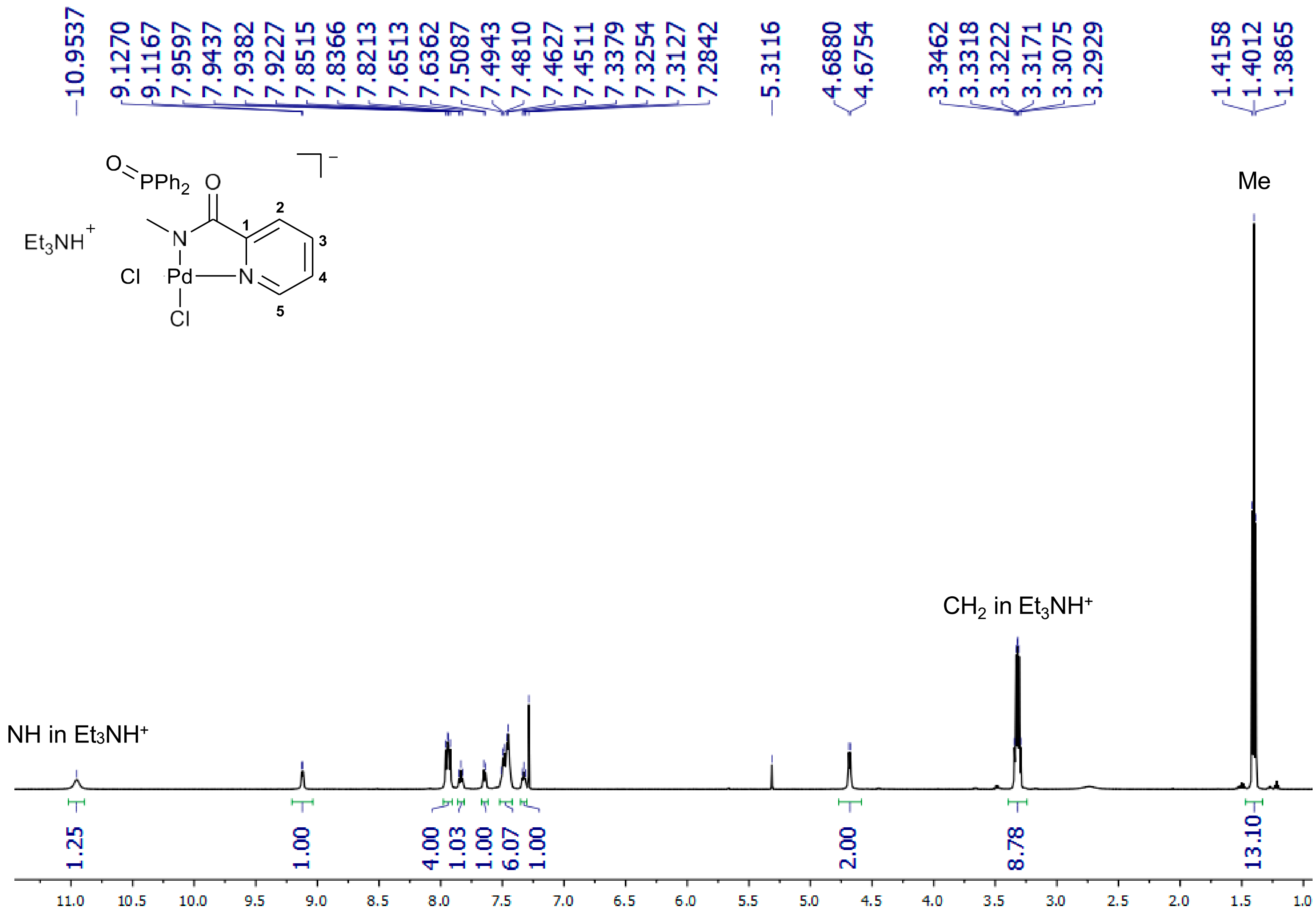

Figure S12. ^{1}H NMR spectrum of complex **2a** (500.13 MHz, $CDCl_3$)

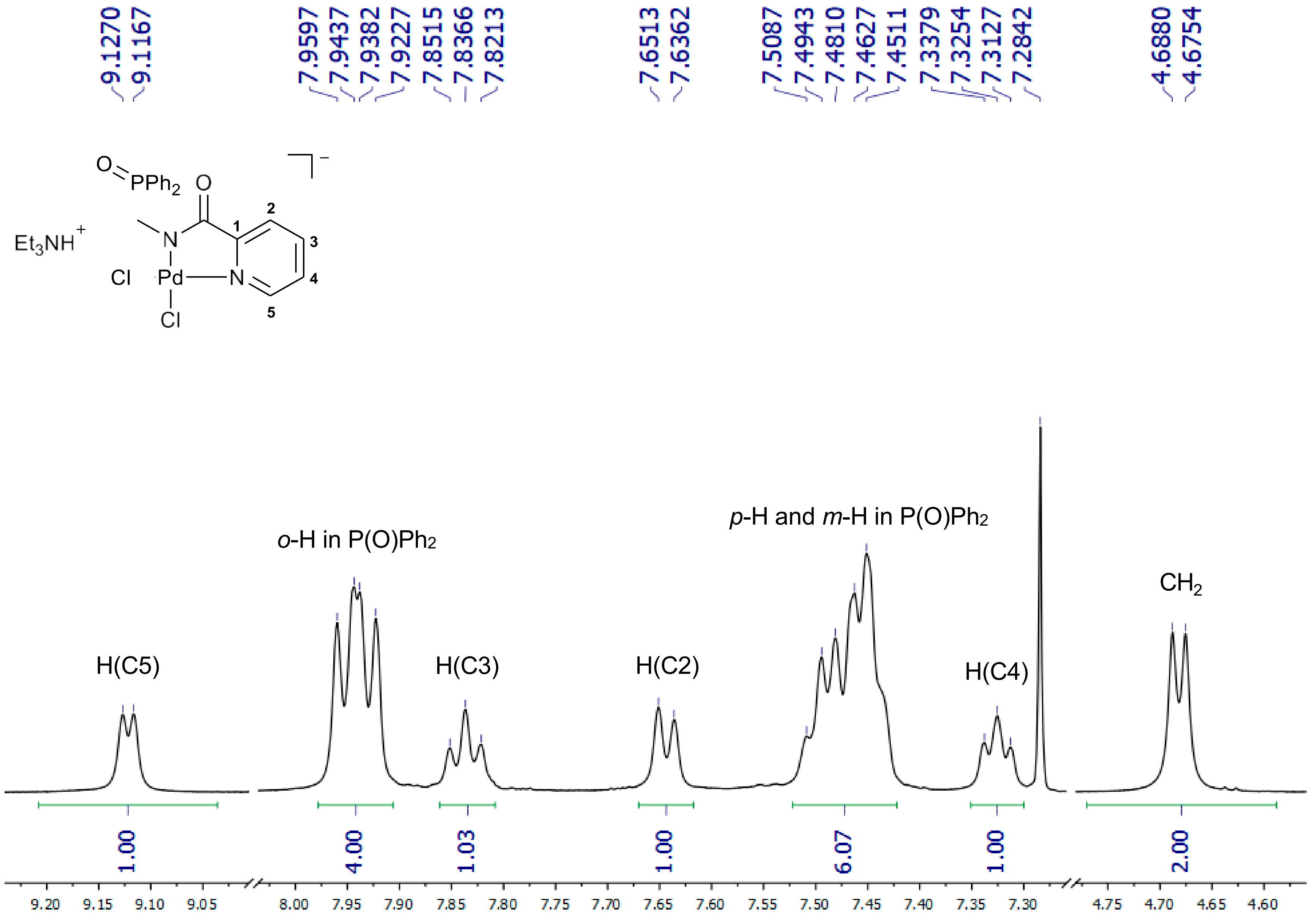

Figure. S13. Extended fragments of the ^{1}H NMR spectrum of complex **2a** (500.13 MHz, $CDCl_3$)

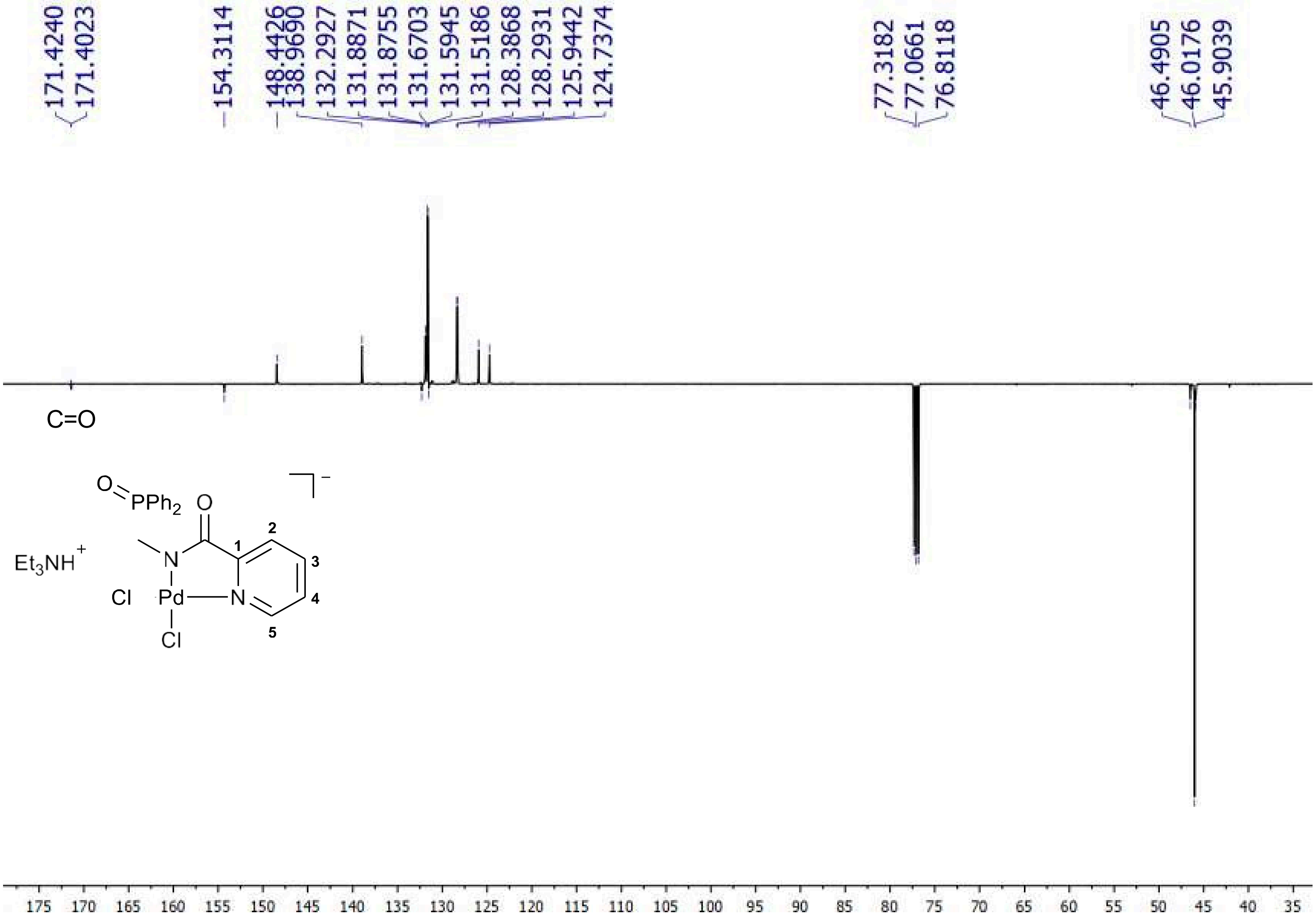

Figure S14. $^{13}C\{^1H\}$ NMR spectrum of complex **2a** (125.76 MHz, $CDCl_3$)

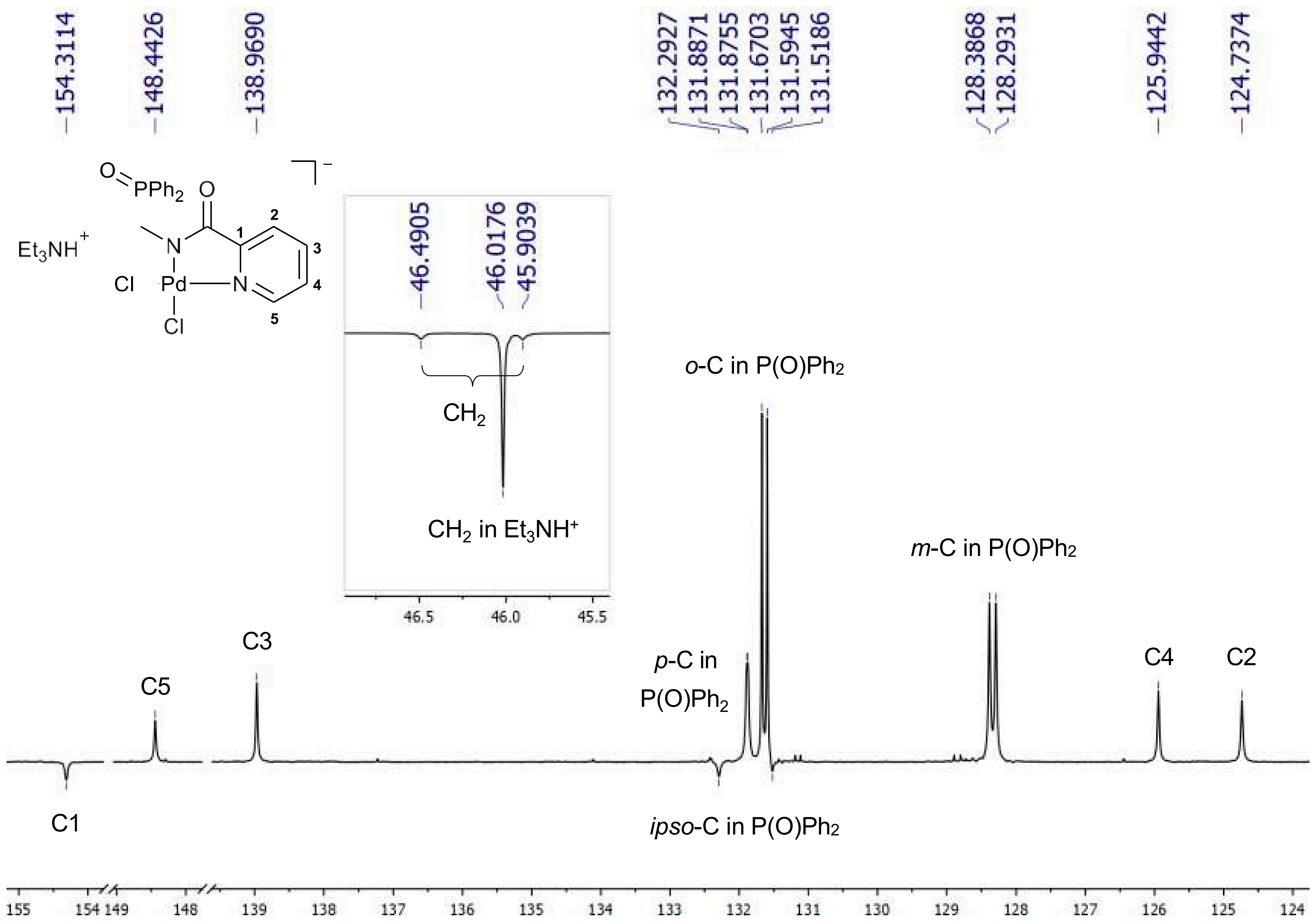

Fig. S15. Extended fragments of the $^{13}C\{^1H\}$ NMR spectrum of complex **2a** (125.76 MHz, $CDCl_3$)

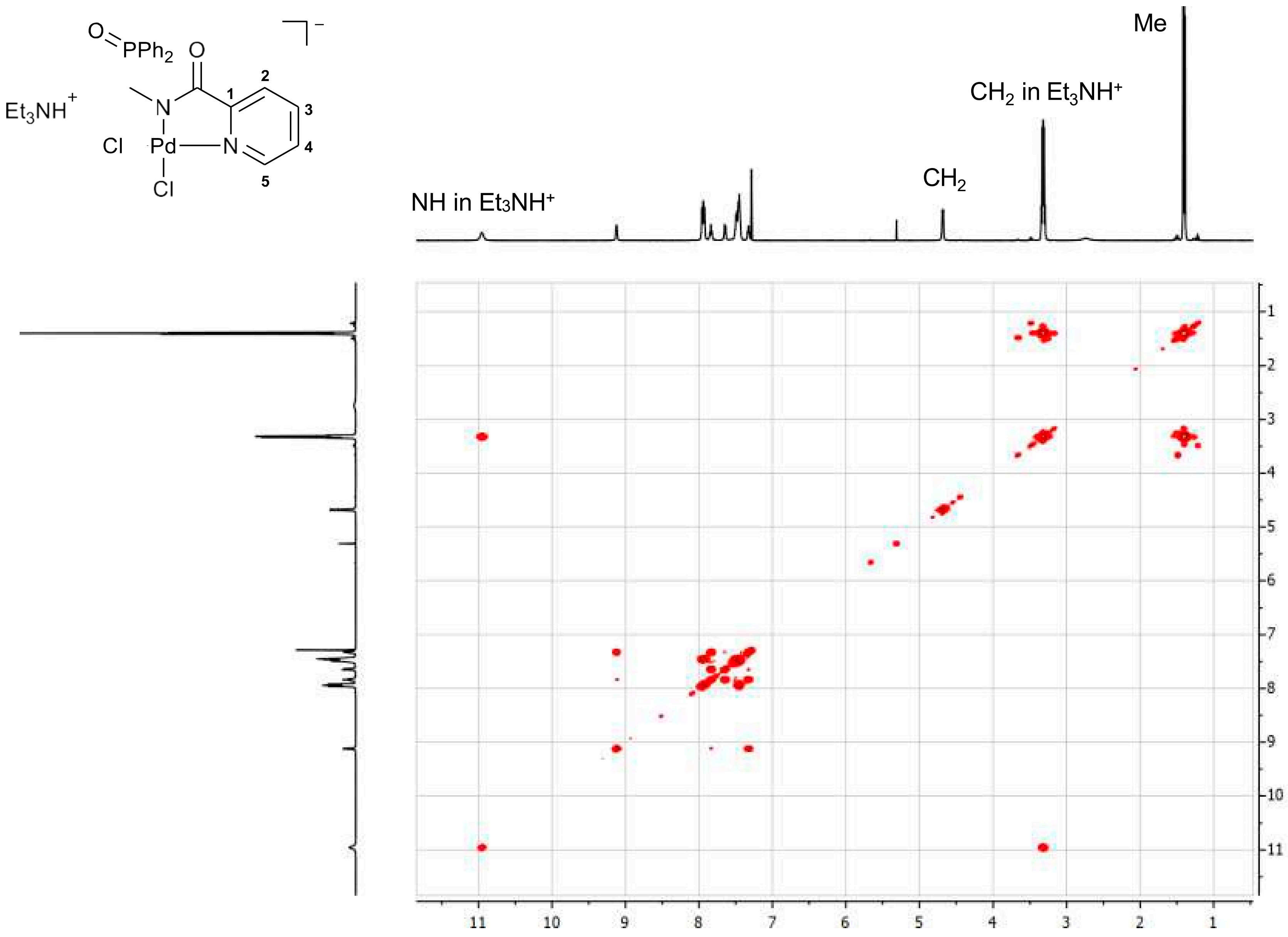

Figure. S16. ^1H–^1H COSY spectrum of complex **2a** (500.13 MHz, $CDCl_3$)

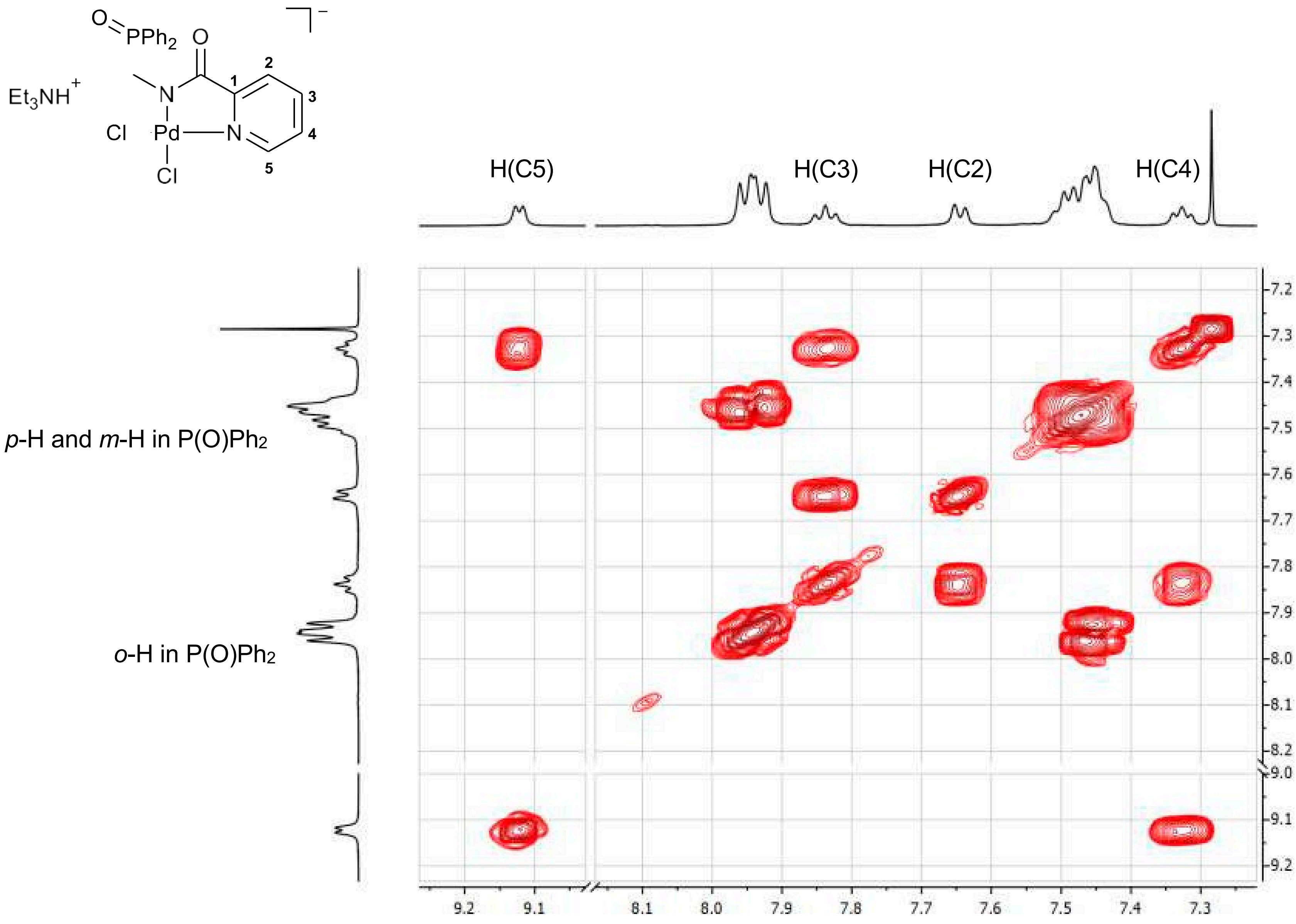

Figure S17. ^{1}H–^{1}H COSY spectrum of complex **2a** (500.13 MHz, $CDCl_3$)

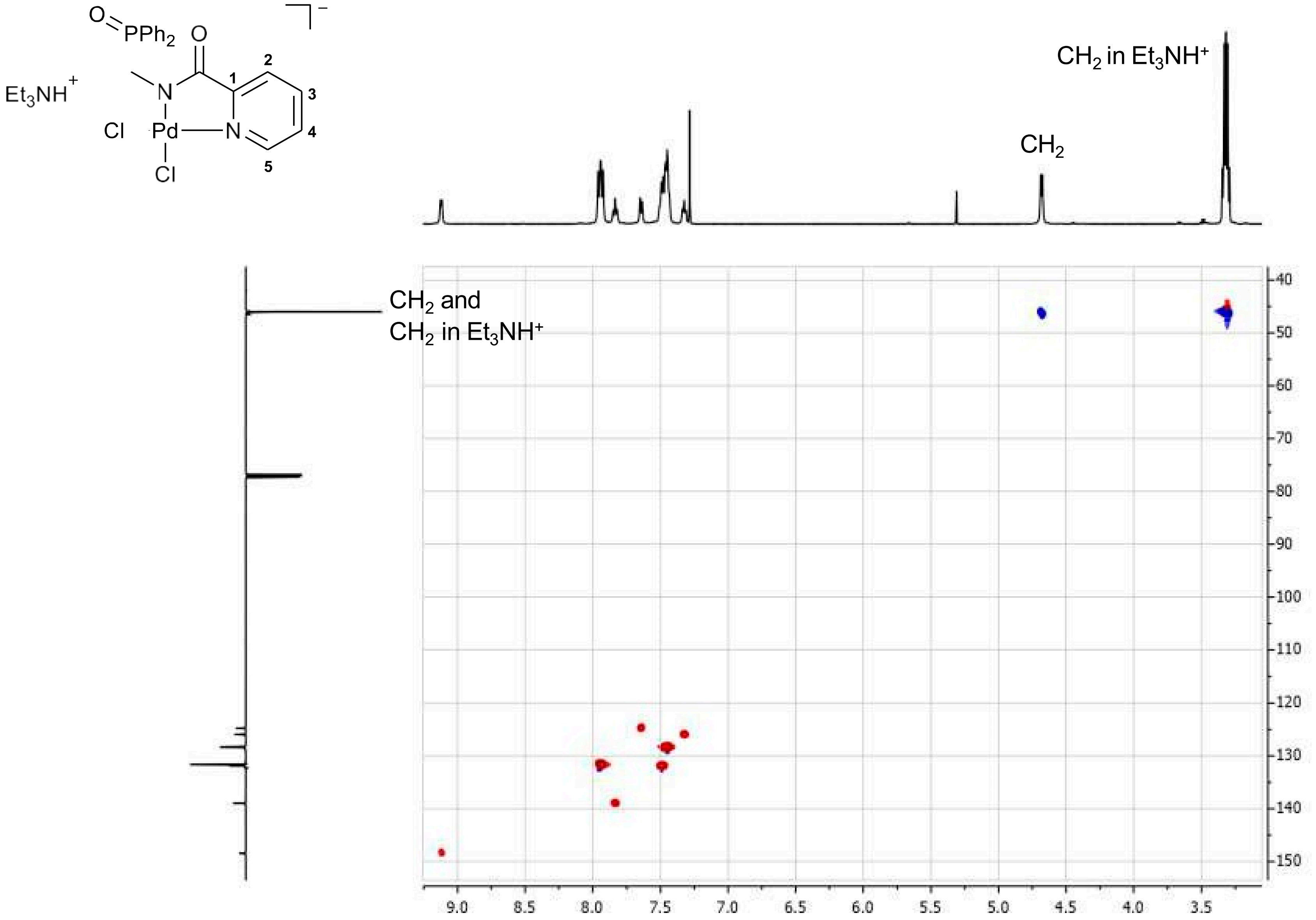

Figure S18. ^{1}H–^{13}C HSQC spectrum of complex **2a** ($CDCl_3$)

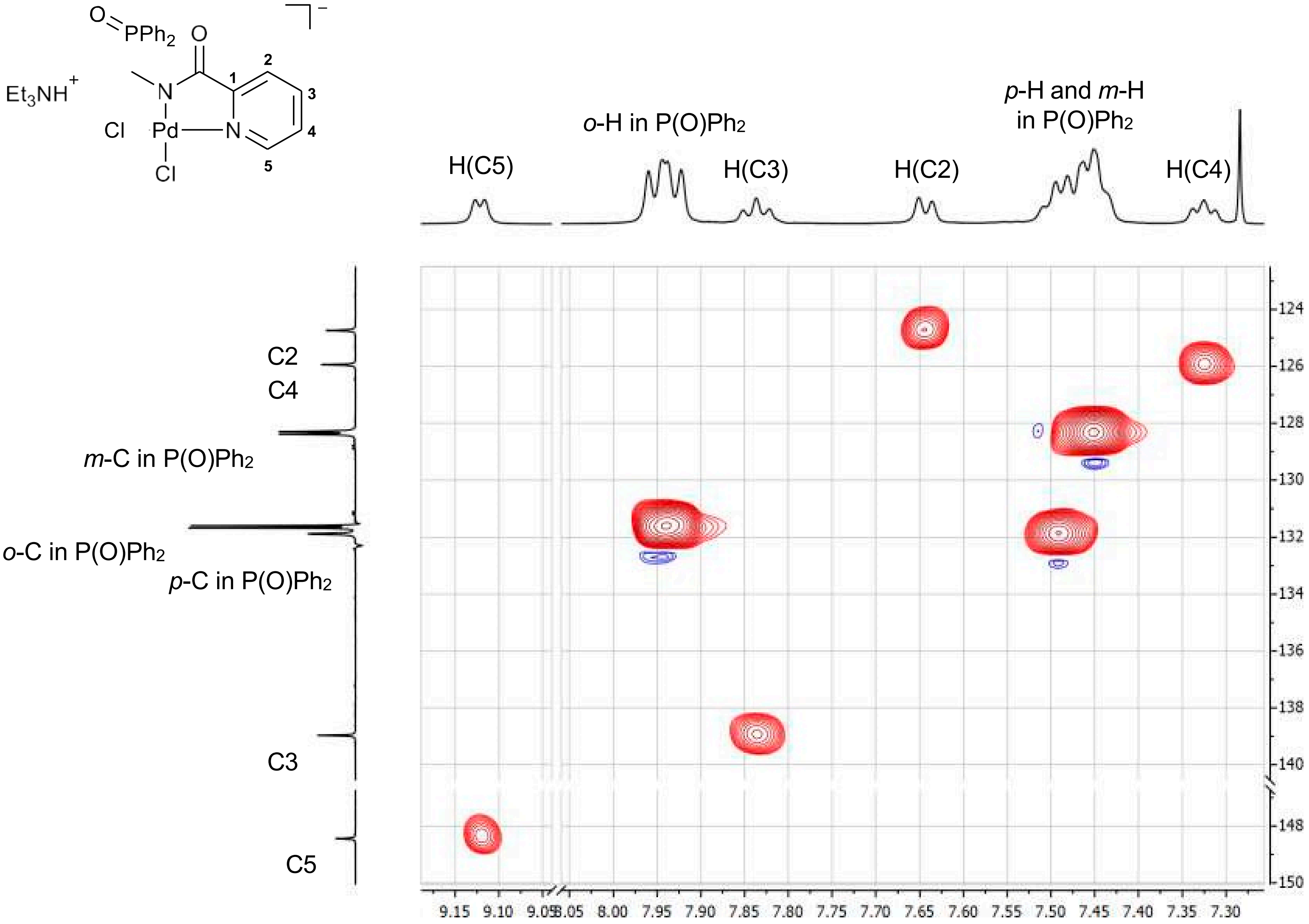

Figure S19. Extended fragments of the ^1H–^{13}C HSQC spectrum of complex **2a** ($CDCl_3$)

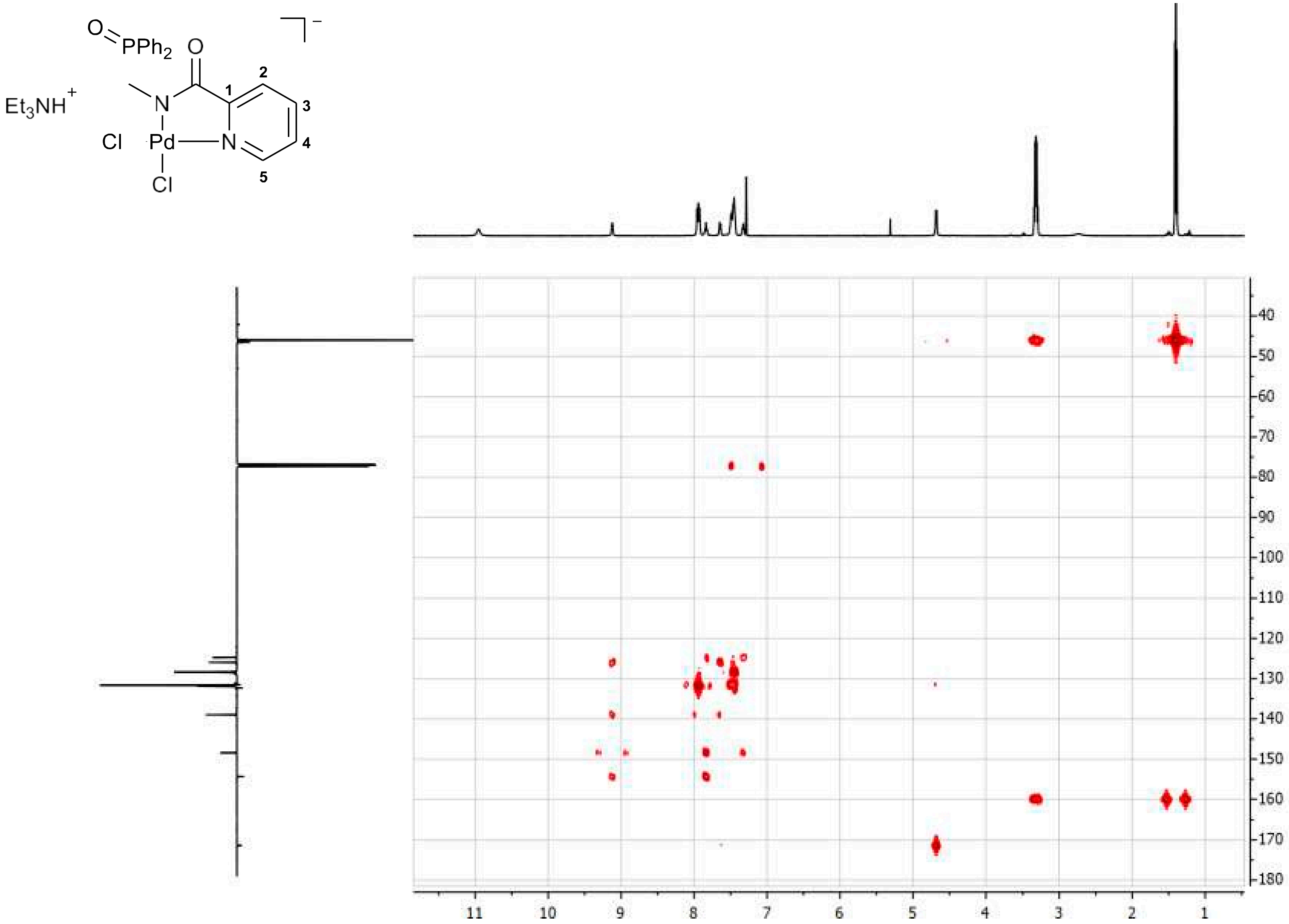

Figure S20. ^{1}H–^{13}C HMBC spectrum of complex **2a** ($CDCl_3$)

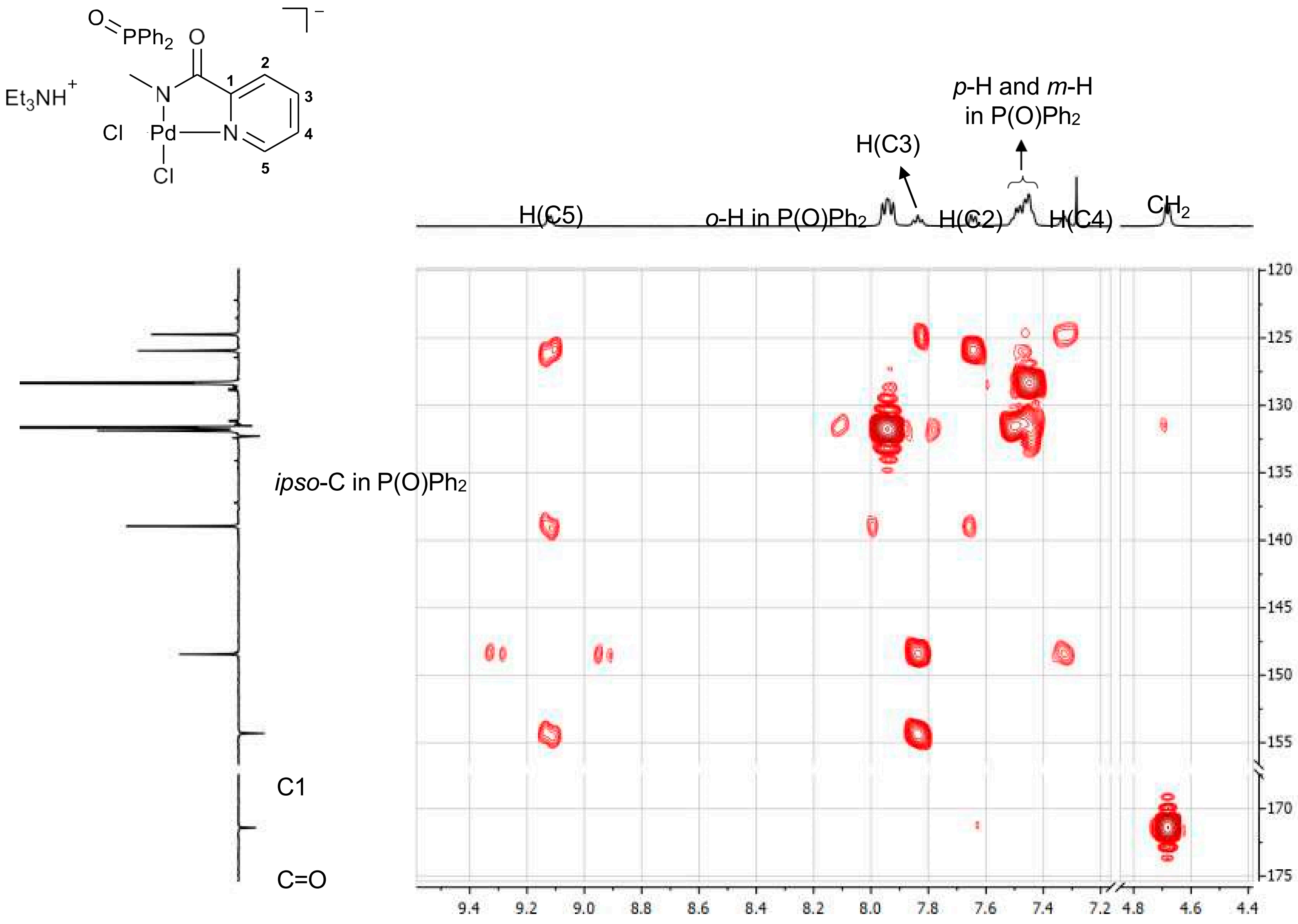

O=PPh2
O
Et3NH+
N
Pd
N
Cl
Cl
1
2
3
4
5
p-H and m-H in P(O)Ph2
H(C3)
H(C5)
o-H in P(O)Ph2
H(C2)
H(C4)
CH2
ipso-C in P(O)Ph2
C1
C=O

Figure S21. Extended fragments of the ^{1}H–^{13}C HMBC spectrum of complex **2a** ($CDCl_3$)

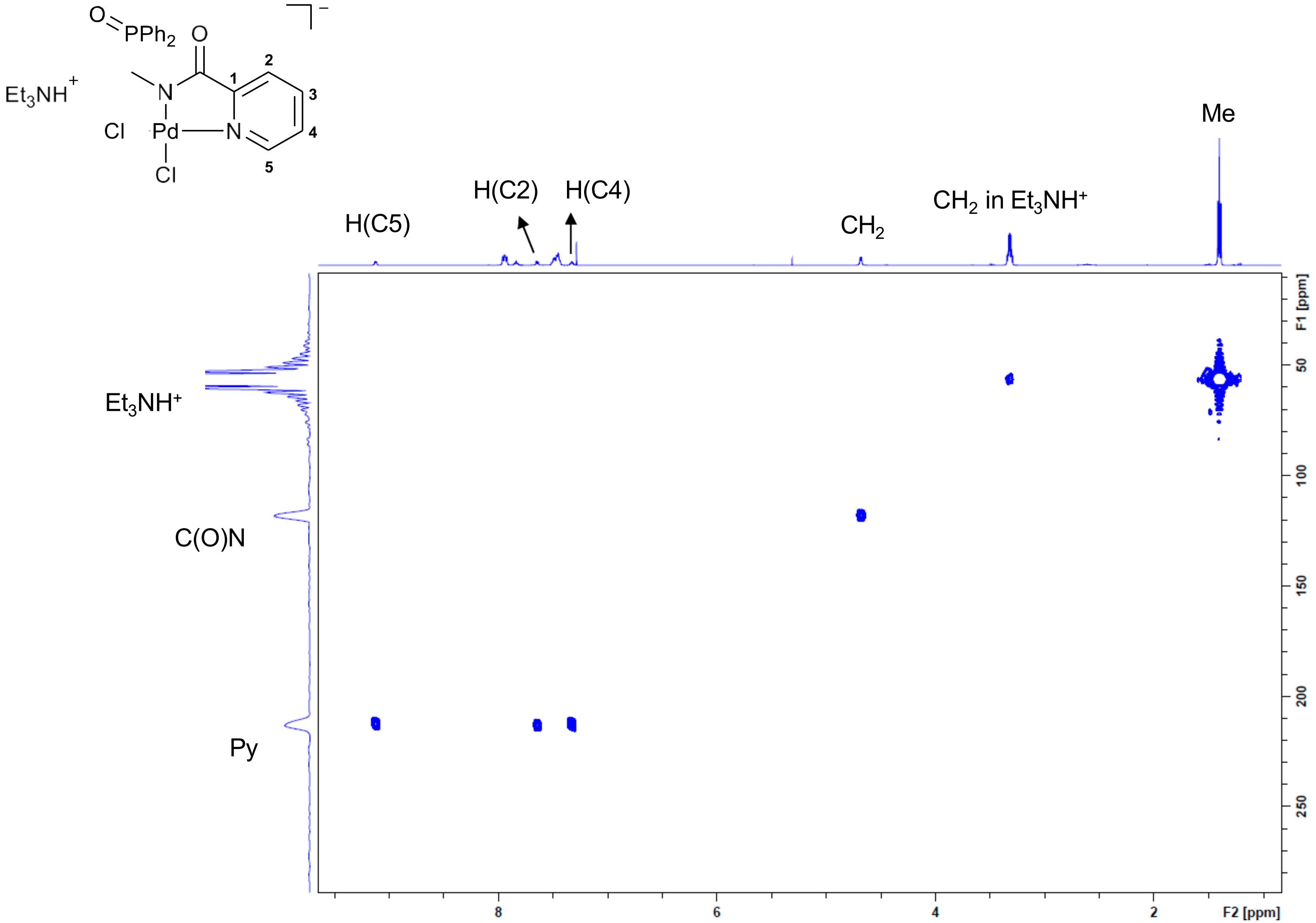

O
PPh2
O
1
2
3
4
5
Et3NH+
N
Pd
N
Cl
Cl
Me
H(C5)
H(C2)
H(C4)
CH2
CH2 in Et3NH+
Et3NH+
C(O)N
Py
F1 [ppm]
50
100
150
200
250
8
6
4
2
F2 [ppm]

Figure S22. ^{1}H–^{15}N HMBC spectrum of complex **2a** ($CDCl_3$)

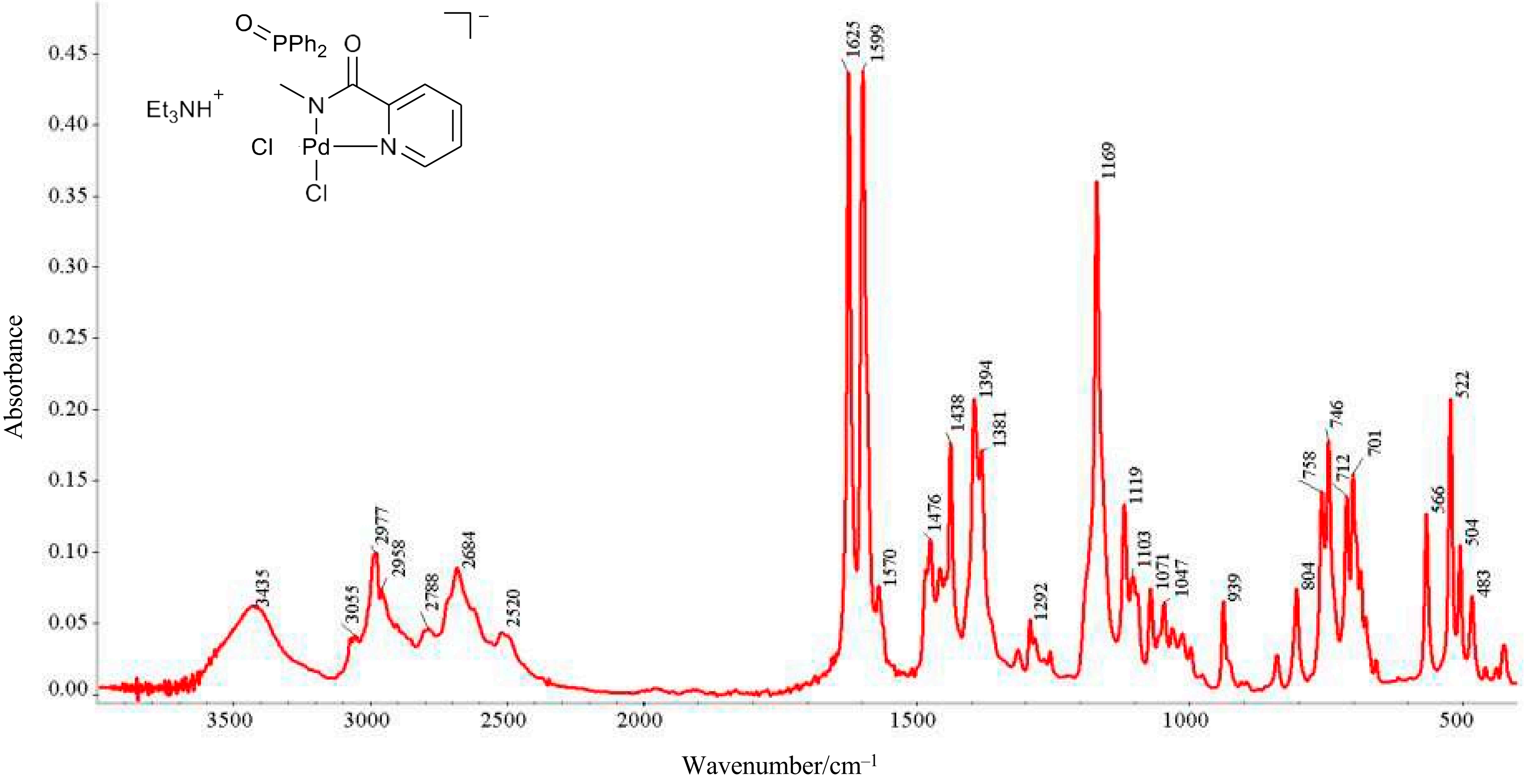

Figure. S23. IR spectrum of complex **2a**

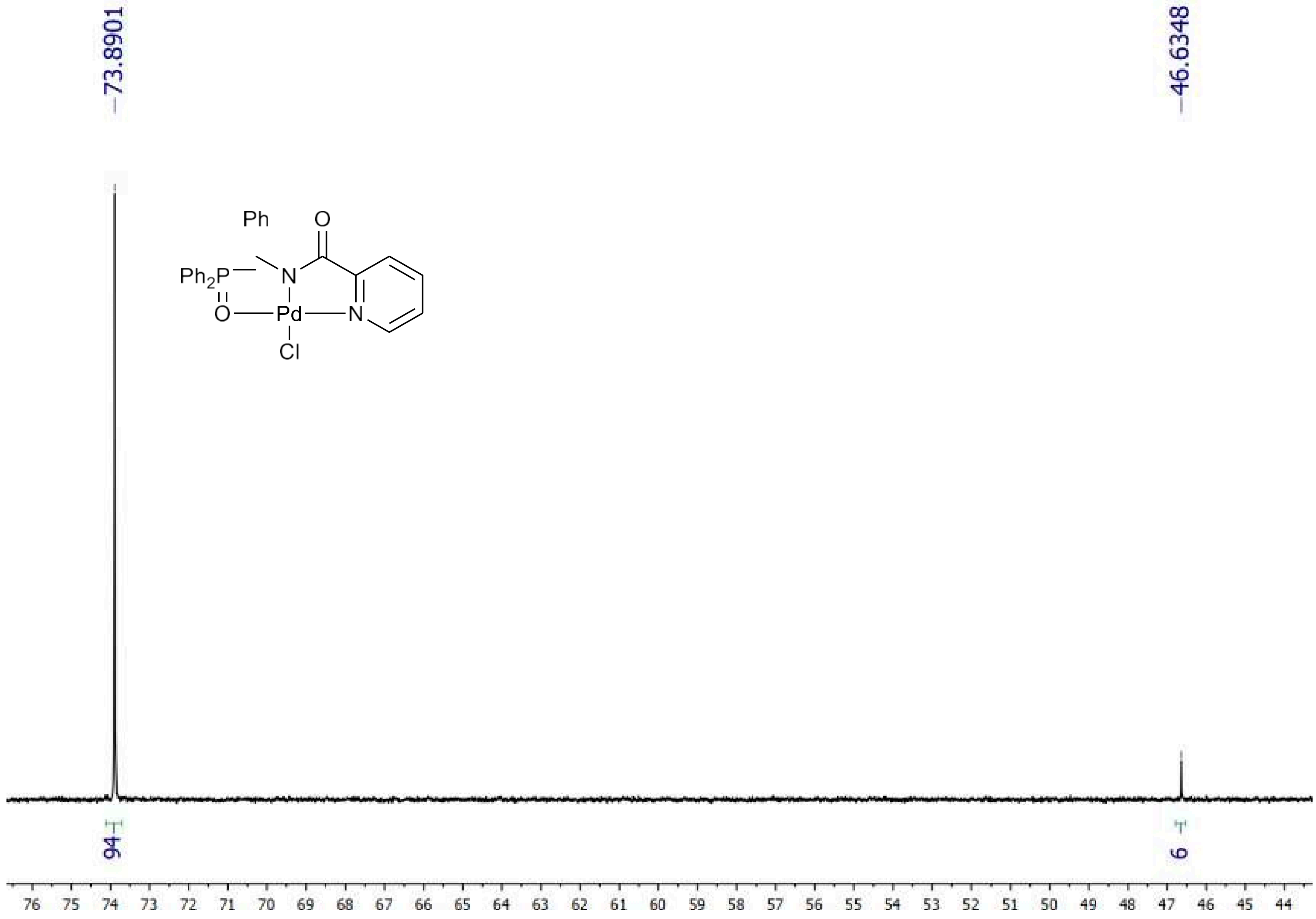

Figure S24. $^{31}P\{^1H\}$ NMR spectrum of complex **3b** (202.45 MHz, $CDCl_3$)

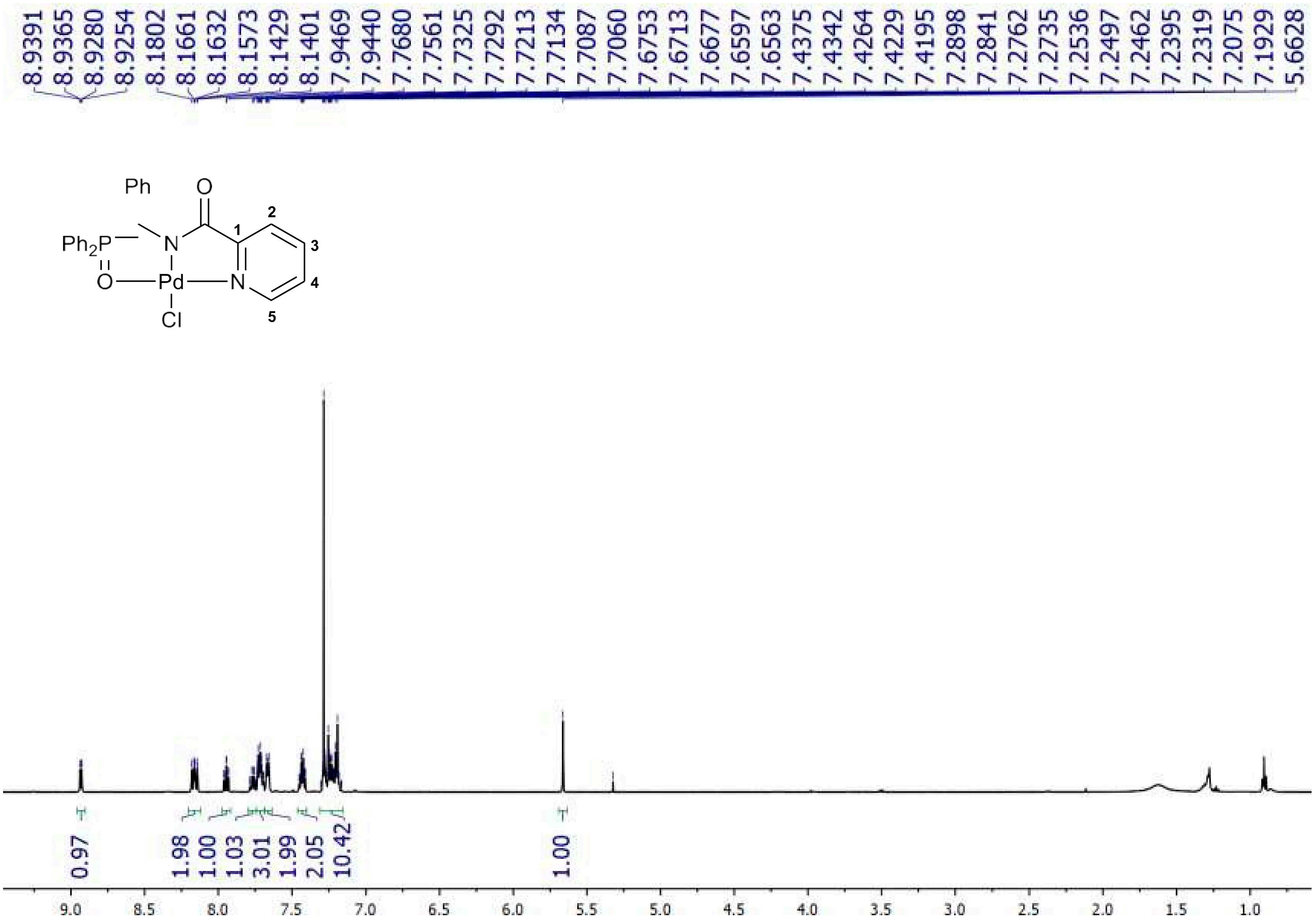

Figure S25. ^{1}H NMR spectrum of complex **3b** (500.13 MHz, $CDCl_3$)

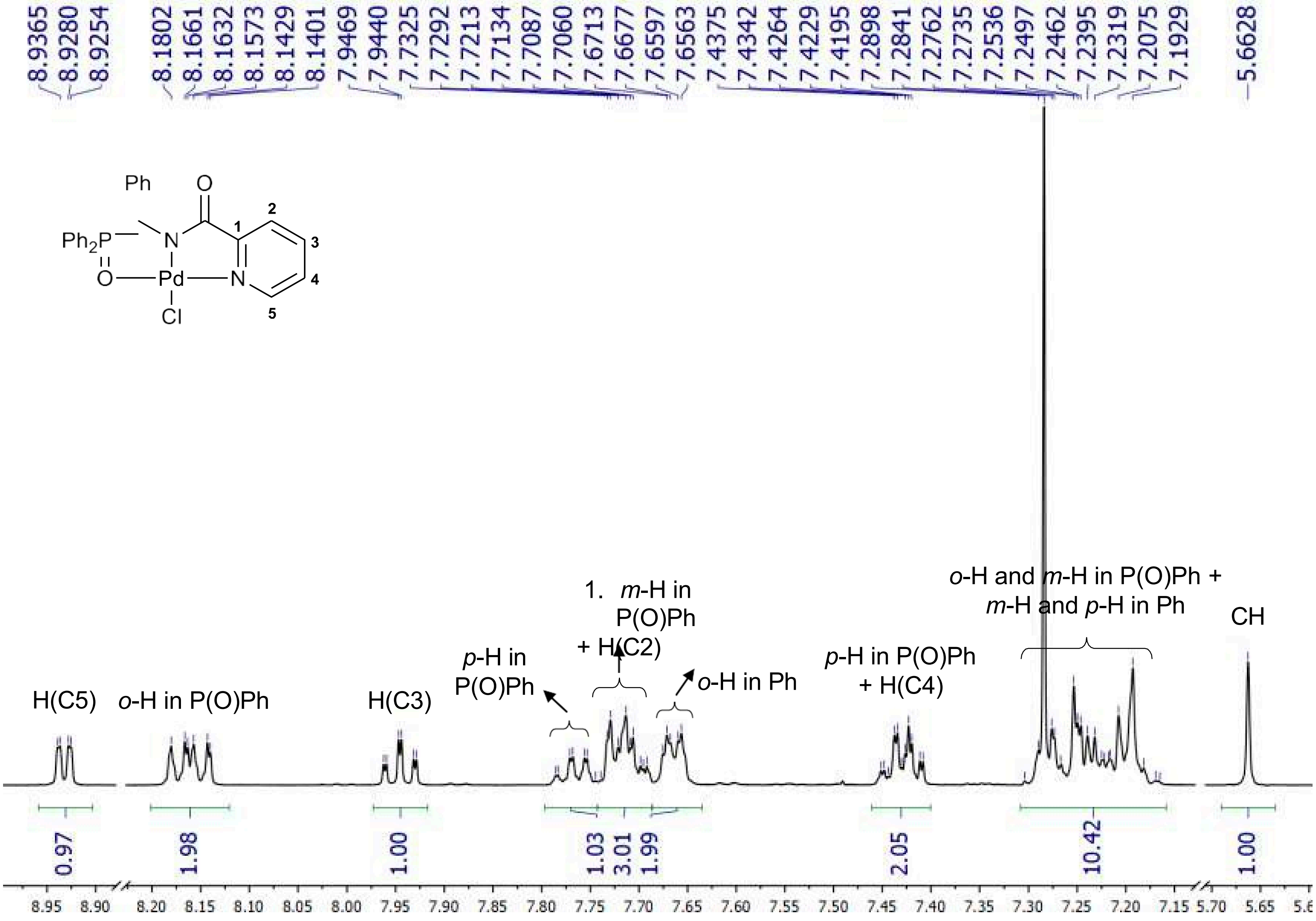

Figure S26. Extended fragments of the ^{1}H NMR spectrum of complex **3b** (500.13 MHz, CDCl3)

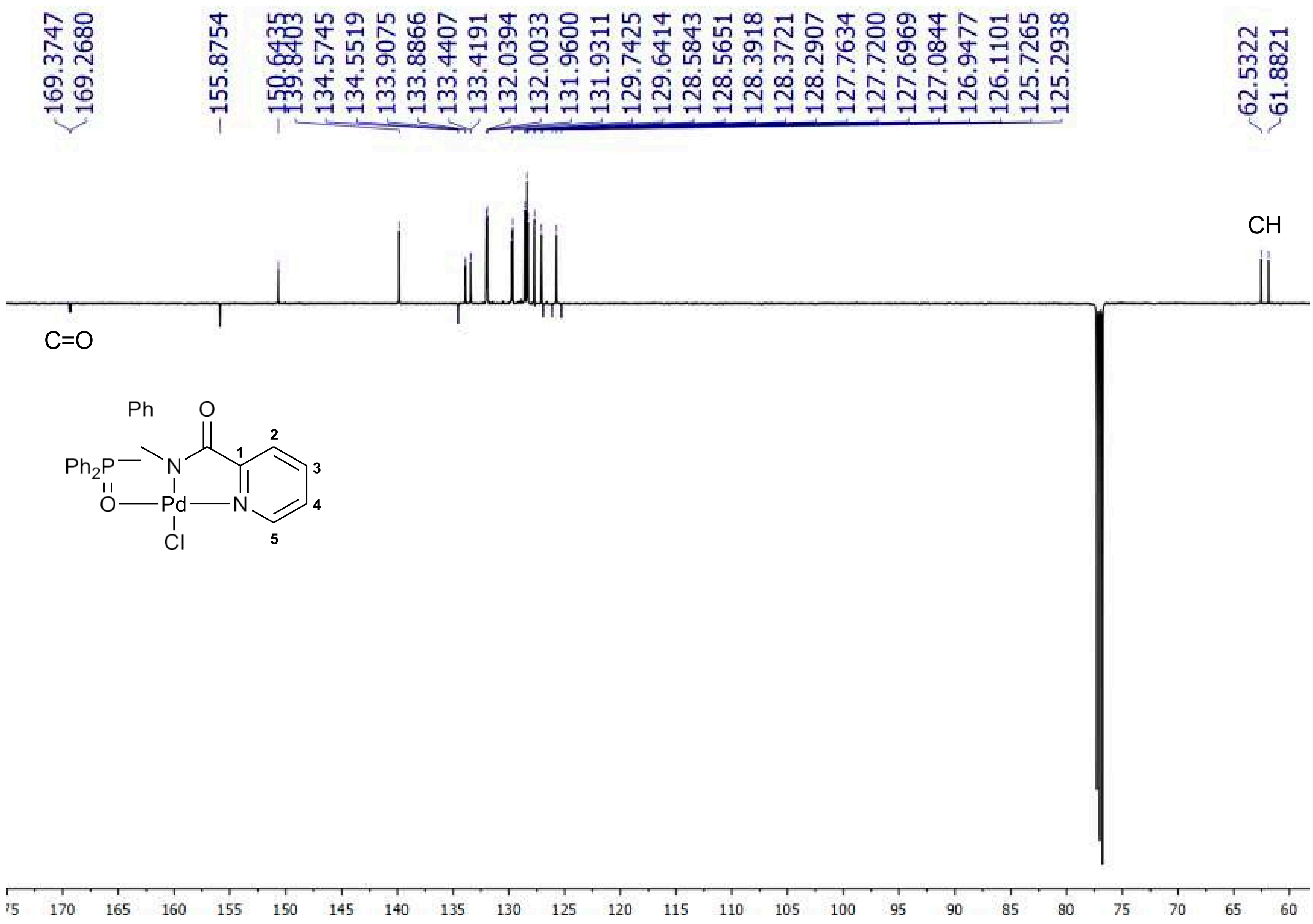

Figure S27. $^{13}C\{^{1}H\}$ NMR spectrum of complex **3b** (125.76 MHz, $CDCl_3$)

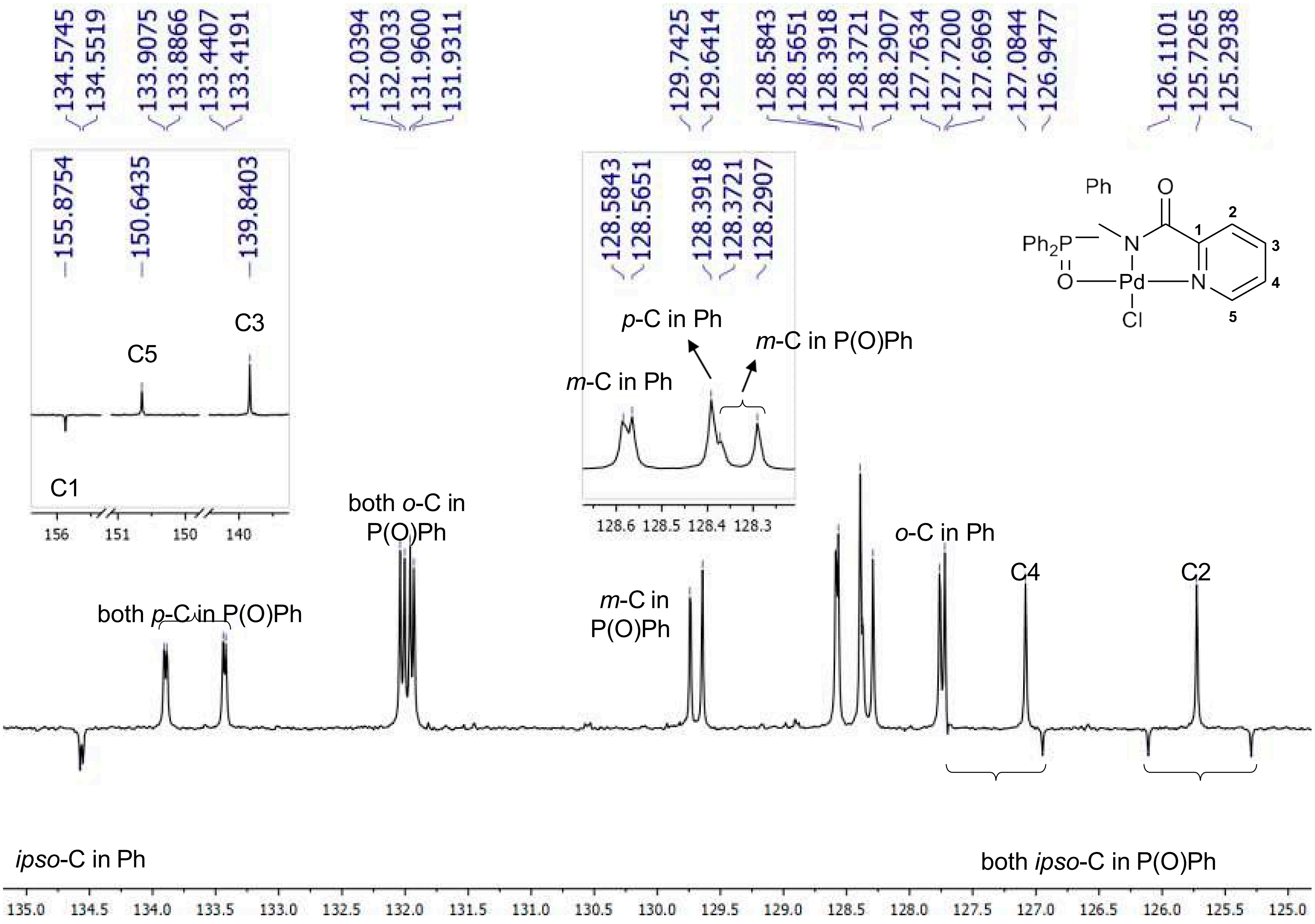

Figure S28. Extended fragments of the $^{13}C\{^{1}H\}$ NMR spectrum of complex **3b** (125.76 MHz, $CDCl_3$)

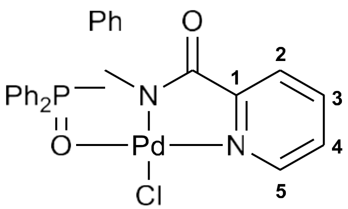

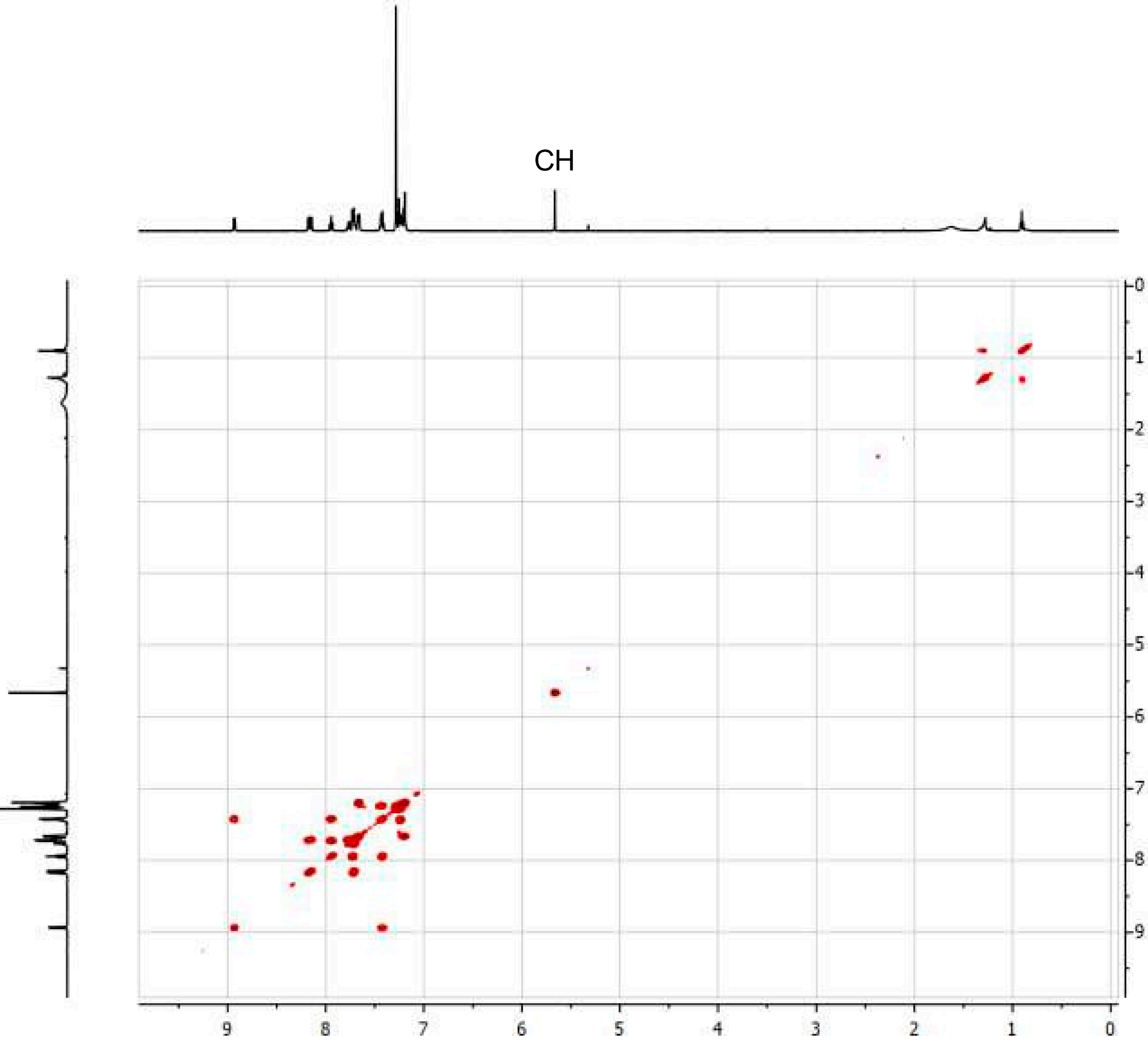

Figure S29. ^1H–^1H COSY spectrum of complex **3b** (500.13 MHz, $CDCl_3$)

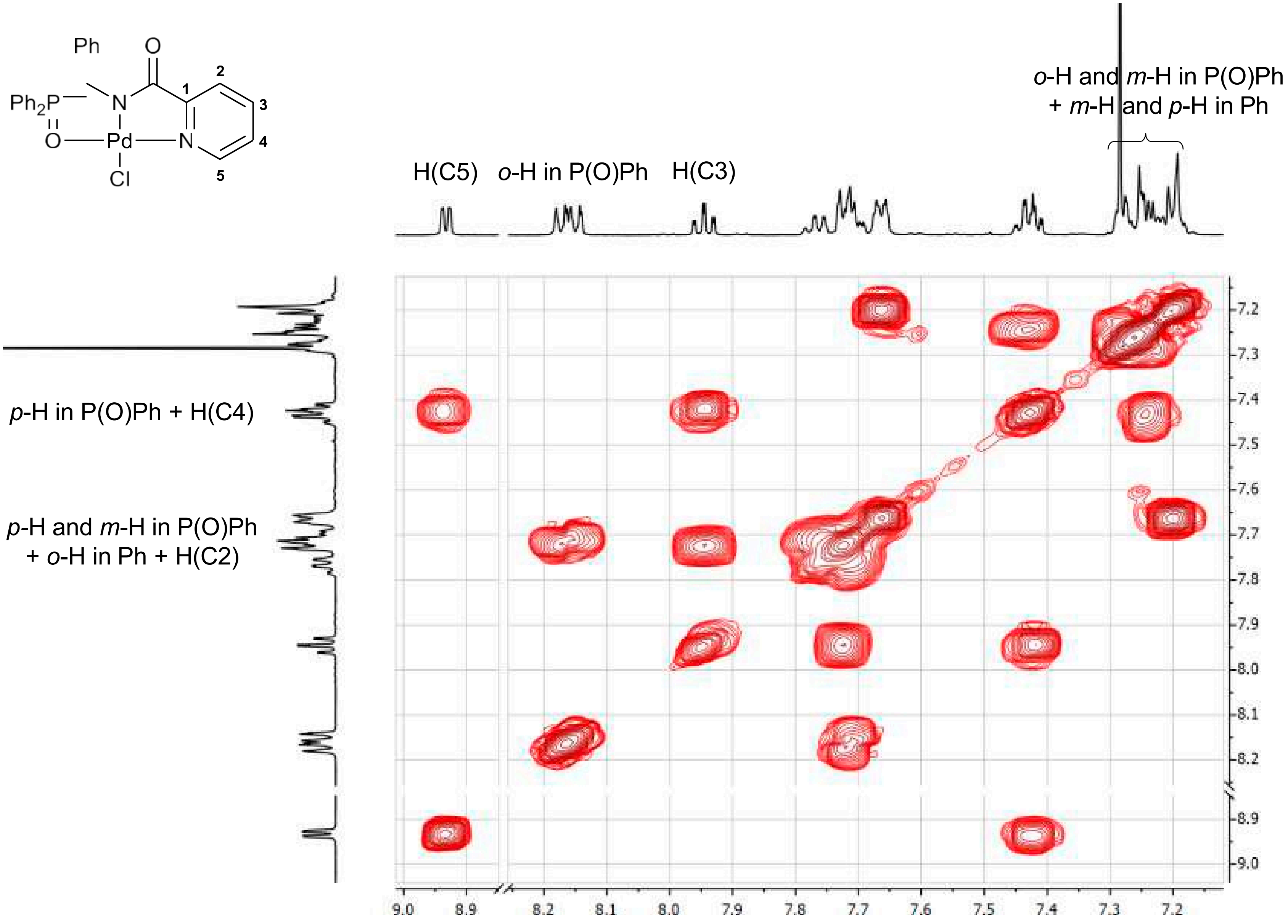

Figure S30. Extended fragments of the ^{1}H–^{1}H COSY spectrum of complex **3b** (500.13 MHz, $CDCl_3$.

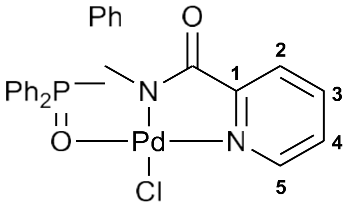

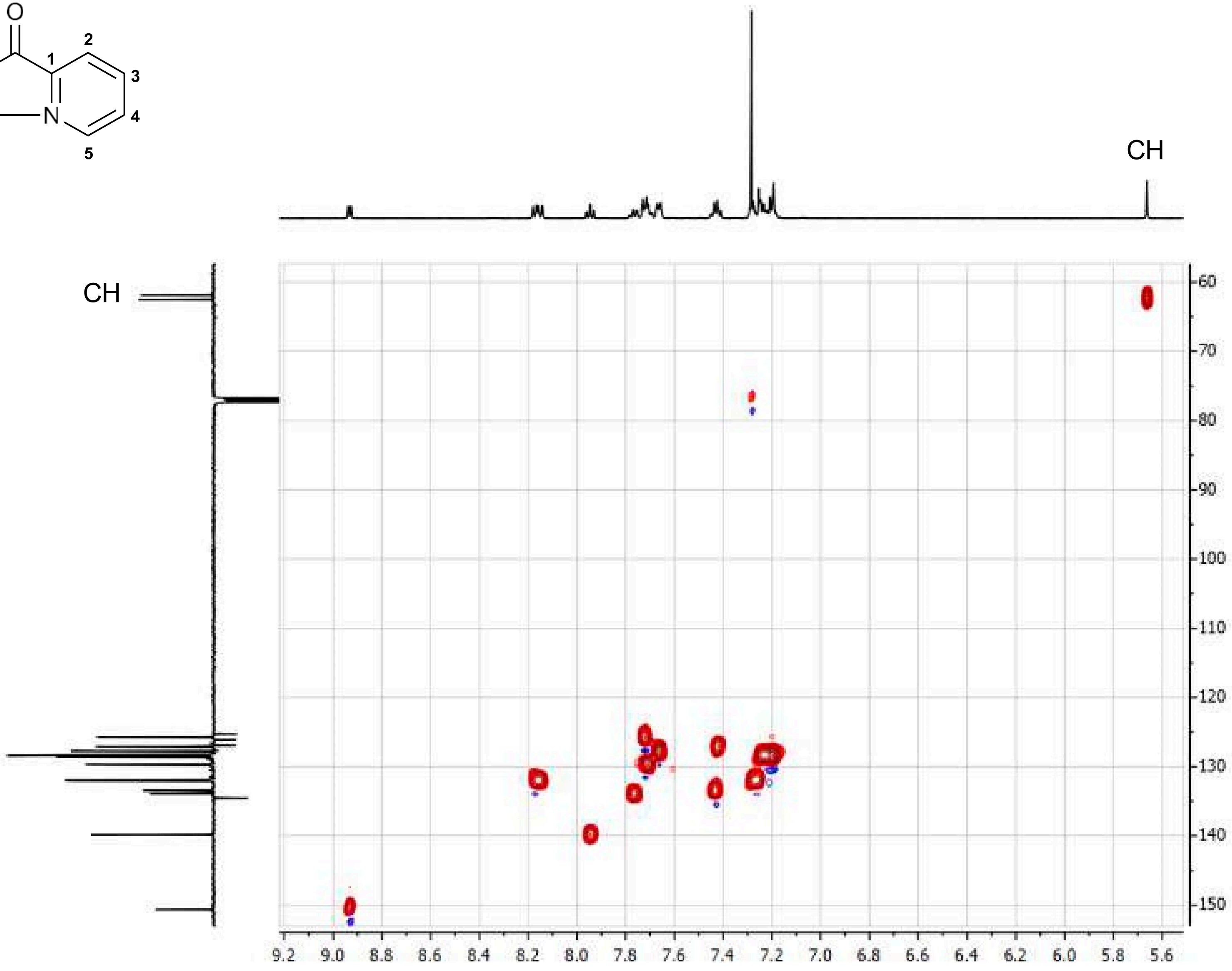

Figure S31. ^{1}H–^{13}C HSQC spectrum of complex **3b** ($CDCl_3$)

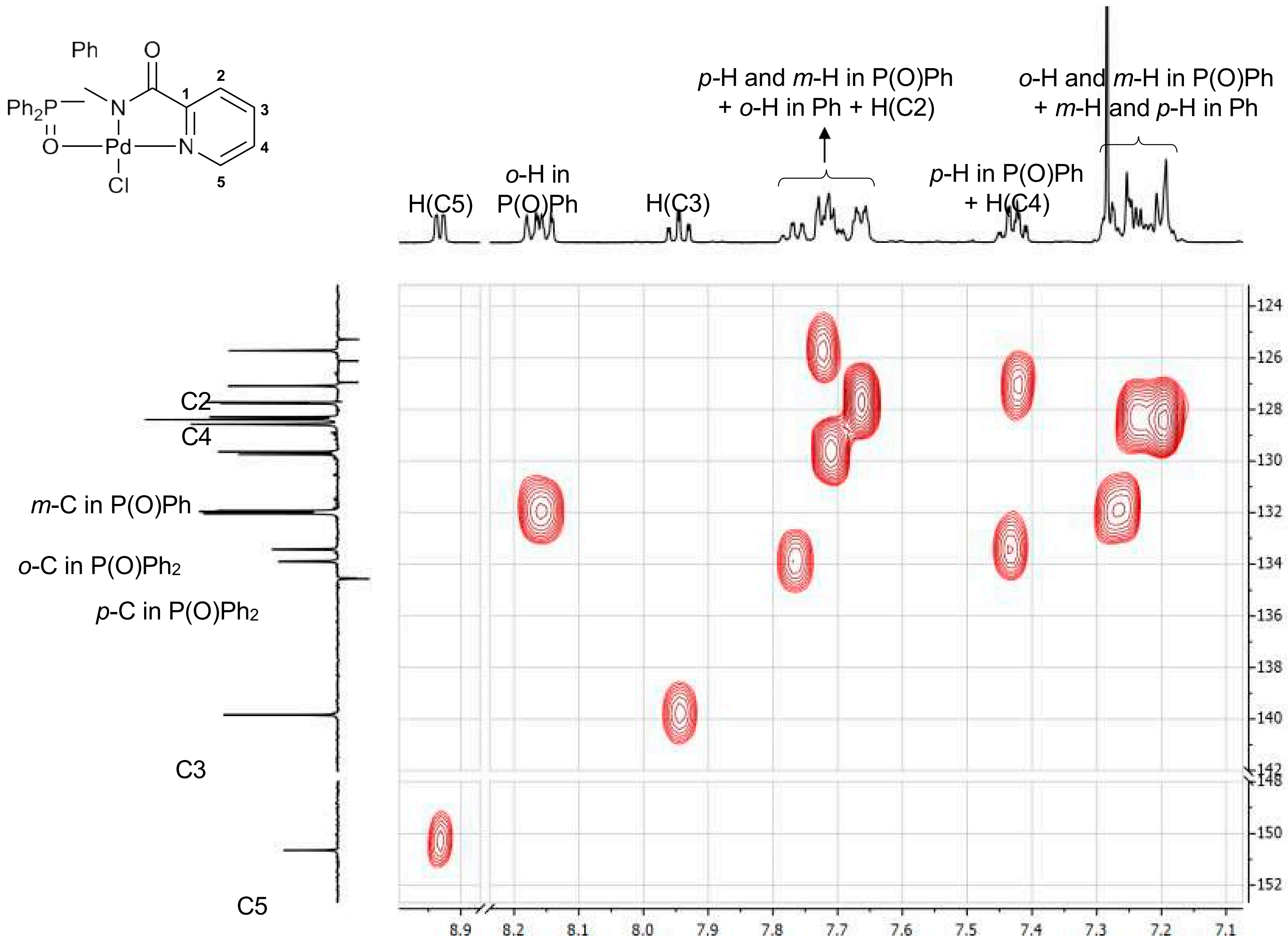
Ph
O
Ph_2P
N
O
Pd
N
Cl
1
2
3
4
5
p-H and m-H in P(O)Ph
+ o-H in Ph + H(C2)
o-H and m-H in P(O)Ph
+ m-H and p-H in Ph
H(C5)
o-H in
P(O)Ph
H(C3)
p-H in P(O)Ph
+ H(C4)
C2
C4
m-C in P(O)Ph
o-C in $P(O)Ph_2$
p-C in $P(O)Ph_2$
C3
C5
124
126
128
130
132
134
136
138
140
142
148
150
152
8.9
8.2
8.1
8.0
7.9
7.8
7.7
7.6
7.5
7.4
7.3
7.2
7.1

Figure S32. Extended fragments of the ^{1}H–^{13}C HSQC spectrum of complex **3b** ($CDCl_3$)

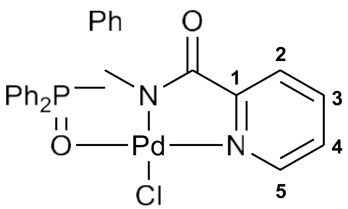

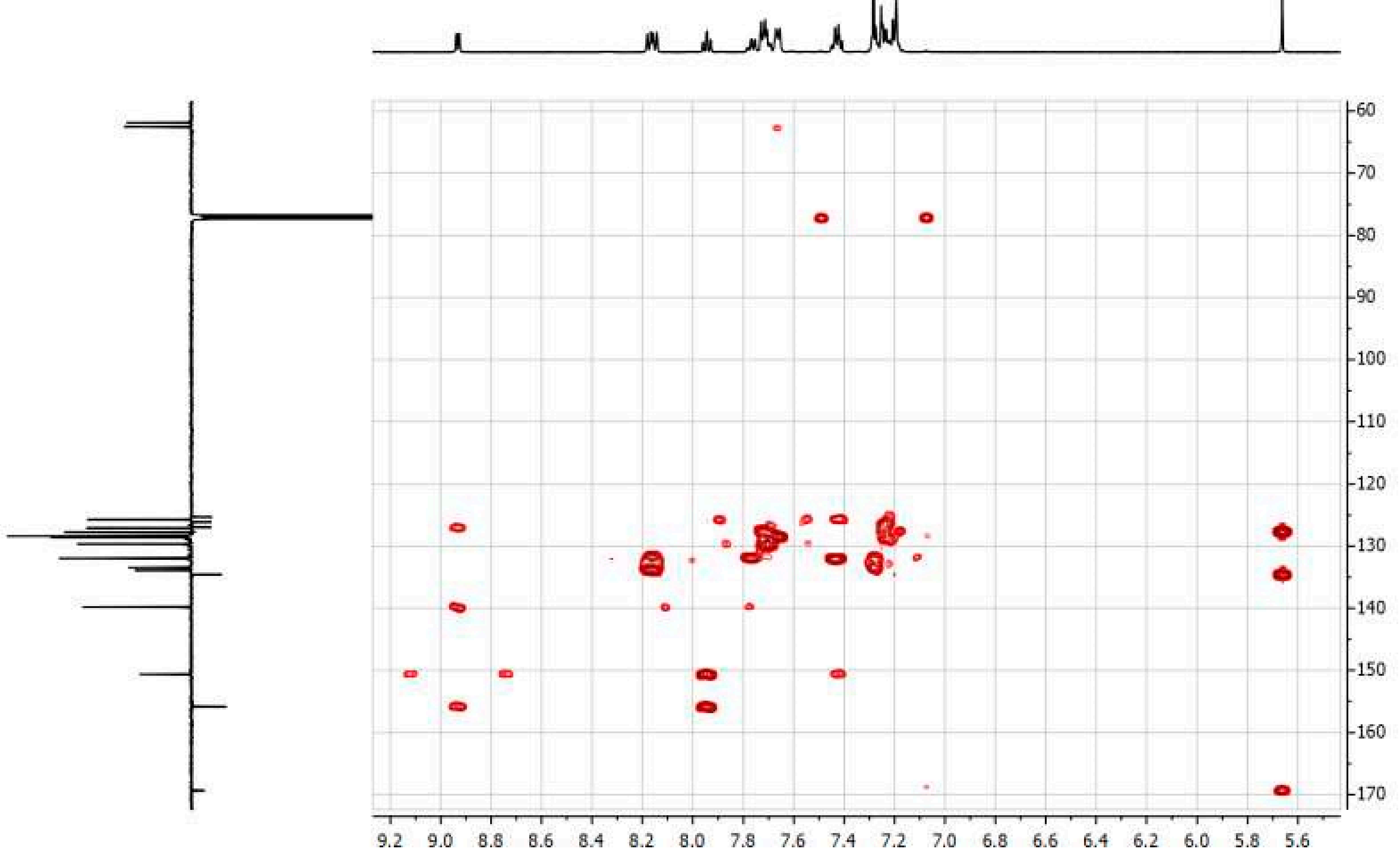

Figure S33. ^{1}H–^{13}C HMBC spectrum of complex **3b** ($CDCl_3$

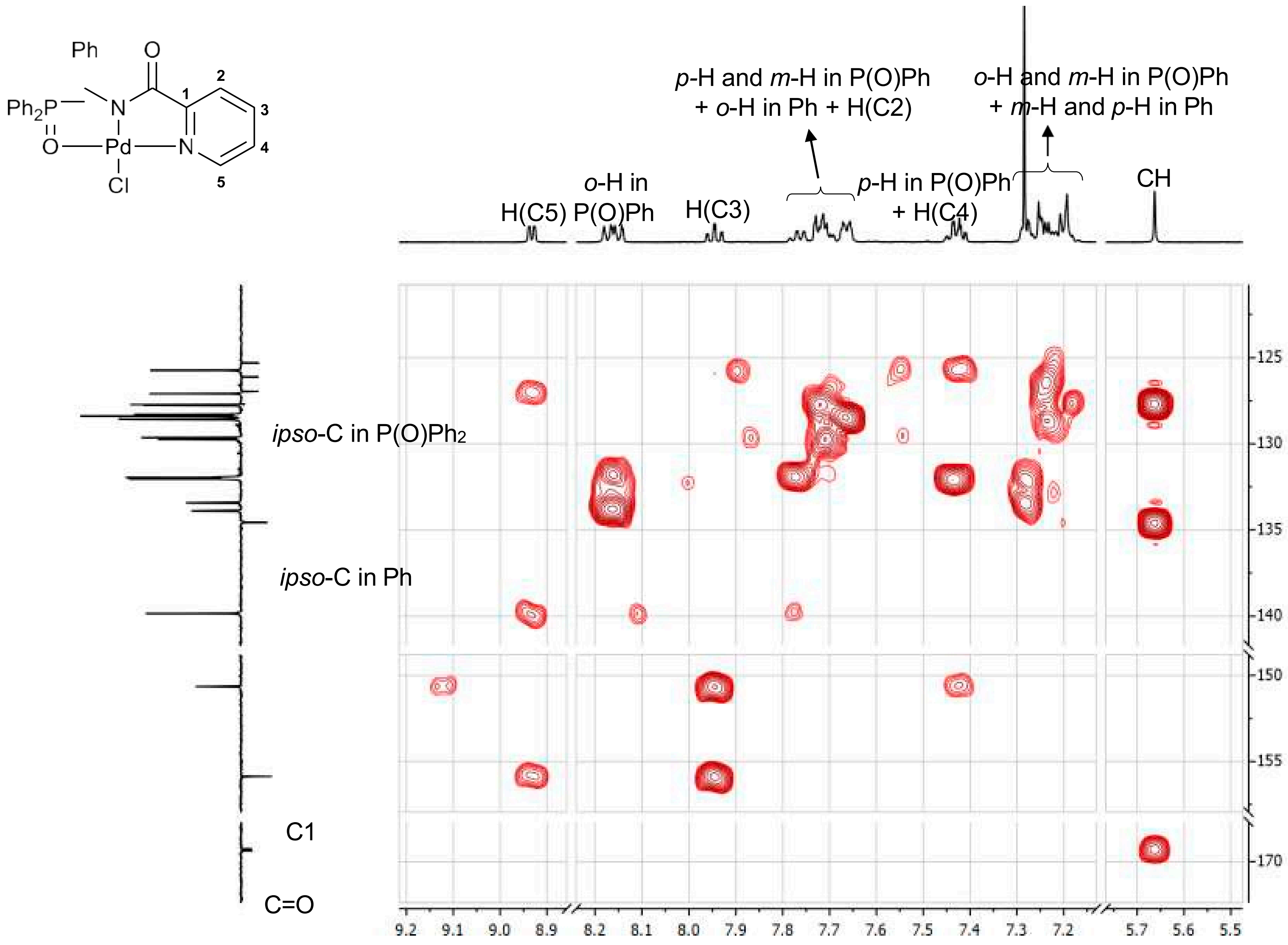
Ph
O
Ph2P
N
O
Pd
N
Cl
1
2
3
4
5
p-H and m-H in P(O)Ph
+ o-H in Ph + H(C2)
o-H and m-H in P(O)Ph
+ m-H and p-H in Ph
o-H in P(O)Ph
H(C5)
H(C3)
p-H in P(O)Ph
+ H(C4)
CH
ipso-C in P(O)Ph2
ipso-C in Ph
C1
C=O

Figure S34. Extended fragments of the ^{1}H–^{13}C HMBC spectrum of complex **3b** ($CDCl_3$)

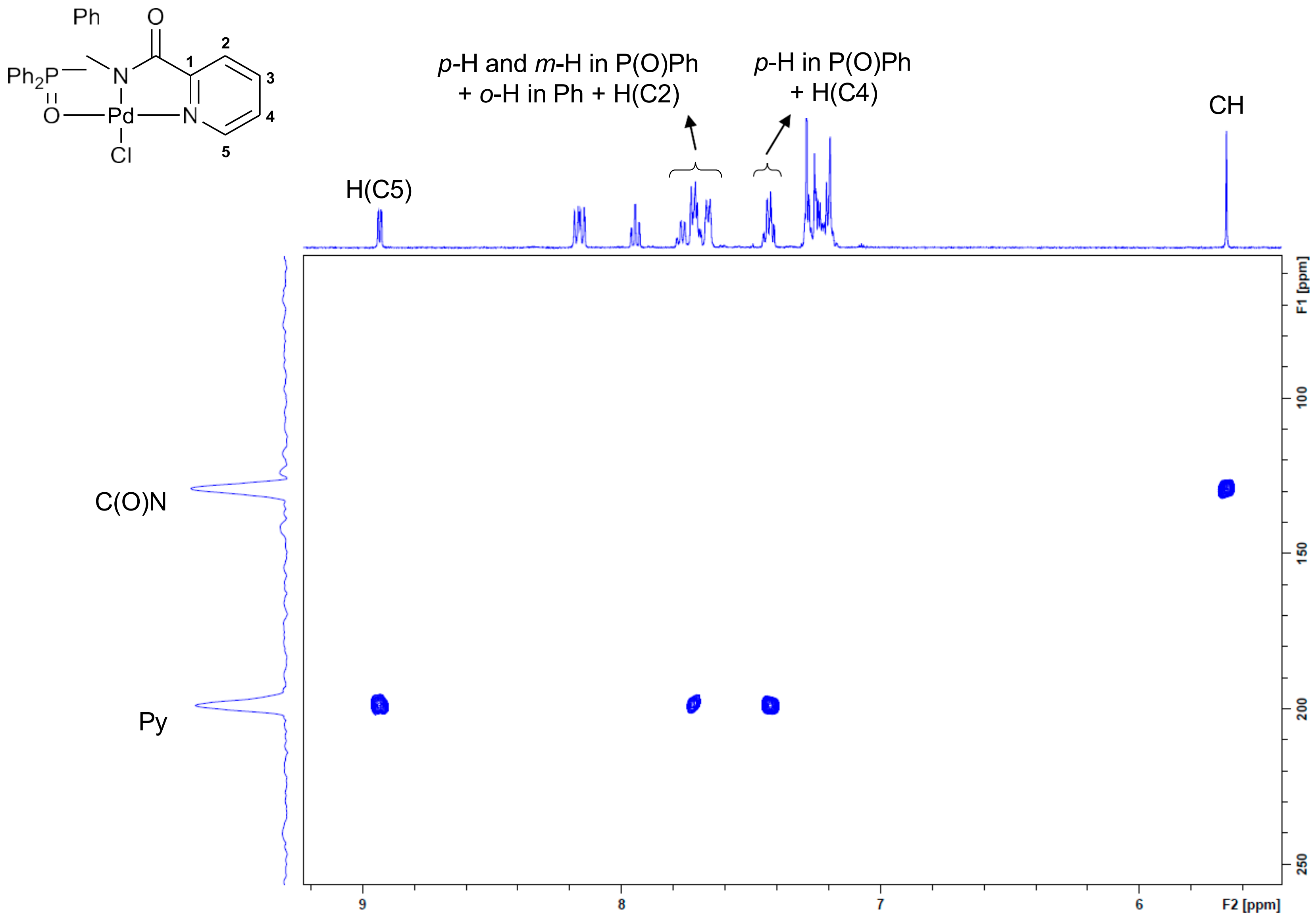

Figure S35. ^{1}H–^{15}N HMBC spectrum of complex **3b** ($CDCl_3$)

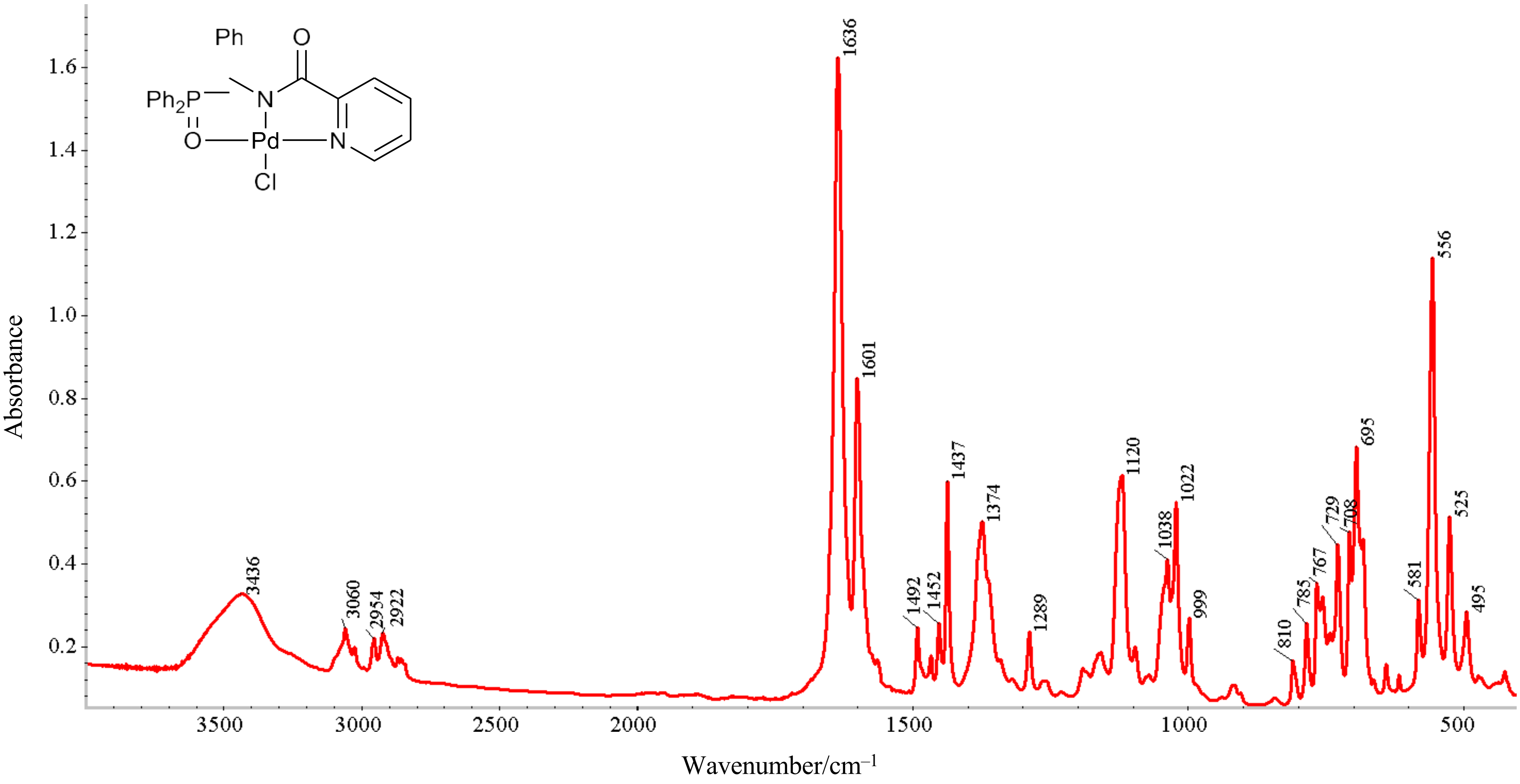

Figure S36. IR spectrum of complex **3b**

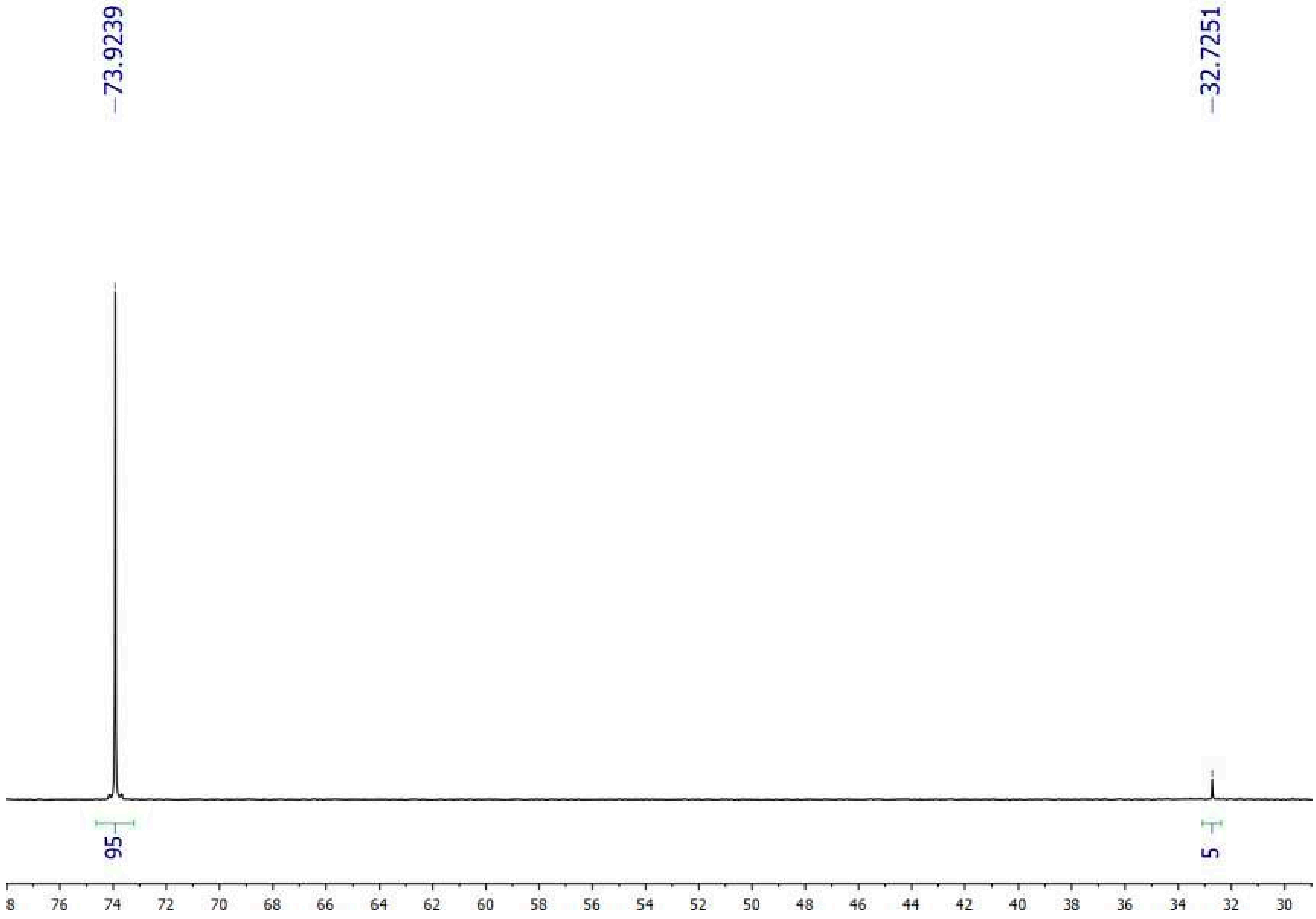

Figure S37. $^{31}P\{^{1}H\}$ NMR spectrum of a solution of complex **2b** in $CDCl_3$ (202.45 MHz)

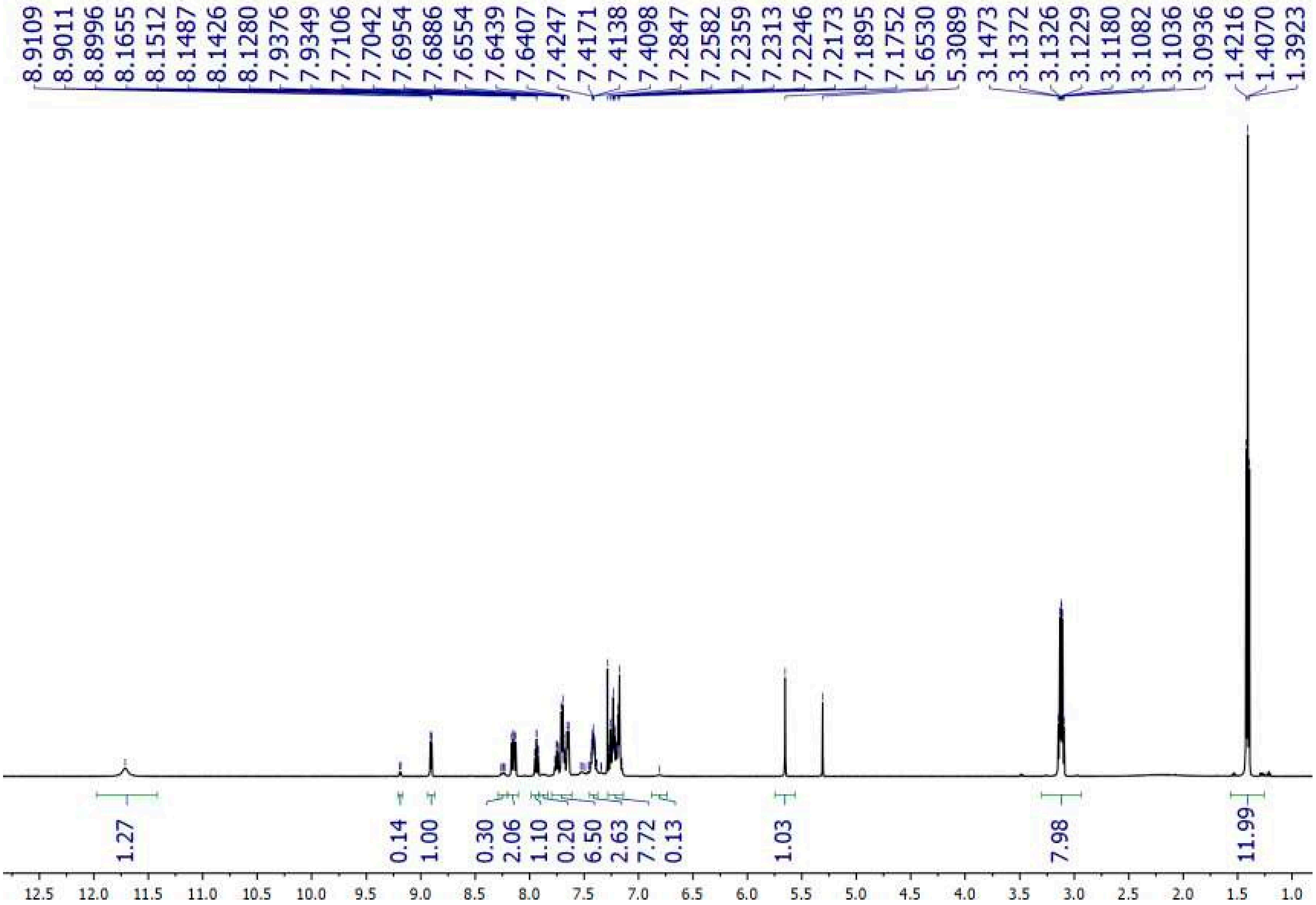

Figure S38. ^{1}H NMR spectrum of a solution of complex **2b** in $CDCl_3$ (500.13 MHz)

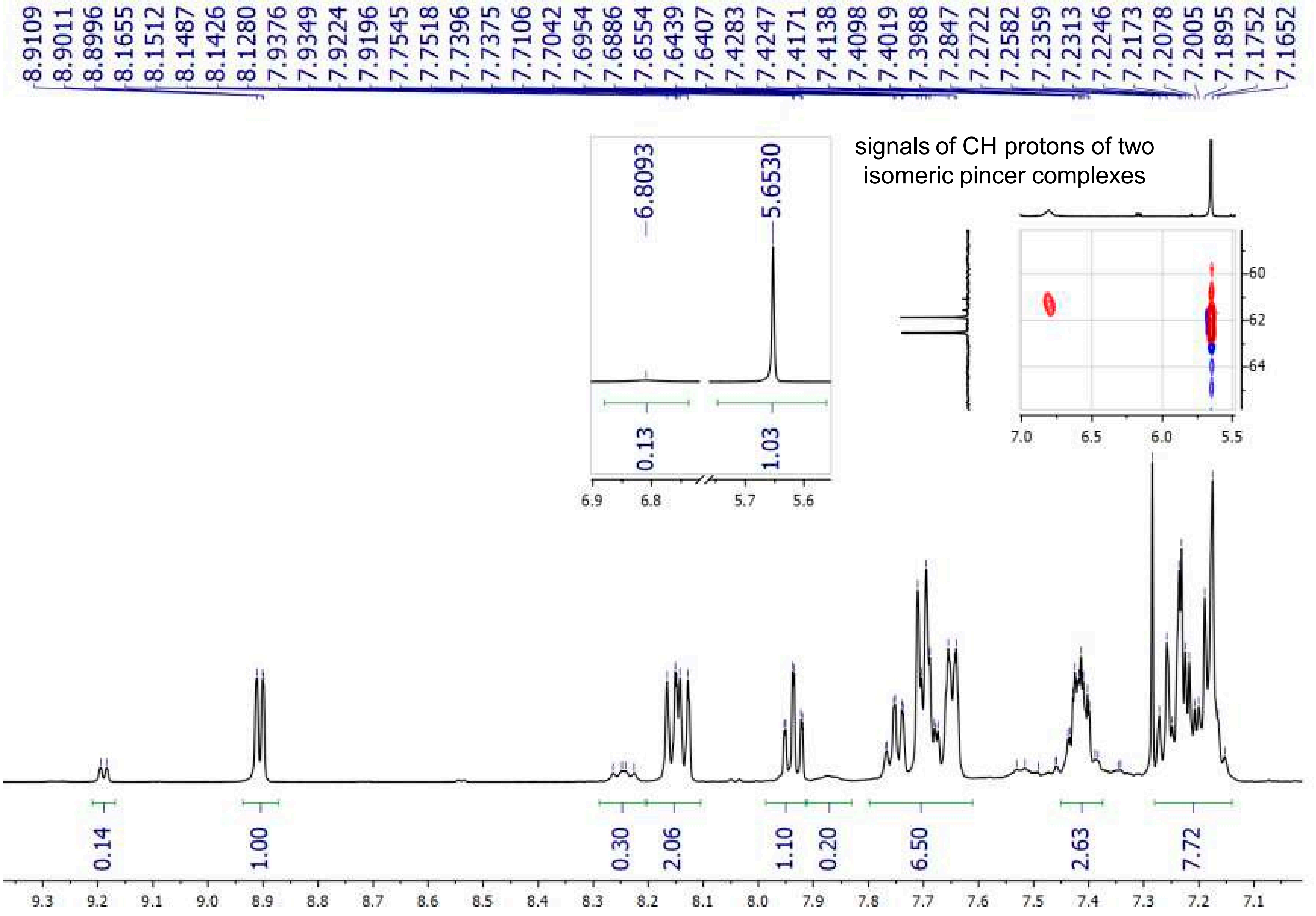

Figure S39. Extended fragments of the ^{1}H NMR and ^{1}H–^{13}C HSQC spectra of a solution of complex **2b** in $CDCl_3$

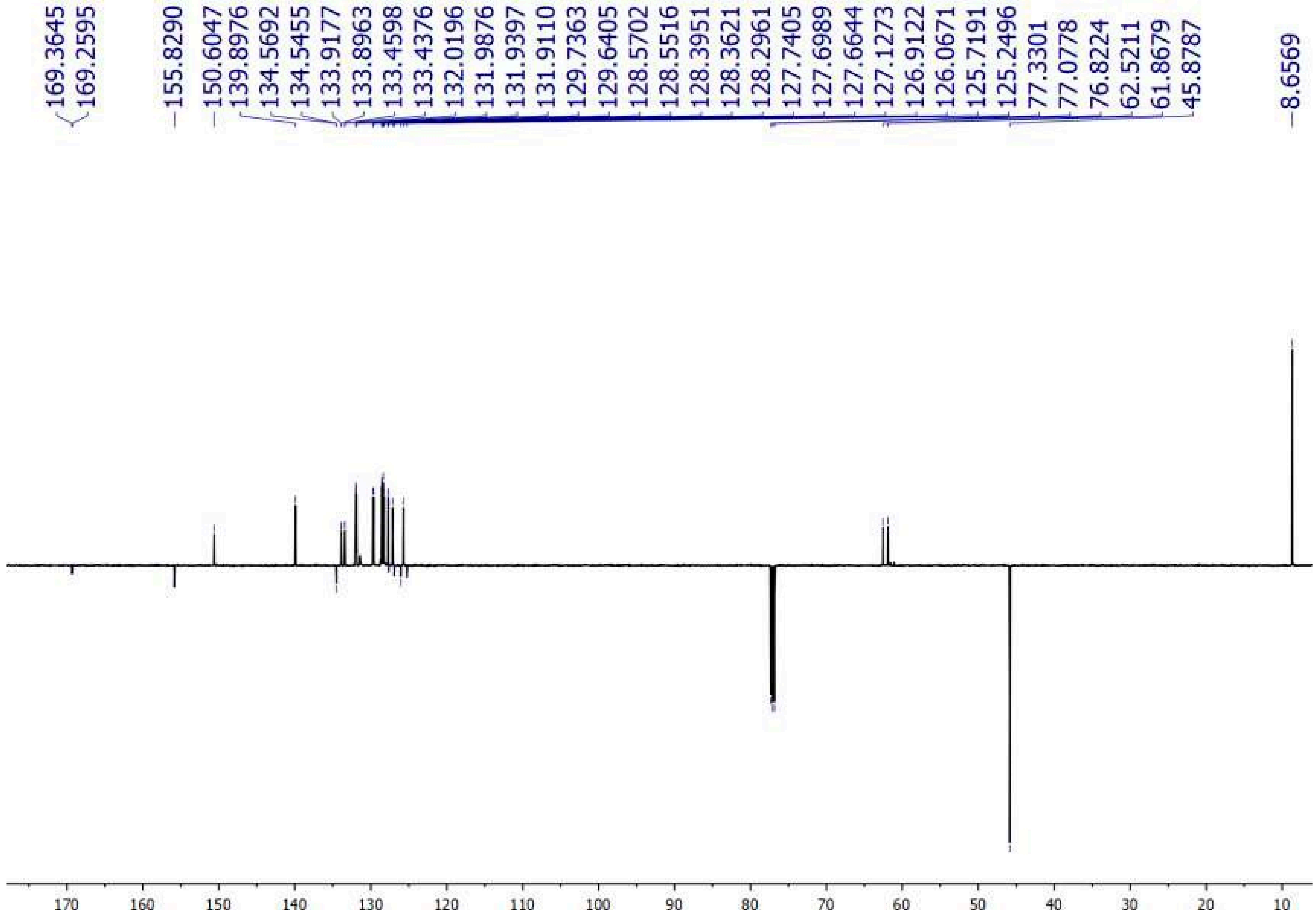

Figure S40. $^{13}C\{^{1}H\}$ NMR spectrum of a solution of complex **2b** in $CDCl_3$ (125.76 MHz)

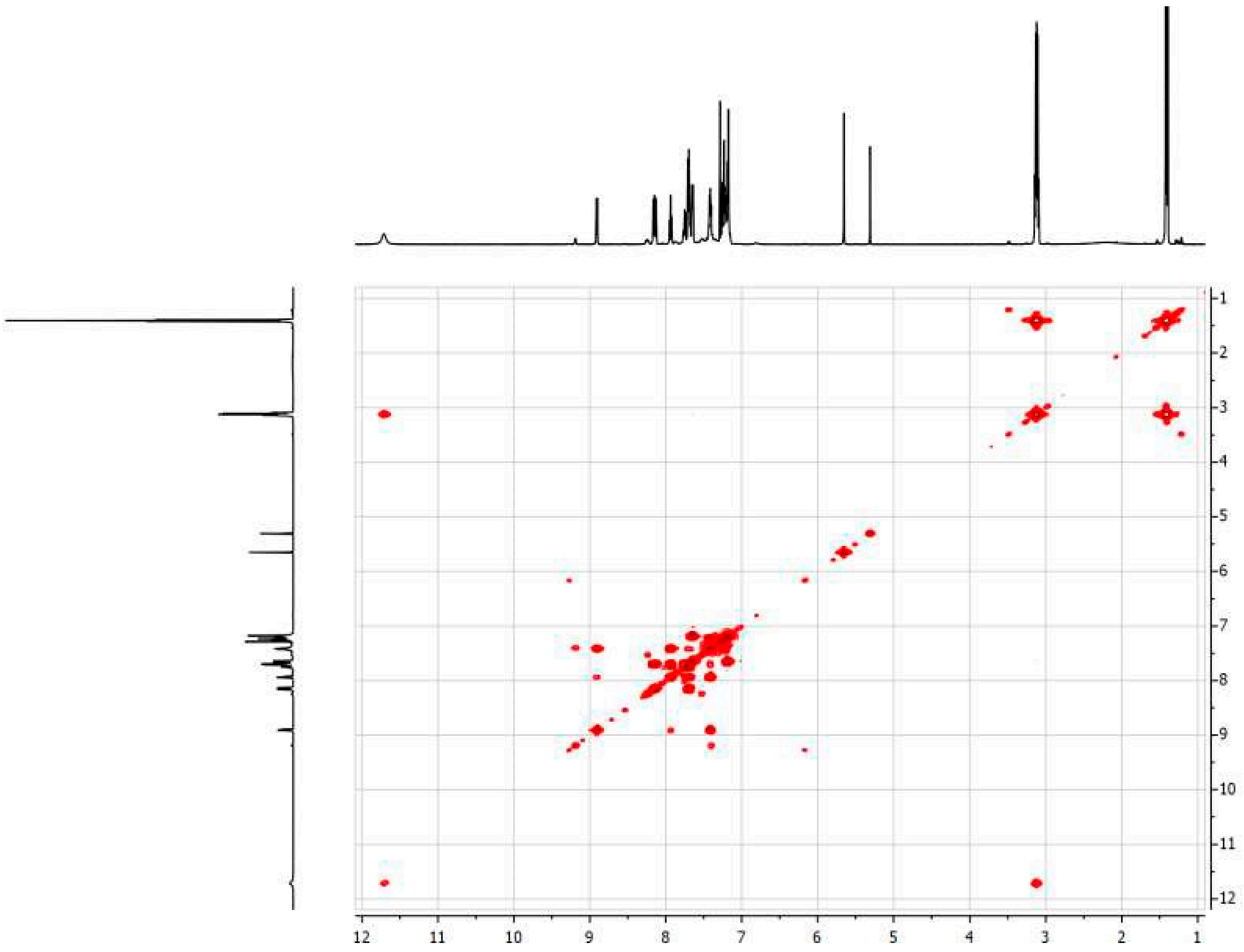

Figure S41. ^{1}H–^{1}H COSY spectrum of a solution of complex **2b** in $CDCl_3$

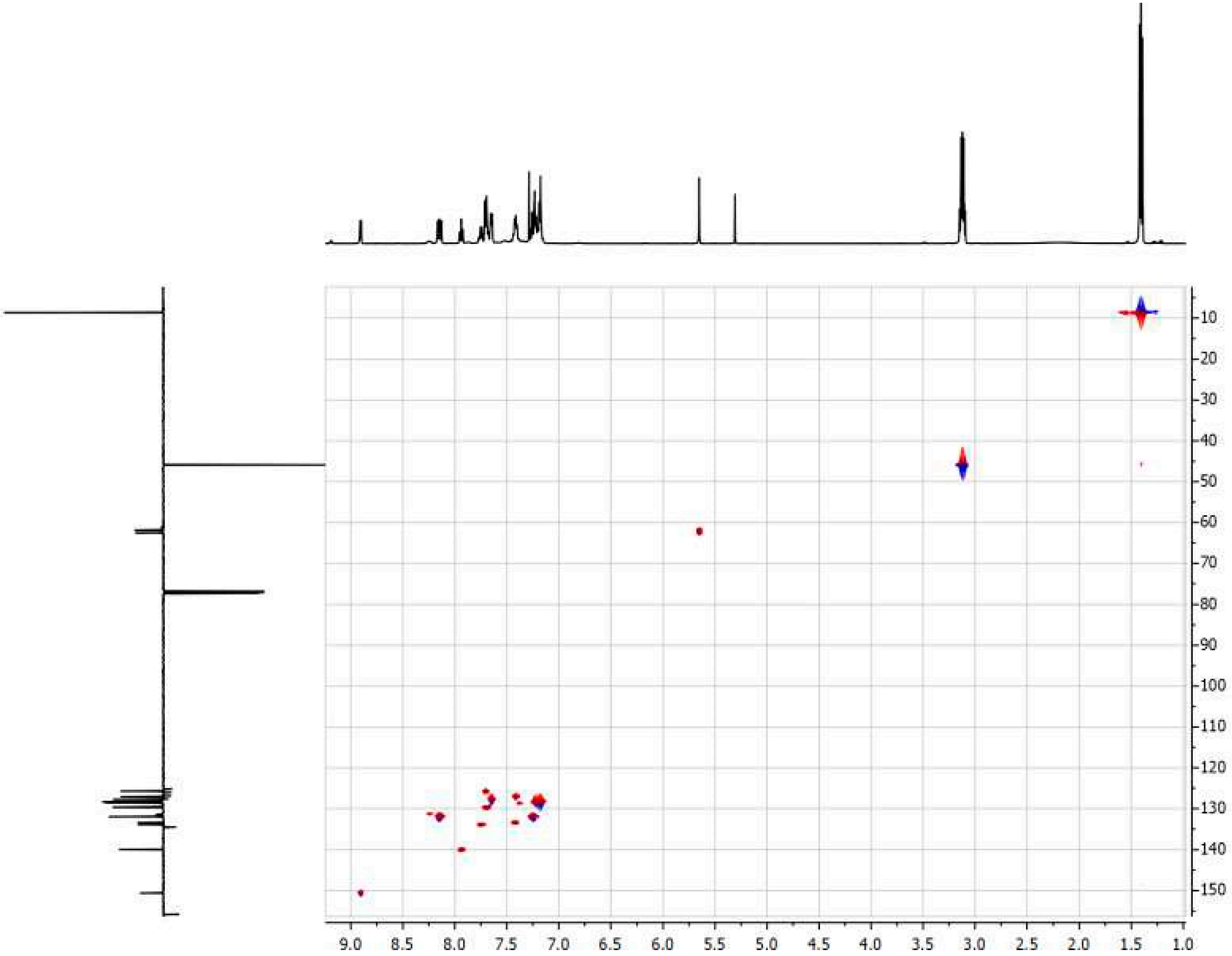

Figure S42. ^{1}H–^{13}C HSQC spectrum of a solution of complex **2b** in $CDCl_3$

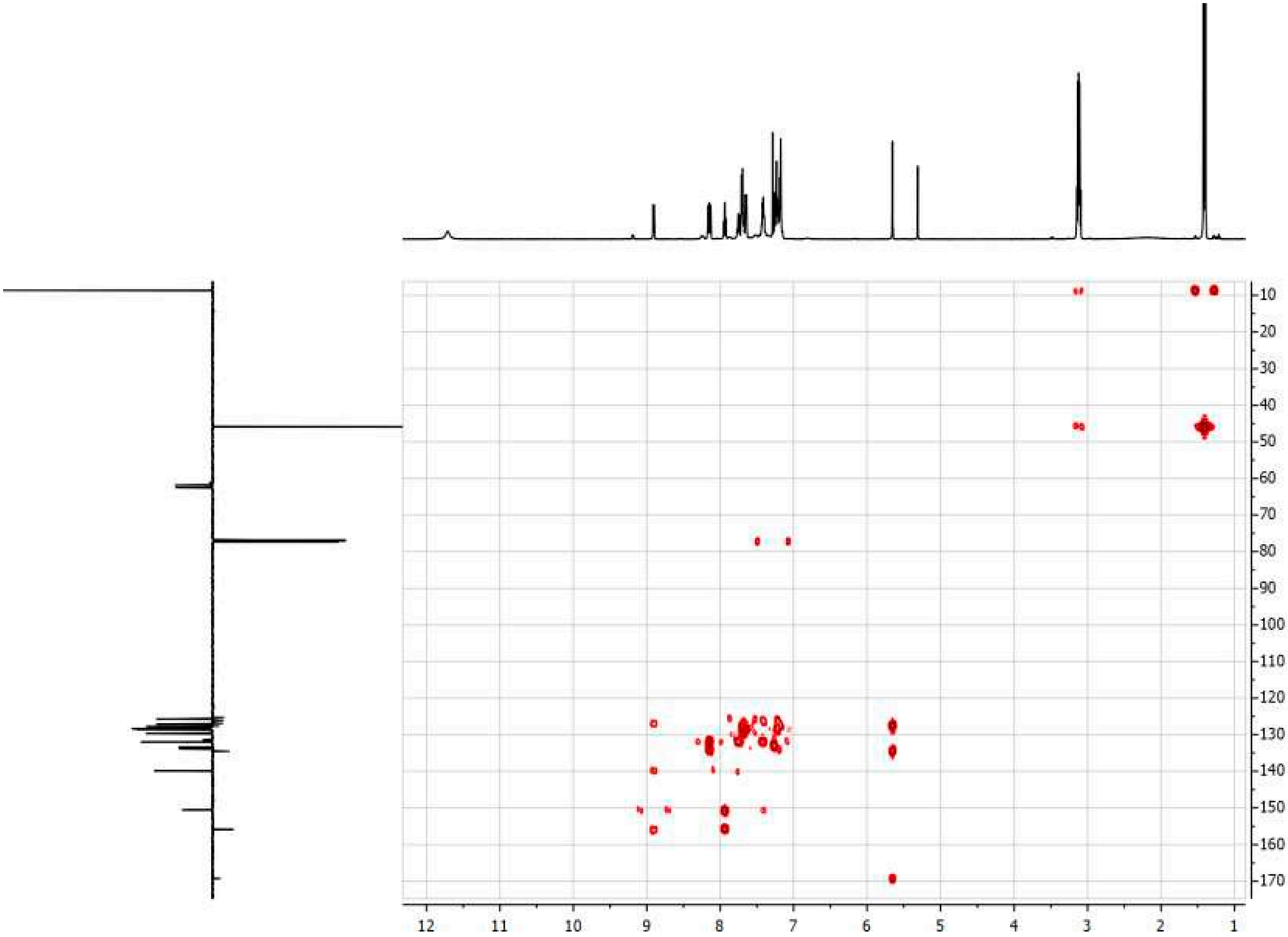

Figure S43. ^{1}H–^{13}C HMBC spectrum of a solution of complex **2b** in $CDCl_3$

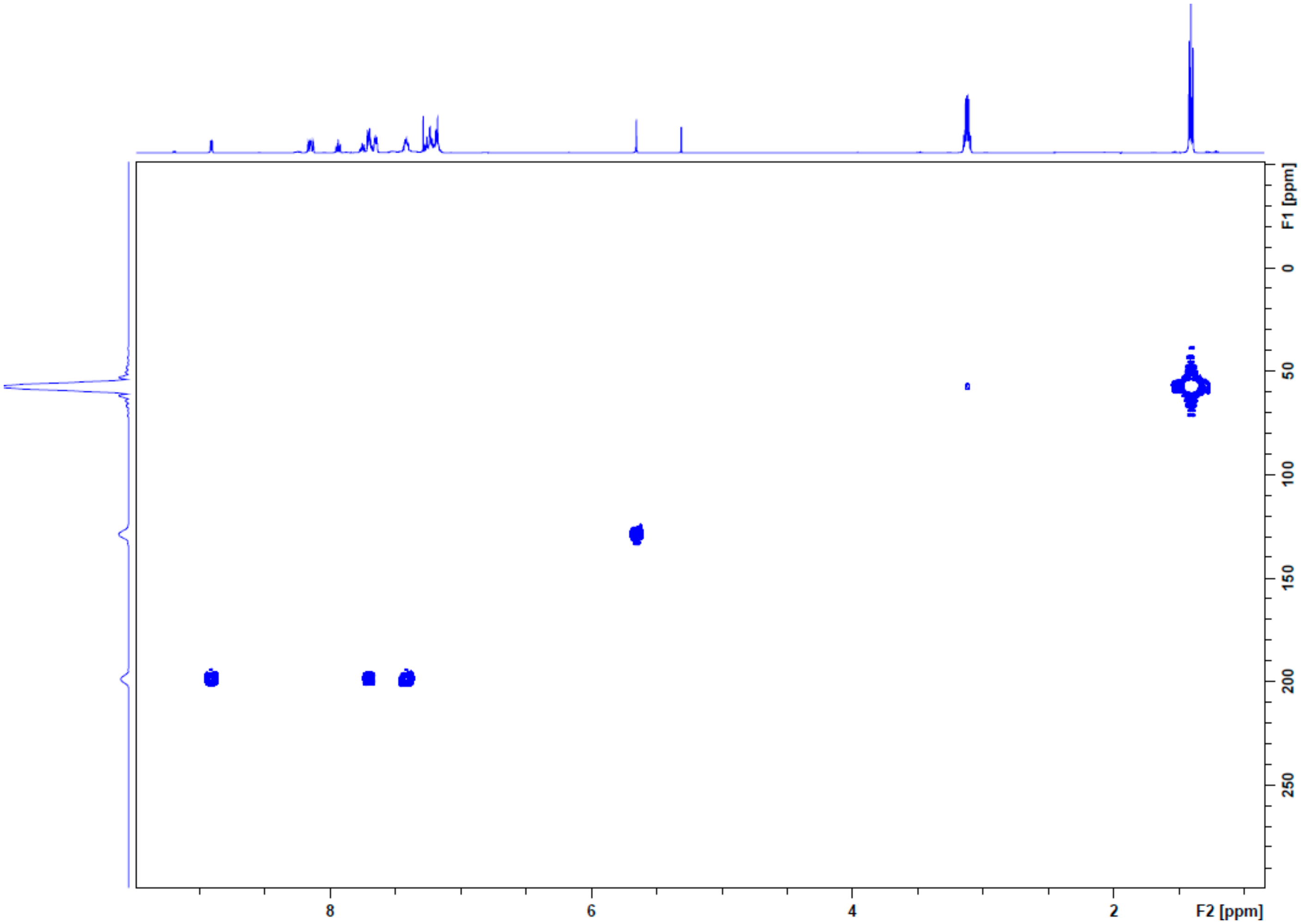

Figure S44. ^{1}H–^{15}N HMBC spectrum of a solution of complex **2b** in $CDCl_3$

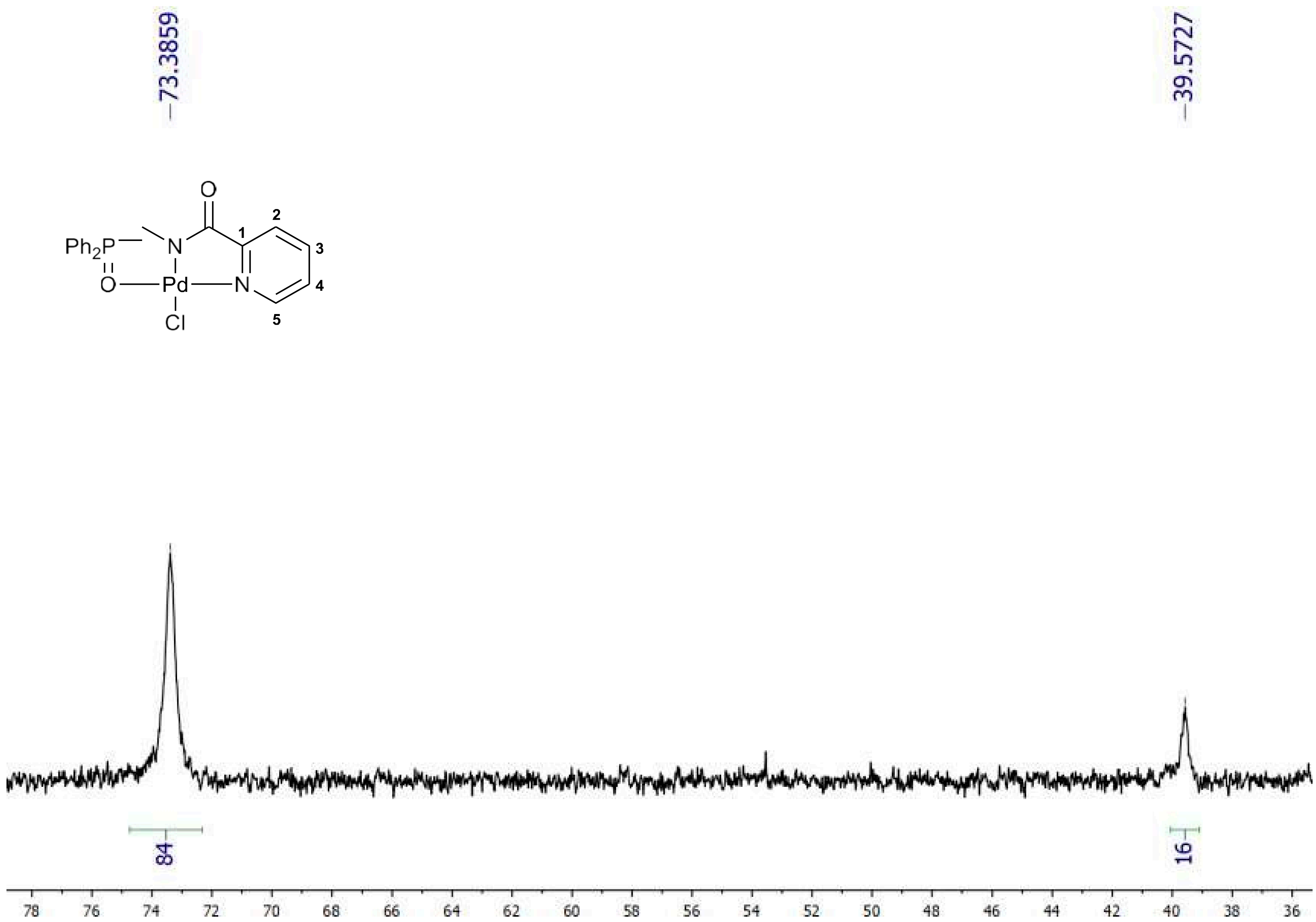

Figure S45. $^{31}P\{^1H\}$ NMR spectrum of complex **3a** (161.98 MHz, $CDCl_3$)

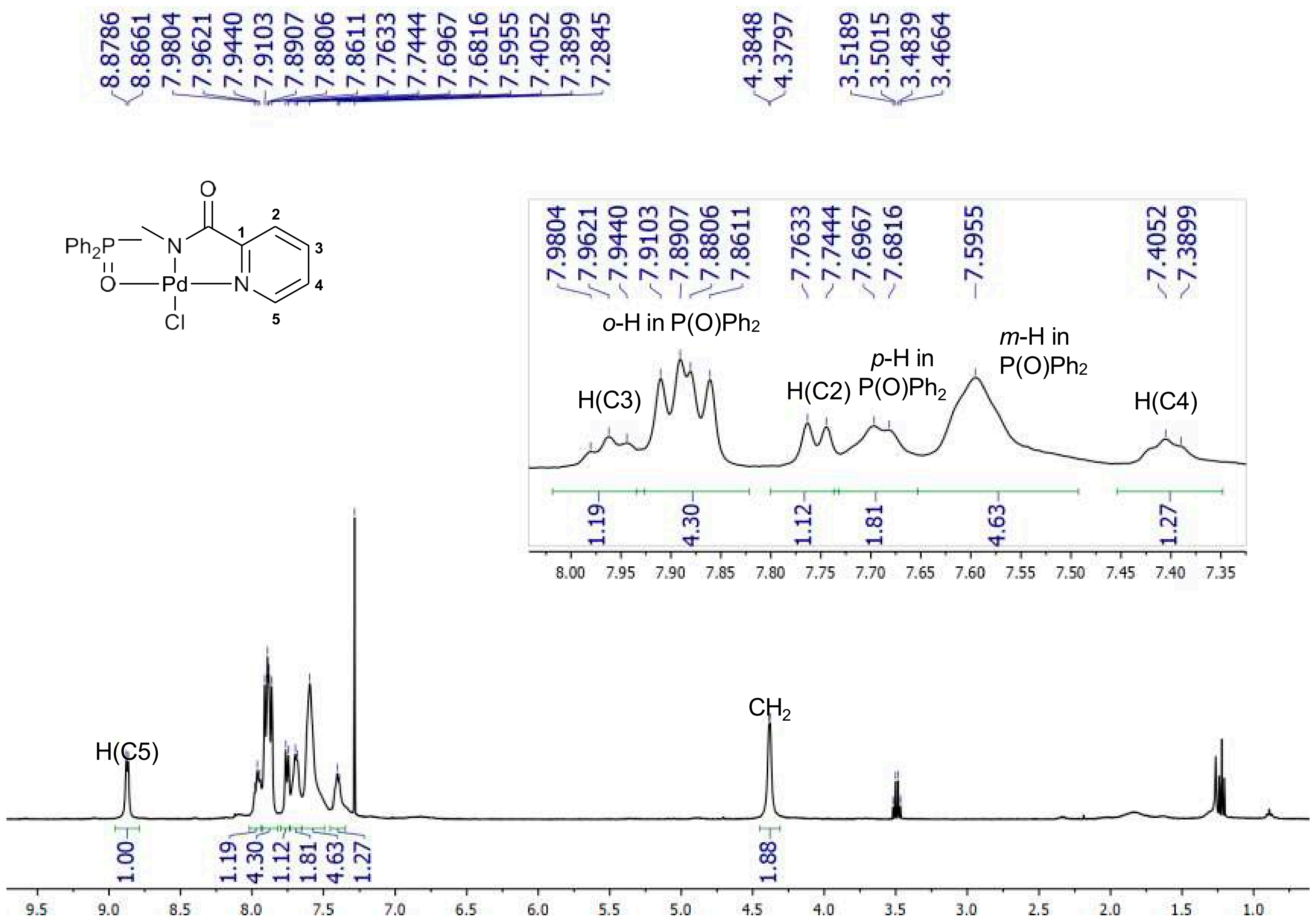

Figure S46. ^{1}H NMR spectrum of complex **3a** (400.13 MHz, $CDCl_3$)

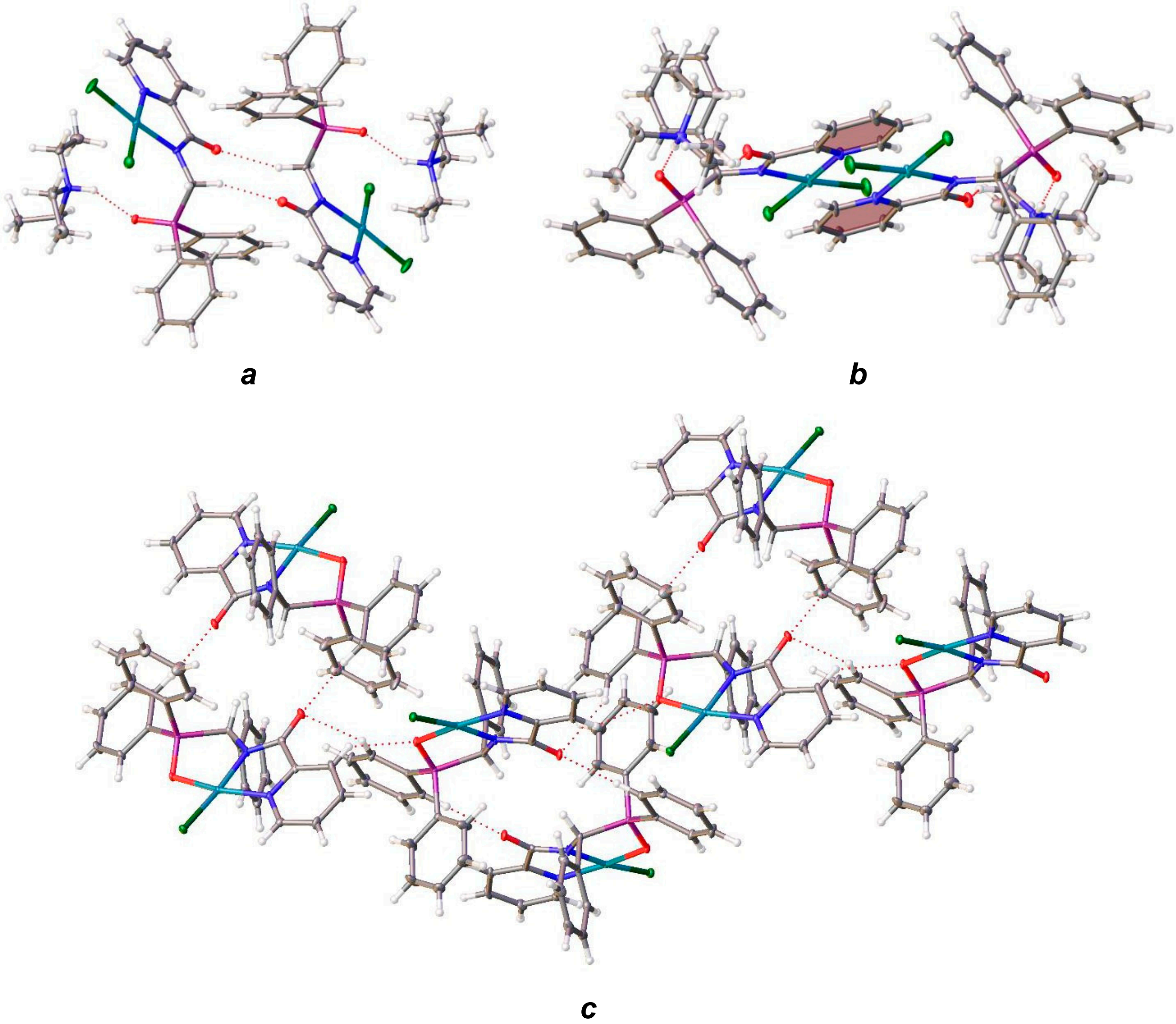

Figure S47. Fragments of the crystal packing of complexes **2a** (*a*), **2b** (*b*) and **3b** (*c*)

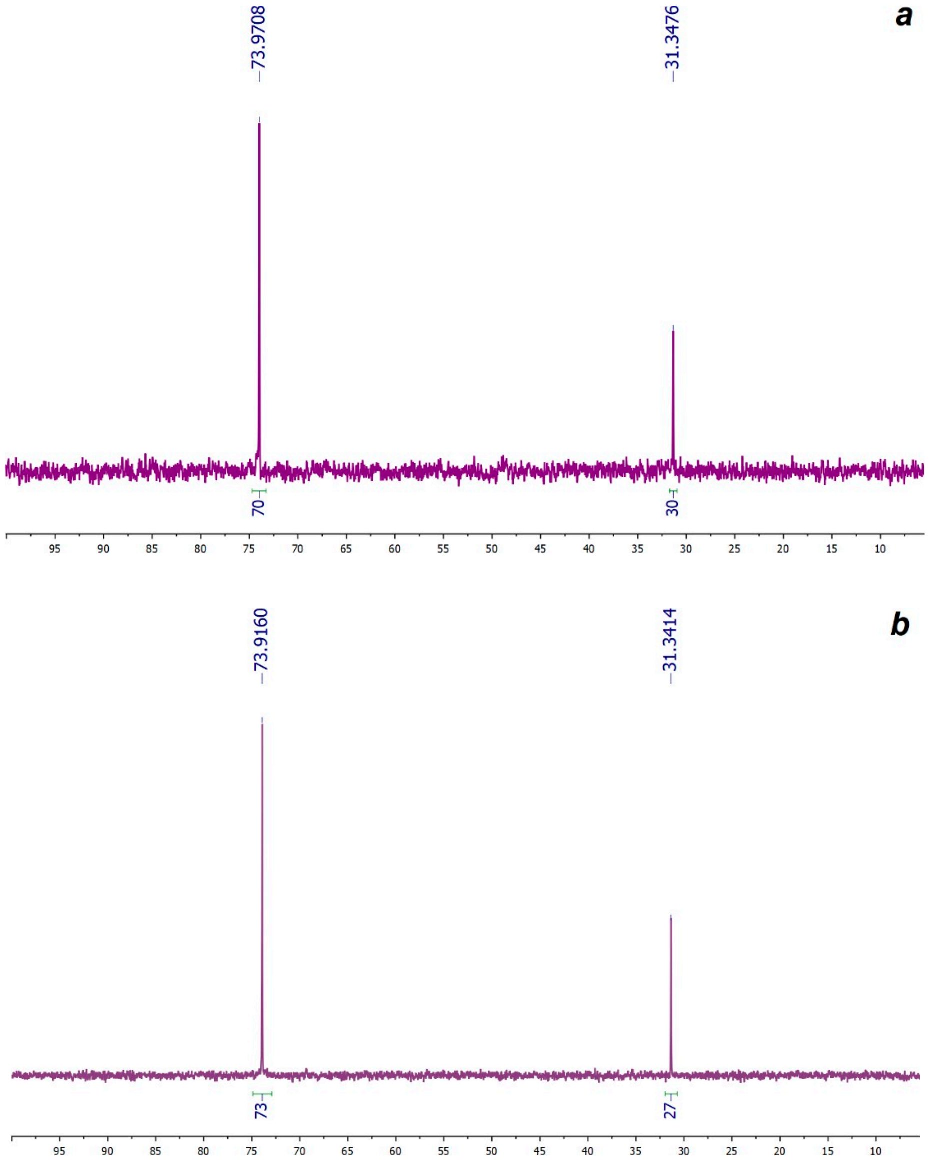

Figure S48. $^{31}P\{^1H\}$ NMR spectra of complex **2b** in $CDCl_3$–$(CD_3)_2SO$ registered in 3 h (***a***) and 3 days (***b***) after dissolution.

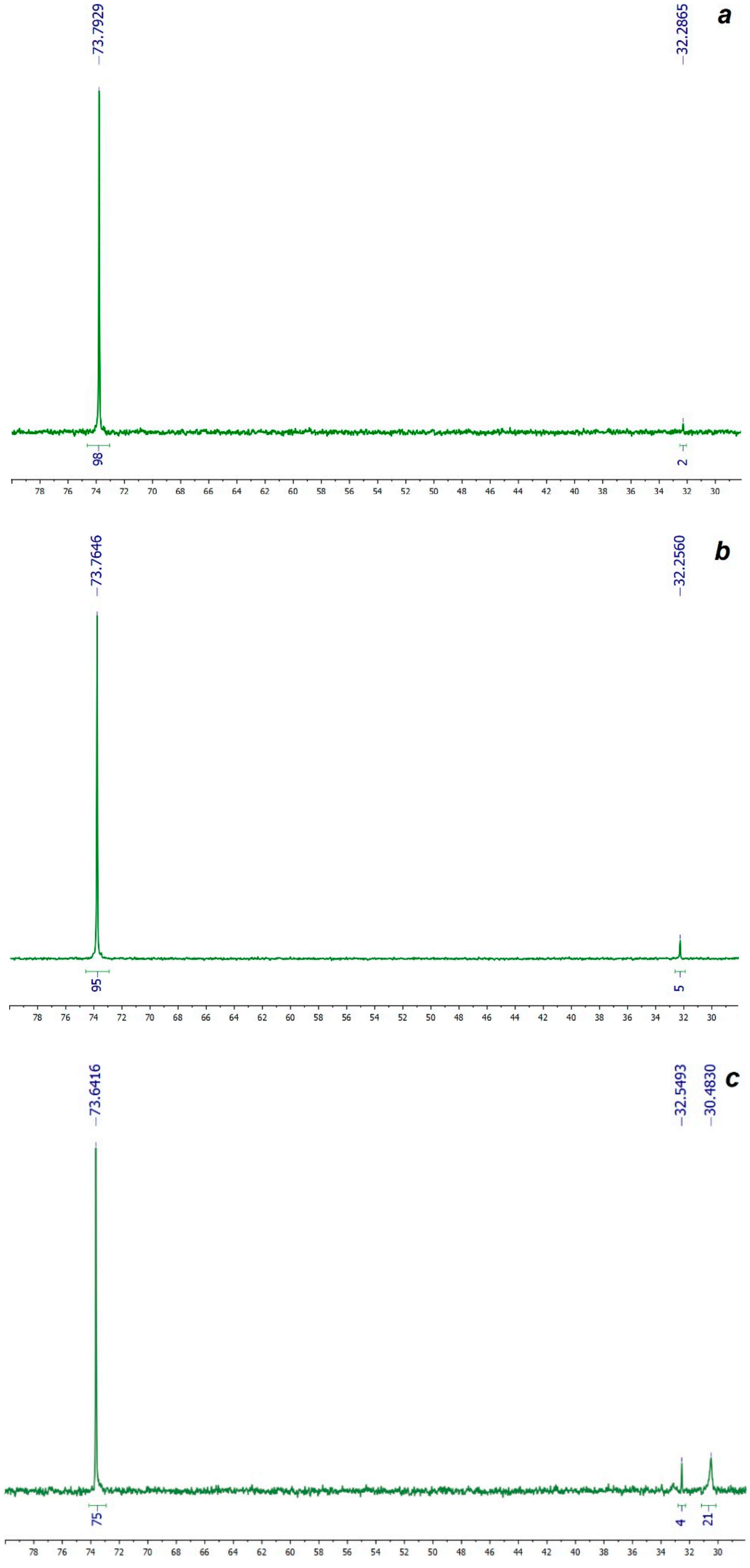

Figure. S49. $^{31}P\{^{1}H\}$ NMR spectra of complex **3b** in CDCl3–(CD3)2SO registered in 1 h (***a***), 8 h (***b***), and 1 week (***c***) after dissolution

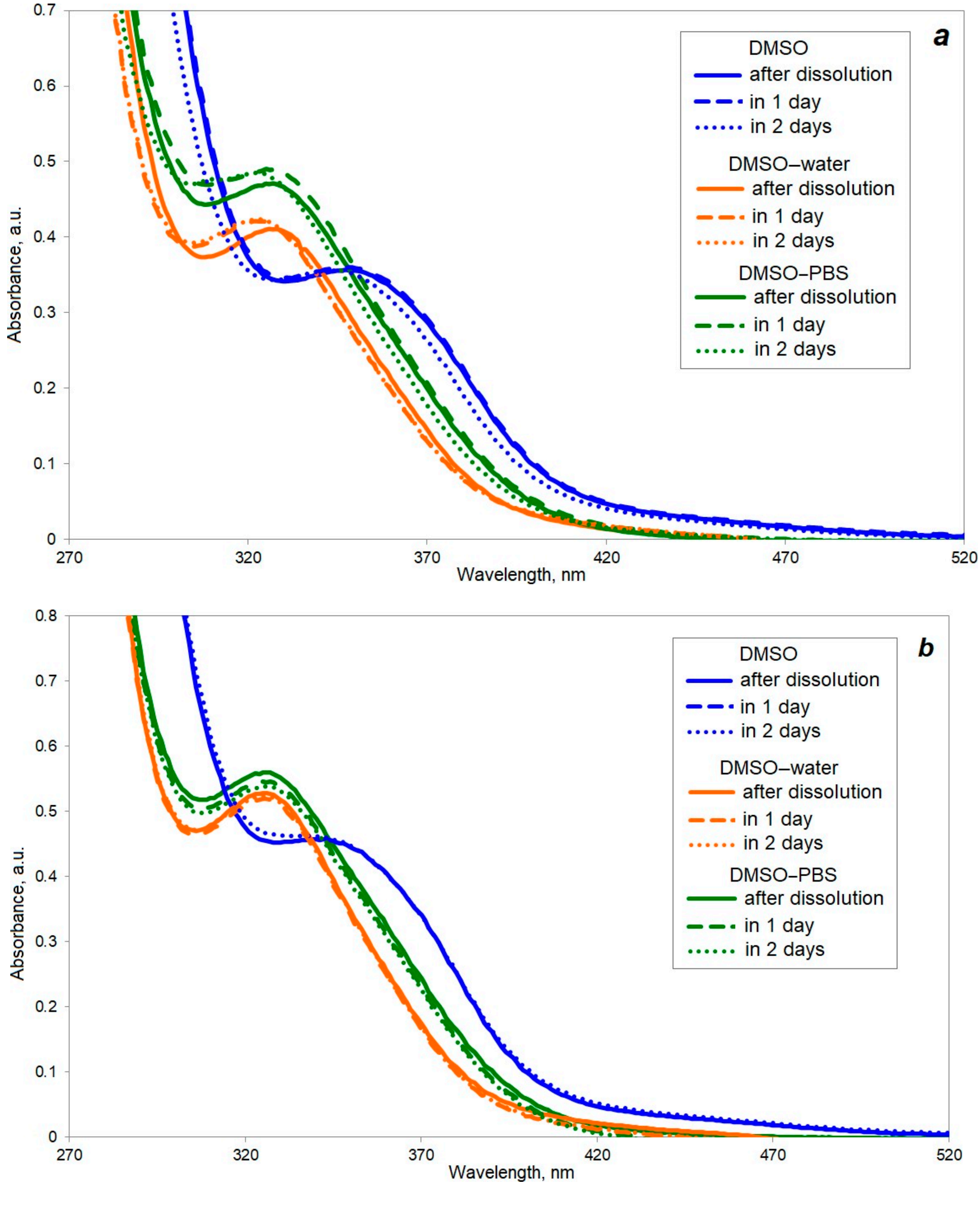

Figure. S50. UV-vis spectra of complexes **2a** (***a***) and **3a** (***b***) in DMSO, DMSO–deionized water (1:1), and DMSO–PBS (1:1) registered immediately after dissolution, in one or two days

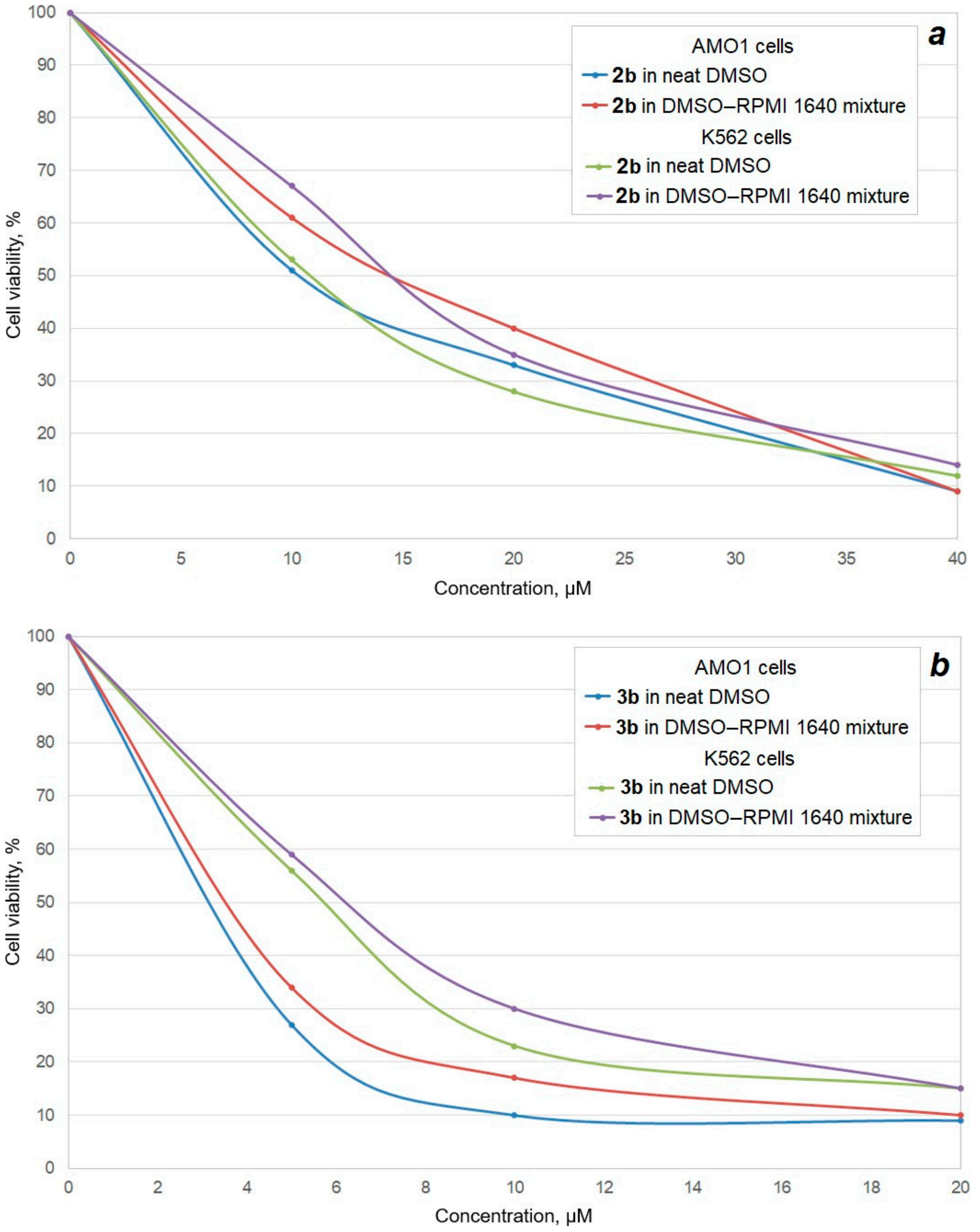

Figure S51. Effect of complexes **2b** (***a***) and **3b** (***b***) dissolved either in neat DMSO or DMSO–RPMI 1640 mixture on AMO1 and K562 cells (cell viability was defined by the conventional MTT assay according to the methodology described in the Experimental section with slight modifications: the complexes explored were preliminarily kept in neat DMSO or DMSO–RPMI 1640 mixture for 2 days before the experiments and the following incubation period was 72 h)

Table S1. Selected bond lengths (Å) and angles (°) for complexes **5** and **7**.

**5**			
Pd1–Cl1	2.3063(13)	C10–N2	1.331(5)
Pd1–O2	2.084(3)	Cl1–Pd1–N2	171.72(10)
Pd1–N1	2.038(3)	O2–Pd1–N1	166.40(11)
Pd1–N2	1.971(3)	N1–Pd1–N2	80.92(13)
P1–O2	1.523(3)	N2–Pd1–O2	85.66(12)
N1–C1	1.339(5)	O2–Pd1–Cl1	90.57(8)
C1–C10	1.509(5)	Cl1–Pd1–N1	102.44(10)
C10–O1	1.240(5)		
**7**[a]			
Pd1–Cl1	2.3317(17)	C10–O1	1.253(9)
Pd1–O2	2.058(5)	C10–N2	1.343(9)
Pd1–N1	1.995(6)	Cl1–Pd1–N2	171.24(16)
Pd1–N2	2.006(6)	O2–Pd1–N1	177.9(2)
P1–O2	1.530(5)	N1–Pd1–N2	91.0(2)
N1–C9	1.386(9)	N2–Pd1–O2	88.4(2)
C9–C1	1.425(10)	O2–Pd1–Cl1	86.27(14)
C1–C10	1.515(9)	Cl1–Pd1–N1	94.57(17)

[a] the geometric parameters of two symmetry-independent molecules are very close; here are the selected bond lengths and angles for the molecule presented in Figure 4.

Table S2. Crystal data and structure refinement parameters for compounds **1b**, **2a**, **2b**, **3b**, **5**, and **7**

Complex	**1b**	**2a**	**2b**	**3b**	**5**	**7**
Empirical formula	$C_{25}H_{21}N_2O_2P$	$C_{25}H_{32}Cl_2N_3O_2PPd$	$C_{63}H_{73}Cl_7N_6O_4P_2Pd$	$C_{25}H_{20}ClN_2O_2PPd$	$C_{30}H_{24}Cl_3N_2O_2PPd$	$C_{47}H_{37}Cl_5N_4O_4P_2Pd_2$
Formula weight	412.21	614.80	1501.16	553.25	688.23	1173.79
T, K	120	120	120	120	120	120
Crystal system	Triclinic	Monoclinic	Monoclinic	Monoclinic	Triclinic	Triclinic
Space group	$P\bar{1}$	$P2_1/c$	$P2_1/c$	$P2_1/c$	$P\bar{1}$	$P\bar{1}$
Z	2	4	2	4	2	2
a, Å	9.8201(9)	16.9979(10)	9.7726(15)	11.7717(16)	9.6402(19)	10.6639(14)
b, Å	9.8338(9)	9.1189(6)	35.668(5)	11.9075(3)	11.978(2)	13.0578(17)
c, Å	11.9476(10)	18.8822(12)	10.2456(15)	17.389(5)	13.418(3)	17.630(2)
α, °	100.591(2)	90	90	90	74.73(3)	89.676(3)
β, °	108.278(2)	115.7740(10)	113.674(2)	109.346(7)	72.94(3)	73.550(3)
γ, °	102.041(2)	90	90	90	78.15(3)	72.491(2)
V, Å^3	1032.14(16)	2635.6(3)	3270.8(8)	2299.9(7)	1415.1(6)	2236.8(5)
D_{calc} (g cm^{-1})	1.327	1.549	1.524	1.598	1.615	1.743
μ, cm^{-1}	1.58	9.95	9.36	10.18	10.27	12.25
F(000)	432	1256	1532	1112	692	1172
$2\theta_{max}$, °	58	56	54	54	58	54
Reflections measured	21542	28994	21319	8078	7510	9682
Independent reflections	5495	6352	7123	4232	7510	9682
Observed reflections [$I > 2\sigma(I)$]	4480	5154	5037	3433	6652	8437
Parameters	271	310	394	289	353	578
R1	0.0445	0.0319	0.0901	0.0314	0.0451	0.0568
wR2	0.1225	0.0688	0.1638	0.0755	0.1011	0.1974
GOF	1.037	1.021	1.227	1.012	1.058	1.077
$\Delta\varrho max/\Delta\varrho min$ (e Å^{-3})	0.548/–0.248	0.469/–0.775	1.322/–1.163	0.517/–0.367	0.716/–1.078	3.931/–1.930
CCDC	2242547	2242548	2242549	2242550	2242552	2242554
